# *Megavirus baoshanense* Mb0671 modulates host translation and increases viral fitness

**DOI:** 10.3389/fmicb.2025.1574090

**Published:** 2025-04-28

**Authors:** Wenya Bian, Jie Yang, Yucheng Xia, Yun Li, Yanjin Cheng, Yuchen Wu, Jianhua Gan, Jiang Zhong

**Affiliations:** ^1^State Key Laboratory of Genetics and Development of Complex Phenotypes, Department of Microbiology and Immunology, School of Life Sciences, Fudan University, Shanghai, China; ^2^State Key Laboratory of Genetics and Development of Complex Phenotypes, Department of Biochemistry and Biophysics, School of Life Sciences, Fudan University, Shanghai, China

**Keywords:** *Megavirus baoshanense*, Mb0671, translation initiation factor 4A, activity, localization, proteomic, interaction, adaptability

## Abstract

Amoeba giant viruses encode many translation-related proteins, but the function of these proteins remains obscure. In the current work, we studied the potential eukaryotic translation initiation factor 4A (eIF4A, Mb0671) encoded by *Megavirus baoshanense*, a member of the family *Mimiviridae*. The protein was shown to possesse ATPase activity and RNA-binding capacity, localize in the cytoplasm of infected cells, and present in mature virions. Interactome analysis showed that Mb0671 interacted primarily with ribosomal proteins and translation-related proteins. Specifically, Mb0671 was found to interact indirectly with host eIF4A, suggesting that it was associated with the translation apparatus. Proteomic analysis revealed that the protein profile of *Acanthamoeba castellanii* cells stably expressing Mb0671 was altered significantly compared to wild-type cells. The cellular proteins that were significantly upregulated included those in the pathways of spliceosome, amino acids biosynthesis, ribosome biogenesis, vesicular transportation, mTOR signaling pathway, etc. Both Mb0671 overexpression or siRNA-mediated reduction of its expression level significantly affected the synthesis of viral proteins. Furthermore, overexpressing Mb0671 accelerated cell growth and virus replication, whereas reduction of Mb0671 expression by siRNA delayed virus replication. These results suggested that Mb0671 altered cellular translation, possibly through its association with the host translation machinery, and played an important role in enhancing virus adaptability.

## Introduction

The discovery of amoeba giant viruses challenges the clear distinction between viruses and cellular life, due to their extraordinary large size of virions and genomes, and more importantly, their coding of genes that have not been found in virus previously, including genes related to protein translation (Abergel et al., 2015; Brahim Belhaouari et al., 2022; La Scola et al., 2003; Raoult et al., 2004), energy metabolism (Brahim Belhaouari et al., 2022), etc.

The replication of *Mimivirus bradfordmassiliense* (*Acanthamoeba polyphaga* mimivirus, APMV), the first amoeba giant virus isolated, has been studied in detail (Colson et al., 2017). The virus first enters amoeba cells by phagocytosis, and after the fusion of the phagosome with lysosome (1 to 2 h post-infection, p.i.), the stargate on the virion is opened and the viral DNA is released around 4 h p.i. A typical viral eclipse phase is then established, during which no viral particle is visible in the cell. An early virus factory (VF) is formed and viral proteins are synthesized outside the factory 4–6 h p.i. In a mature VF, newly assembled virions increase in complexity and acquire the genomes and fibrils simultaneously (8 h p.i.). Finally, the viral progeny is released after cell lysis (Andrade et al., 2017).

One uncommon feature of amoeba giant viruses is that they encode many translation-related genes in their genomes. APMV encodes four aminoacyl tRNA synthetases (aaRS), six tRNAs, and five translation factors (Abrahão et al., 2017). Member of the genus *Tupanvirus* in the family of *Mimiviridae*, along with APMV, possesses the most complete translational apparatus of the known virosphere, with up to 70 tRNA, 20 aaRS, 11 factors for all translational steps, and factors related to tRNA maturation and ribosome protein modification (Abrahão et al., 2018; Aylward et al., 2023). The virus under study in the current work, *Megavirus baoshanense* (M. baoshan), from the genus *Megavirus*, *Mimiviridae*, shared similar dynamics of morphogenesis with APMV and other mimiviruses (Xia et al., 2022a). Its 1.22 Mb genome encoded nine tRNAs and 1,062 predicted ORFs (Xia et al., 2022b), including seven aaRS (TyrRS, TrpRS, CysRS, ArgRS, MetRS, AsnRS, IleRS), one tRNA^His^ guanylyltransferase, and five potential translation factors (eIF4A, eIF4E, SUI1, EF-Tu, eRF1).

The finding of translation-related genes in giant amoeba viruses have sparked widespread discussion (Schulz et al., 2017), though the function of these genes in protein translation remains to be elucidated. Bekliz et al. showed that silencing APMV eIF4A slowed the replication of virus, but viral production at the end of the infection was unaffected, and a 2-D gel-based proteomic analysis showed deregulation of 32 viral proteins (Bekliz et al., 2018). More researches are needed to understand the role of these unusual viral genes in virus infection and adaptability.

In eukaryotic cell, protein translation can be divided into four main stages: initiation, elongation, termination and ribosome recycling (Brito Querido et al., 2024), with translation initiation being the most regulated and also the most varied one (Montero et al., 2019). The eIF4A is an essential component of the initiation complex eIF4F, and is responsible for the unwinding of the secondary structures within the 5′-untranslated region of mRNA and recruiting ribosome to attach to mRNA (Montero et al., 2019). eIF4A belongs to the DEAD-box family and possesses RNA-dependent ATPase activity, RNA binding capability, and helicase activity (Ray et al., 1985; Rogers et al., 2002; Rogers et al., 2001; Rogers et al., 1999).

Viruses rely on host translational machinery to produce viral proteins. Many of them have evolved mechanisms to prioritize viral protein synthesis over that of the host (Montero et al., 2015; Montero et al., 2019). The eIF4F complex, including eIF4A, is frequently targeted by viruses for this purpose, though the specific strategies employed by different viruses vary widely, with some utilizing eIF4A and others not, or even toggling between dependence and independence on it (Bushell and Sarnow, 2002; Gale et al., 2000; Jan et al., 2016; Smith and Gray, 2010; Walsh, 2010). Some viruses manipulate the eIF4A protein to increase viral protein synthesis during infections (Bushell and Sarnow, 2002; Gale et al., 2000; Jan et al., 2016; Montero et al., 2015; Smith and Gray, 2010; Walsh, 2010).

M. baoshan protein Mb0671 was annotated to be eukaryotic translation initiation factor eIF4A. In the current work, we showed that Mb0671 protein has ATPase and RNA binding activities *in vitro*. In either infected or non-infected *A. castellanii* cells, Mb0671 interacted, directly or indirectly, with host cell translation-related proteins such as ribosomal proteins and translation factors. Overexpression of Mb0671 significantly changed cellular protein composition, accelerated cell proliferation, and significantly sped up viral replication, particularly in early infection stages. These results indicated that Mb0671 markedly influenced or manipulated the protein synthesis machinery in the host cell, and played an important role in virus replication and its adaptability to the host.

## Materials and methods

### Cell culture, virus particle production and purification

*A. castellanii* str. Neff (ATCC 30010) cells were cultured in peptone-yeast extract with glucose (PYG) medium in a T-150 flask at 28°C using standard techniques (Abrahão et al., 2016).

M. baoshan (GenBank number: MH046811.2) was propagated in *A. castellanii* cells as described (Xia et al., 2022a). Briefly, the virus was inoculated to the cell of approximately 95% confluence at an MOI of approximately 1, and the flask was gently swirled for even distribution. The culture was incubated at 28°C until cytolysis occurred, typically within 4 days. The lysate was collected into a 50 mL tube, freeze-thawed three times at −80°C to release viruses, and centrifuged at 4°C, 500 × g for 5 min to remove cell debris. The supernatant was either stored at −80°C or used for purification.

The virus-containing supernatant was filtered through a filter of 1.2-μm pore size and centrifuged at 12,000 × g for 30 min. The virus pellet was resuspended in 10 mL PBS, washed three times with PBS, and layered on a discontinuous sucrose gradient (20, 30, 40, 50, 60, 70%; wt/vol). The gradient was centrifuged at 32,000 × g for 8 h. The band of virions was collected, washed three times with PBS buffer, and stored at −80°C or used for proteomic analysis.

Marseillevirus Shanghai 1 (GenBank number: MG827395.1) was propagated in the same way.

### Mb0671 sequence alignment and conserved domain prediction

BLAST searches were conducted with the amino acid sequence of Mb0671, and homologous proteins were selected from different categories of organisms including fungi, metazoa, bacteria, archaea, plants, protists, and viruses. In the BLAST search, selected Mb0671 homologous proteins had *e*-values of ≥2E-52, coverage of ≥74%, positives of ≥47%, and identities of ≥29%. One virus from each lineage within *Megamimivirinae* was chosen for phylogenetic tree construction. For protists, the protein with the highest homology to Mb0671 was selected from each group, along with eIF4A from *A. castellanii*. For other organisms, the proteins with the highest homology to Mb0671 were selected from each category.

Multiple alignment was performed with HHpred’s Multiple Alignment using Fast Fourier Transform (MAFFT) with a 1.53 gap open penalty. Maximum likelihood phylogenetic tree was constructed using RaxML (Stamatakis, 2014), with 1,000 bootstrap replicates and using the JTT protein substitution model. The constructed phylogenetic tree was visualized using the online website Chiplot (Xie et al., 2023). The phylogenetic tree of Mb0671 with homologous proteins from other members of *Megamimivirinae* was built similarly, using the homologous protein from *Fadolivirus* of the *Klosneuvirinae* as an outgroup. Both *Megamimivirina* and *Klosneuvirinae* belong to the family *Mimiviridae* (Aylward et al., 2023).

### Construction of overexpression cell lines

The PCR primers used in the work were listed in Supplementary Table S1A. The Mb0671 encoding gene of M. baoshan was amplified by PCR from viral genomic DNA and cloned into the pGAPDH-GFP amoebal expression plasmid, which had a neomycin resistant gene, to obtain the pGFP-Mb0671 plasmid that expressing Mb0671 fused with GFP at the N-terminal (Bateman, 2010). The eIF4A-encoding gene of *A. castellanii* (GenBank number GCA_000313135.1) was amplified by PCR from genomic DNA of the cell. The forward primer used in PCR had additional sequence encoding a HA-tag added to its 5′ end and the PCR product was cloned into the pGAPDH-GFP similarly, to obtain the plasmid pHA-AceIF4A that expressing N-terminally HA-tagged AceIF4A (Bateman, 2010).

The Mb0671 and GFP genes, both fused in frame with the FLAG-tag sequence at their 5′ ends, were cloned into the modified amoebal expression plasmid pEF1-GFP-NAT, which encoded a Nourseothricin resistance gene (Liu et al., 2021), to obtain the plasmids of pFLAG-Mb0671 and pFLAG-GFP, respectively. They were used to express N-terminally FLAG-tagged Mb0671 and GFP.

The pFLAG-Mb0671 plasmid was used to transfect *A. castellanii* cells. 1 × 10^6^ *A. castellanii* cells were transfected with 6 μg plasmid using the Polyfect transfection reagent (Qiagen, Germany) following standard procedures, and the transfected cells were selected with nourseothricin. The concentration of nourseothricin was 30 mg/mL at the beginning, and increased up to 60 mg/mL over a couple of weeks, resulting in cells stably expressing the FLAG-tagged Mb0671 (Ac_Mb0671).

The pGFP-Mb0671 and pHA-AceIF4A plasmids were used to transfect *A. castellanii* cells in the same way, and the transfected cells were selected with 30 mg/mL G418 at the beginning, and the concentration of G418 increase up to 60 mg/mL. Cells stably overexpress GFP-fused Mb0671, and HA-tagged AceIF4A were obtained and named Ac_GFPMb0671 and Ac_HAAceIF4A, respectively.

To obtain cells overexpress both Mb0671 and AceIF4A, or both GFP and AceIF4A, Ac_HAAceIF4A cells were transfected with plasmids FLAG-Mb0671 or FLAG-GFP, followed by selection with 30 ~ 60 mg/mL Nourseothricin as above. After the cell growth in 60 μg/mL nourseothricin became stable, 60 μg/mL G418 was added. Ultimately, dual-overexpressing cells (Ac_FGFP+HAAceIF4A or Ac_FMb0671 + HAAceIF4A) were obtained.

### Fluorescence and immunofluorescence microscope

Cells were cultured in confocal microscopy-specific dishes and infected with M. baoshan at an MOI of 50, except for the negative control. The culture medium was removed 6 h p.i., and the cells were washed once with PBS, then fixed with PBS containing 3.7% formaldehyde for 30 min at room temperature. To visualize GFP-fused protein (Ac_GFPMb0671), the fixed cells were washed once with PBS, followed by the addition of 50 μL VECTASHIELD mounting medium with DAPI (4′,6-diamidino-2-phenylindole; Vectorlabs, Burlingame, United States) to the center of the culture dish. For immunofluorescence experiments (Ac_HAAceIF4A), the fixed cells were washed three times with PBS and then incubated with Immunol Staining Blocking Buffer (Beyotime, Shanghai, China) at room temperature for 60 min. After washing three times with Immunol Staining Wash Buffer (Beyotime), the cells were incubated with a 1:100 diluted HA-tag mouse monoclonal antibody (Beyotime) at room temperature with gentle shaking for 1 h. The primary antibody was removed, and the cells were washed with Immunol Staining Wash Buffer for 5 min with gentle shaking. After the wash buffer was removed, the cells were incubated with a 1:500 diluted Cy3-labeled goat anti-mouse IgG (H + L) (Beyotime) at room temperature with gentle shaking for 1 h. The cells were then washed with Immunol Staining Wash Buffer for 5 min with gentle shaking. Finally, 50 μL of VECTASHIELD mounting medium with DAPI was added to the center of the culture dish.

The fluorescence was examined using a Zeiss LSM880 with Airyscan super-resolution laser scanning confocal microscope with a 63x objective lens associated with a 1.6x Optovar for DAPI, GFP, and Cy3 fluorescence recording.

### RNA extraction and RT-qPCR

Total cellular RNA was extracted using the RNAsimple Total RNA Kit with gDNA wiper (Tiangen Biotech, Beijing, China). The cDNA was synthesized from the total RNA using the HiScript III RT SuperMix for qPCR (Vazyme, Nanjing, China), according to the manufacturer’ instructions. The primers were designed using the Primer-BLAST (Ye et al., 2012), and are listed in Supplementary Table S1B. RT-qPCR was performed using Taq Pro Universal SYBR qPCR Master Mix (Vazyme) on a Bio-Rad CFX96 Touch real-time PCR system. The mRNA level of target gene was normalized against *A. castellanii* mitochondrial cytochrome b gene (NCBI accession number: AOS85701) using the threshold cycle (2^−ΔΔCT^) method (Li et al., 2022; Xia et al., 2022a).

### Mass spectrometry proteomic

The purified virion of M. baoshan was lysed by adding 50 mM Tris–HCl (pH 7.5), 2% SDS solution, and 10 mM DTT to it. The protein concentration was determined using the BCA method. The resulting protein samples were subjected to LC-MS/MS.

*A. castellanii* cells were infected with M. baoshan (MOI = 20). After 1 h, the medium was removed, and the cells were washed three times with PBS, and fresh PYG medium was added. The infected cells were cultured at 28°C before being collected at 4 and 9 h p.i. with three replicates for each time point. They were then lysed with RIPA cell lysis buffer (Epizyme, Shanghai, China), and the concentration of protein was determined as above and the resulting protein samples were subjected to label-free quantitative proteomics analysis via LC–MS/MS. For uninfected cells, similar cell lysis and quantification methods were used.

The FASP (Filter-Aided Sample Preparation) digestion was adapted following procedures in Microcon PL-10 filters. After three-time buffer displacement with 8 M Urea and 100 mM Tris–HCl, pH 8.0, proteins were reduced by 10 mM DTT at 37°C for 30 min, followed by alkylation with 30 mM iodoacetamide at 25°C for 45 min in dark. Then, three rounds of buffer displacement were done with digestion buffer (30 mM Tris–HCl, pH 8.0), and the digestion was carried out with trypsin (enzyme:protein = 1:50) at 37°C for 12 h. After digestion, the solution was filtrated and the filter was washed twice with 15% acetonitrile (ACN), and all the filtrates were pooled and vacuum-dried. LC–MS/MS analysis was performed using a nanoflow EASYnLC 1200 system (Thermo Fisher Scientific, Odense, Denmark) coupled to an Orbitrap Exploris480 mass spectrometer (Thermo Fisher Scientific, Bremen, Germany). A one-column system was adopted for all analyses. Samples were analyzed on a home-made C18 analytical column (75 μm i.d. × 25 cm, ReproSil-Pur 120 C18-AQ, 1.9 μm, Dr. Maisch GmbH, Germany). The mobile phases consisted of Solution A (0.1% formic acid) and Solution B (0.1% formic acid in 80% ACN). The peptides for LFQ analysis were eluted using the following gradients: 5–8% B in 3 min, 8–44% B in 100 min, 44–70% B in 5 min, 70–100% B in 2 min, 100% B for 10 min, at a flow rate of 200 nL/min. High-field asymmetric-wave form ion mobility spectrometry (FAIMS) was enabled during data acquisition with compensation voltages set as −40 and −60 V. MS1 data were collected in the Orbitrap (60,000 resolution). Charge states between 2 and 7 were required for MS2 analysis, and a 45 s dynamic exclusion window was used. Cycle time was set at 1.5 s. MS2 scans were performed in the Orbitrap with HCD fragmentation (isolation window 1.6; 15,000 resolution; NCE 30%, max injection time 30 ms).

The raw data from the mass spectrometer were imported into ProteomeDiscoverer^™^ Software (version 2.4, Thermo Fisher Scientific) and searched against the Mascot search engine (version 2.7.0, Matrix Science) for processing. The protein database for *A. castellanii* and M. baoshan used in this study was downloaded from UniProt (UP0000110833) and NCBI (MH046811.2). The mass tolerances were 10 ppm for precursor and fragment Mass Tolerance 0.05 Da. Up to two missed cleavages were allowed. The carbamidomethylation on cysteine was set as a fixed modification, and acetylation on the protein N-terminal and oxidation on methionine were set as variable modifications. Identified proteins must contain at least one unique peptide.

Data are available at the ProteomeXchange Consortium (proteomecentral.proteomexchange.org) via the iProX partner repository with the accession code PXD056594.

### Transcriptomics

The transcription dynamics of translation related gene was analyzed based on transcriptome data of *A. castellanii* cells infected by M. baoshan published in literature (Xia et al., 2022a). The accession numbers of RNA-seq data in Sequence Read Archive (SRA) database, NCBI, is PRJNA778649. Expression values were expressed in log transformed counts per million of mapped reads (CPM). For the 5 translation initiation factors, 7 aaRS, and one tRNA^His^ guanylyltransferase encoded by Mb0671, as well as the corresponding homologous proteins and actin proteins encoded by *A. castellanii*, each transcript expression value was scaled to a Z-score to produce a heatmap using the online OmicShare Heatmap tools.^1^

Ac and Ac_Mb0671 cells were used to extract total RNA, and the RNA samples were proceeded with transcriptome sequencing and data processing, followed by differential gene expression analysis as described in (Xia et al., 2022a). The RNA-seq data have been submitted to the NCBI’s Sequence Read Archive (SRA) database and the accession numbers is PRJNA1195399.

### Purification of his-Mb0671, his-Mb0678, and HA-AceIF4A proteins

The primers used for PCR are listed in Supplementary Table S1C. M. baoshan genes of Mb0671 and Mb0678 were amplified by PCR and cloned into the pET-28a plasmid in-frame with the N-terminal His-tag sequence for protein expression. His-tagged proteins of Mb0671 and Mb0678 were purified from the culture of recombinant *E. coli*. Similarly, the N-terminal HA-tagged AceIF4A were cloned into the pETSUMO plasmid, and the recombinant protein with HA-tag was purified.

The entire process of protein purification was carried out on ice or at 4°C. 1 L *E. coli* cells were resuspended and lysed with 100 mL lysis buffer (0.2 mM DTT, 500 mM NaCl, 20 mM Tris–HCl, pH7.5) by using an APV Laboratory Homogenizers (SPX FLOW), under 1,000–1,500 bar pressure for 3–4 cycles. Right before purification, cOmplete EDTA-free Protease Inhibitor Cocktail (Roche, Basel, Switzerland) was added into the lysis buffer, and the cell lysate was centrifuged at 15,000 × g for 30 min. The supernatant was incubated with Ni-NTA Agarose Beads (HisTrap™ HP, 5 mL; Cytiva, Marlborough, United States) at 4°C for 1 h. Then the Ni-NTA resin was washed with 100 mL wash buffer A (20 mM imidazole, 5% Glycerol, 0.2 mM DTT, 1 M NaCl, 20 mM Tris–HCl, pH7.5), and proteins were eluted with a gradient of wash buffer A and wash buffer B (500 mM imidazole, 5% glycerol, 0.2 mM DTT, 1 M NaCl, 20 mM Tris–HCl, pH 7.5). The eluate was collected into Slide-A-Lyzer™ G3 Dialysis Cassettes with a 3.5 K MWCO (Thermo Fisher Scientific, Waltham, United States), and dialyzed in 4 L of dialysis buffer (0.5 M NaCl, 5% glycerol, 0.2 mM DTT, 20 mM Tris–HCl, pH 7.5, MilliQ water) for 16 h.

When purifying HA-tagged AceIF4A expressed with pETSUMO, ubiquitin-like-specific protease 1 (Ulp1) was added to the Dialysis Cassettes during the first dialysis to cleave off the SUMO-tag. After dialysis the protein was passed through Ni-NTA resin again, and the flow-through was collected for another round of dialysis.

DTT was added to the purified protein solution to the final concentration of 5 mM, and the protein was concentrated to approximately 1 mg/mL, using a 30 kDa MWCO Amicon Ultra-15 Centrifugal Filter Unit (Millipore, Boston, United States) and centrifuge at 3,000 × g and 4°C. The concentrated protein was aliquoted and frozen with liquid nitrogen before storing at −80°C. The purification of Ulp1 protease followed the method described in literature (Chen et al., 2022).

### RNA-crosslinking and ATPase activity assays

For RNA-crosslinking assay, Mb0671 protein (0, 2, 3, and 4 μM) was incubated with 2 μM 5′-FAM-labeled 15-mer poly(U) RNA (Supplementary Table S1D) in the buffer of 2 mM AMPPNP, 20 mM Tris–HCl at pH 7.5, 50 mM NaCl, 1 mM TCEP and 5 mM MgCl_2_ at room temperature for 30 min. The sample was then irradiated with UV light at 254 nm for 30 min, before being resolved on a 4–12% polyacrylamide gel and visualized using the Typhoon FLA 9000 Gel Imaging Scanner (GE healthcare, Boston, United States).

ATPase activity was assayed using EnzChek phosphate assay kit (Invitrogen, Carlsbad, United States). Mb0671 protein was diluted to the concentration of 1 μM in a buffer containing 20 mM Tris–HCl at pH 7.5, 2 mM ATP, 1 mM TCEP, and 5 mM MgCl_2_. The assay was carried out at 37°C following manufacture’s protocol. Assay conditions included both the presence and absence of RNA, with RNA samples consisting of 0.2 mg/mL poly(U)/poly(C) RNA (Sigma-Aldrich, Saint Louis, United States). Each data point was derived from three independent reactions. The ATPase activity of Mb0678 was measured under the same conditions (absence of RNA) as a negative control.

### Cell proliferation curve and viral replication curve

Cells of wild type *A. castellanii*, Ac_Mb0671, Ac_AceIF4A, or Ac_Mb0671 + AceIF4A were seeded in φ60 mm cell culture dishes, 1.25 × 10^5^ cells/dish, and cultured as described above. Cell numbers were counted at days 1, 2, 3, and 4. Three independent experiments were carried out.

In the virus replication experiments, these cells were infected with M. baoshan or Marseillevirus Shanghai 1 (MOI = 10). After 1 h of infection, the culture medium was removed, and cells were wash three times with PBS. Fresh PYG medium was added. Cells were collected 1 h, 4 h, 9 h, 16 h, and 24 h p.i., and freeze-thawed at −80°C for three times to release the viruses. End-point dilution assays were performed and the viral titer (TCID50/mL) was calculated as described (Jacinto et al., 2004; Németh et al., 2018). Three independent experiments were carried out.

### Polyhistidine protein–protein pulldown

The Pierce Pull-Down PolyHis Protein:Protein Interaction Kit (Thermo Fisher Scientific, Waltham, USA) was used to perform the polyhistidine protein–protein pulldown assays following the manufacturer’s protocol. 150 μg of purified bait protein (His-Mb0671) is dissolved in wash solution to a final volume of 600 μL and incubated with HisPur Cobalt Resin at 4°C for 1 h. After being washed five times with wash solution (TBS with Pierce lysis buffer, 1:1 vol/vol), the resins were incubated with cell lysates of *A. castellanii* (NI; 6 h p.i., MOI = 10) for 2 h at 4°C, followed by washing for five times with wash solution. Elution solution was added to the resin to recover bait-prey proteins, which were then analyzed by LC–MS/MS.

To investigate whether Mb0671 directly interacts with AceIF4A, 150 μg of purified HA-AceIF4A protein was incubated with the immobilized bait protein His-Mb0671. The eluted bait-prey proteins were then analyzed by SDS-PAGE followed by immunoblot.

### Co-IP

Two T-75 flasks of either non-infected (NI) or M. baoshan-infected (6 h p.i., MOI = 10) *A. castellanii* cells were collected and washed three times with 1 mL PBS. DYKDDDDK IP/Co-IP Kit for Co-IP experiments (Epizyme, Shanghai, China) were used for the experiment. The cell pellet was resuspended in 500 mL lysis/wash buffer with 1 mM PMSF as described in the instructions and left on ice for 20 min. After centrifugation at 12,000 g for 10 min at 4°C to remove debris, the supernatant was incubated with anti-FLAG antibody-coated beads for 1 h at room temperature with mixing. The protein-bead complexes were then washed five times with 500 mL lysis/wash buffer and separated using a magnetic separator. After elution with elution buffer, eluted proteins were subsequently analyzed by LC–MS/MS, SDS-PAGE, and immunoblot.

### Bioinformatic analysis of proteomic data

Principal component analysis (PCA) and Venn diagrams construction utilized the OmicShare tool,^2^ and default values were used. Differential expression analysis was performed using edgeR (Richardson et al., 2004) in the OmicShare tools with default parameters, and differential expression proteins were selected according to |Log2(Fold Change)| > 1 and *p*-value < 0.05. The functions of all proteins encoded by *A. castellanii* and M. baoshan were annotate separately, using eggNOG-mapper.^3^ The annotation files were organized using the eggNOG-mapper Helper feature in TBtools (Chen et al., 2023). Finally, Gene Ontology (GO) enrichment and Kyoto Encyclopedia of Genes and Genomes (KEGG) enrichment analysis were performed using the background protein files for *A. castellanii* and M. baoshan, together with the mass spectrometry data of proteome, and the results were visualized using the OmicShare tool.

### siRNA

*A. castellanii* cells were seeded to confluence in wells of a 12-well culture plate. The medium was aspirated, and 1 mL of fresh PYG medium was added to each well. Transfection reagent (5 μL, DWS™ transfection reagent; Wishtech, Changchun, China) and siRNA (4 μL, 20 μM; synthesized by Genepharma, Shanghai, China, Supplementary Table S1E) was added to two separate tubes with PYG medium to the volume of 50 μL each, and the tubes were vortex for 10 s. Then the two solutions were combined and incubated at room temperature for 15 min. The combined mixture was then added to the wells, with gentle rocking of the plate to ensure even distribution. The cells were then incubated at 28°C. After 4 h, the transfection mixture was removed, and cells were washed three times with PBS. For wells transfected with FAM-NC siRNA, PYG medium were removed and the cells were washed five times with PBS. The transfection efficiency was examined under an Olympus CKX41 microscope with a 40x objective lens and a fluorescence attachment. For cells transfected with siRNA, fresh PYG medium was added to the washed cells together with M. baoshan virions to achieve an MOI of 10. The cells were then incubated in 28°C incubator for 1 h for virus infection. Then, the medium was removed and the cells were washed three times with PBS. Fresh PYG medium were added and the culture were further incubated at 28°C. Infected cells were collected 1 h, 4 h, 9 h, and 16 h p.i., and used for RNA extraction and RT-qPCR experiments, or used for viral titers assay as described above. Control group transfected with NC-siRNA for each time point were set up, with three replicates for each time point. Cells infected for 4 h (MOI = 10) (with Mb0671-355 or NC siRNA) were also collected, with three replicates each, for label-free quantitative proteomics analysis.

## Results

### Phylogenetic and conserved domain analysis of Mb0671

**Figure 1:**
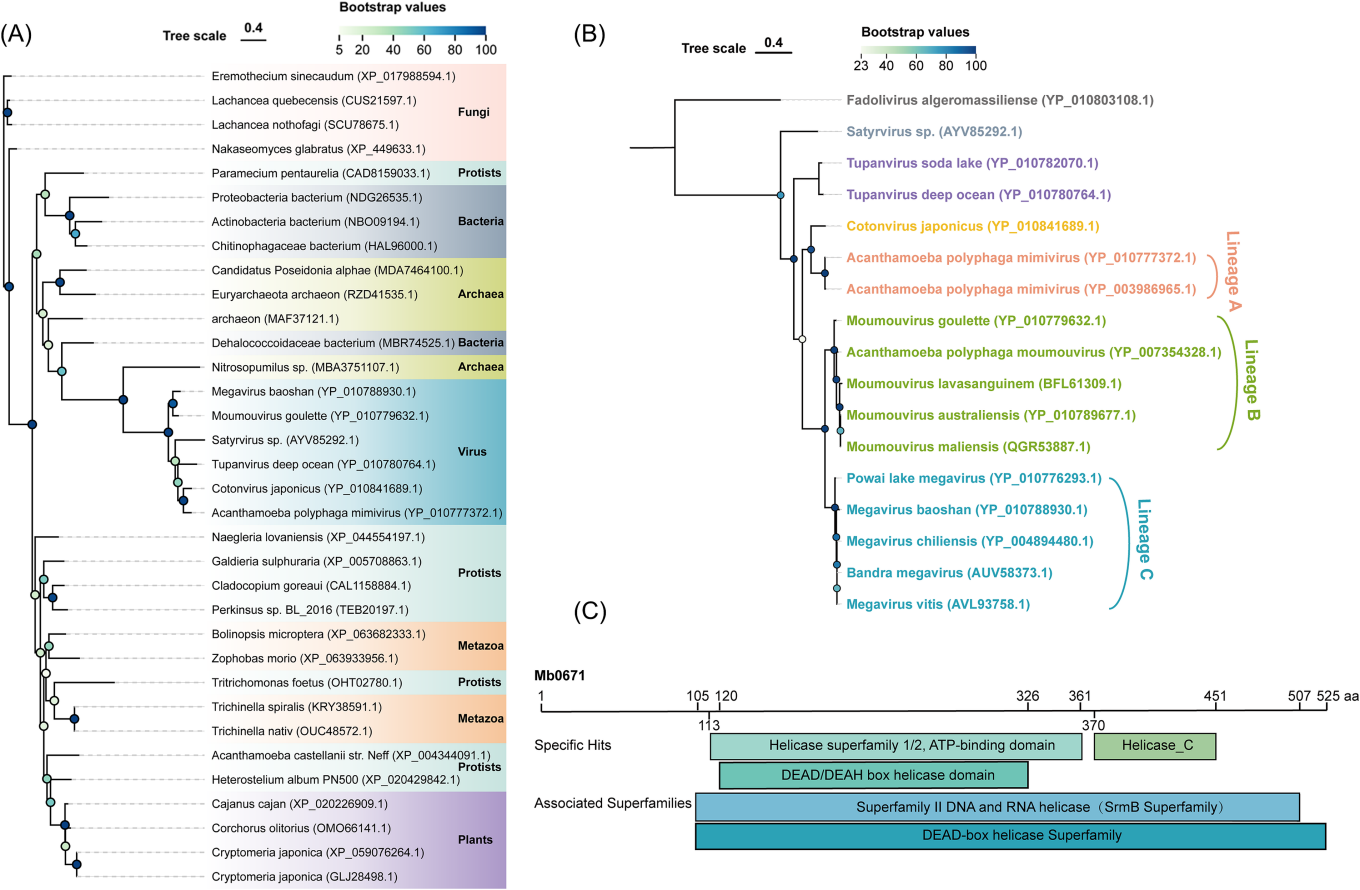
Phylogenetic and conserved domain analysis of Mb0671. **(A)** Maximum likelihood phylogenetic tree of Mb0671 gene and its homologs from fungi, protists, bacteria, archaea, metazoa, plants, and viruses. The tree was built using RaxML (Stamatakis, 2014). **(B)** Phylogenetic tree based on an alignment of the amino acid sequences of Mb0671 and its homologous proteins from members of the *Megamimivirinae*, using Fadolivirus of the *Klosneuvirinae* as an outgroup. **(C)** Conserved domains in Mb0671 and its associated superfamily. Helicase_C: helicase conserved C-terminal domain. Genebank ID was indicated after the name of each organism.

M. baoshan Mb0671 is a protein of 525 amino acids, and annotated to be eukaryotic translation initiation factor 4A (eIF4A). A phylogenetic analysis of homologous proteins from different type of organisms revealed that Mb0671 clustered together with other mimiviral eIF4A, and had a close evolutionary relationship with proteins from archaea, followed by bacteria (Figure 1A). Instead, eIF4A from its host, *A. castellanii*, was situated in a different branch together with plants and metazoans. This excluded the possibility that Mb0671 originated from horizontal gene transfer from the host. High sequence homology was seen among viral eIF4A proteins within the *Megamimivirinae* at the amino acid level (Figure 1B), and conserved DEAD/DEAH box helicase domain and helicase conserved C-terminal domain were predicted in these proteins (Figure 1C), similar to cellular eIF4A. It was suggested that Mb0671 belongs to the SrmB superfamily/DEAD-box helicase superfamily, possessing ATPase, RNA binding, and helicase activities. Sequence alignment of Mb0671 and *A. castellanii* eIF4A (AceIF4A) was shown in Supplementary Figure S1. Relatively high level of homology was seen in the region between amino acids 56 and 525 of Mb0671, which was predicted to contain all thirteen conserved motifs of the DExD-box helicase family (Chen et al., 2020; Jankowsky and Putnam, 2010; Linder and Jankowsky, 2011). It was speculated that in addition to some functions similar to AceIF4A, Mb0671 may also possess other functions.

### Expression of Mb0671 during infection

**Figure 2:**
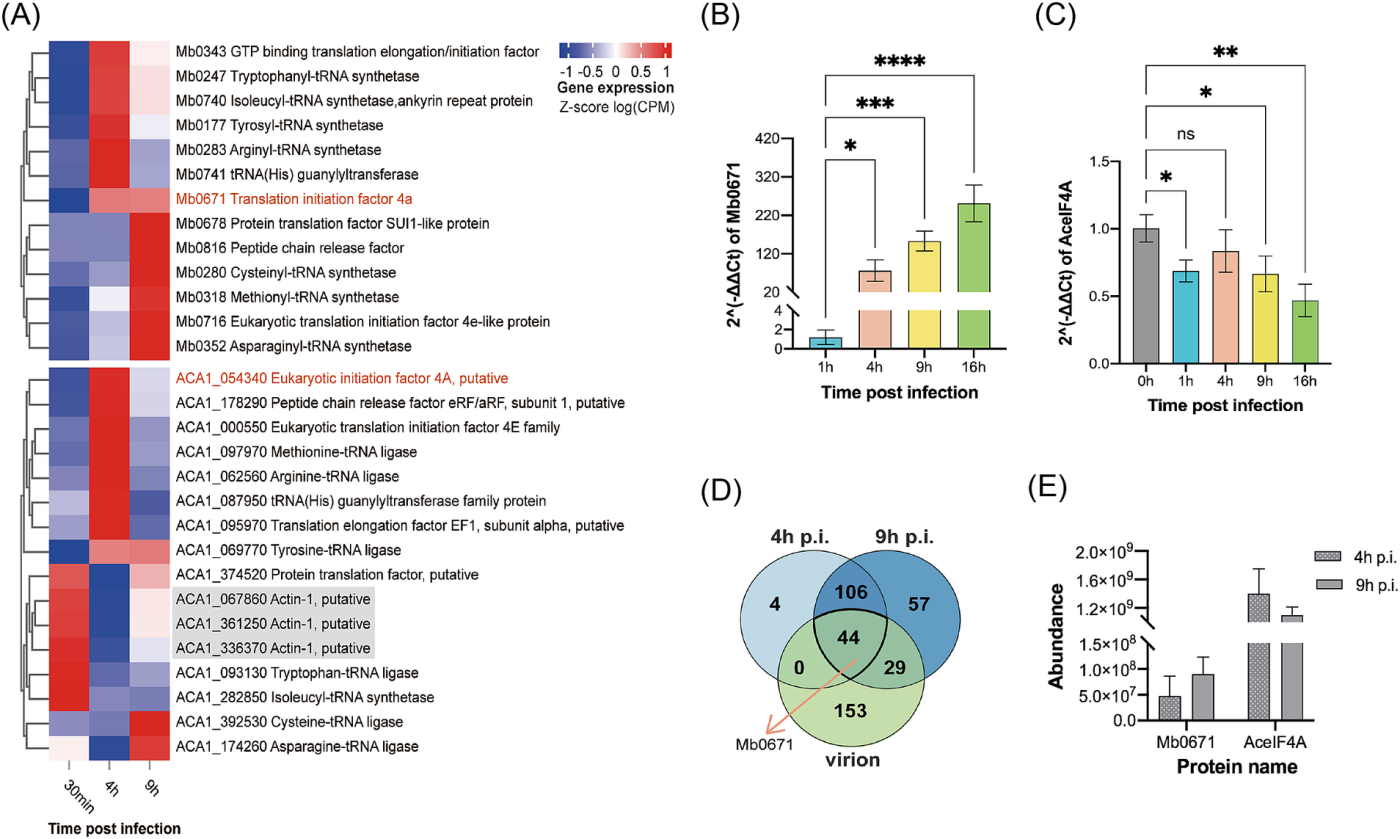
Expression profile of translation-related proteins of M. baoshan and their host homologs. **(A)** Transcription profile of translation-related genes encoded by M. baoshan and *A. castellanii*, 30 min, 4 h, and 9 h p.i., based on RNA-seq data using the Euclidean Clustering method. Host actin genes were used as reference. **(B,C)** Expression level of Mb0671 **(B)** and AceIF4A **(C)** genes based on RT-qPCR. **(D)** Statistics of proteins present in virions, infected cells 4 h and 9 h p.i. The statistical counts of protein identified by LC–MS/MS in virus-infected cells 4 h and 9 h p.i., as well as in the purified virions, were displayed in the Venn diagram showing the intersections and subsets of these three datasets. **(E)** Relative protein abundance of Mb0671 and AceIF4A determined through label-free quantitative proteomics at 4 h and 9 h p.i. Student’s *t* test was performed to assess the significances of the difference between groups in **(B,C)**. *p ≤ 0.05; **p ≤ 0.01; ***p ≤ 0.001; ****p < 0.0001; ns: *p* > 0.05.

Transcriptome sequencing (RNA-seq) were performed to show the expression of 13 translation-related genes encoded by M. baoshan and their host homologs in infected *A. castellanii* cells. All predicted 13 viral genes were found in the transcriptome. Mb0671 began to express at 4 h p.i., and its expression level increased with infection. For other virus-encoded translational factors, SUI1-like protein and peptide chain release factor did not highly expressed until 9 h p.i., and others began to express 4 h p.i., with some peaking in expression at 4 h p.i. and others at 9 h p.i. (Figures 2A,B).

Unlike most of the *A. castellanii* genes (for example, the actin genes in gray), which had decreased expression levels after M. baoshan infection, the host eIF4A gene (AceIF4A), along with the majority of host translation-related genes, showed an increase in expression levels at 4 h p.i., and then decreased by 9 h p.i. to levels slightly higher than those at 30 min p.i. (Figures 2A,C).

Similar dynamics of transcription of Mb0671 and AceIF4A was seen using RT-qPCR (Figures 2B,C), where the mRNA level of Mb671 kept increasing up to 16 h p.i., and that of AceIF4A increased 4 h p.i. and decreased late in the infection. The level of viral proteins was also determined using label-free quantitative proteomic analysis. A total of 226, 154, and 236 proteins were identified in virions, infected cells collected at 4 h and 9 h p.i., respectively (Supplementary Tables S2A–C). A total of 44 proteins were present in all three samples (Figure 2D; Supplementary Table S2D), and Mb0671 was among them. Mb0671 was also the only virus-encoded translation-related protein presented in the virions, though at low abundant. In infected cells, the level of Mb0671 protein increased significantly from 4 h to 9 h p.i., while the level of its host homolog AceIF4A remained almost unchanged, though the latter was consistently 10–20 times more abundant than Mb0671 (Figure 2E).

### Cellular localization of Mb0671

**Figure 3:**
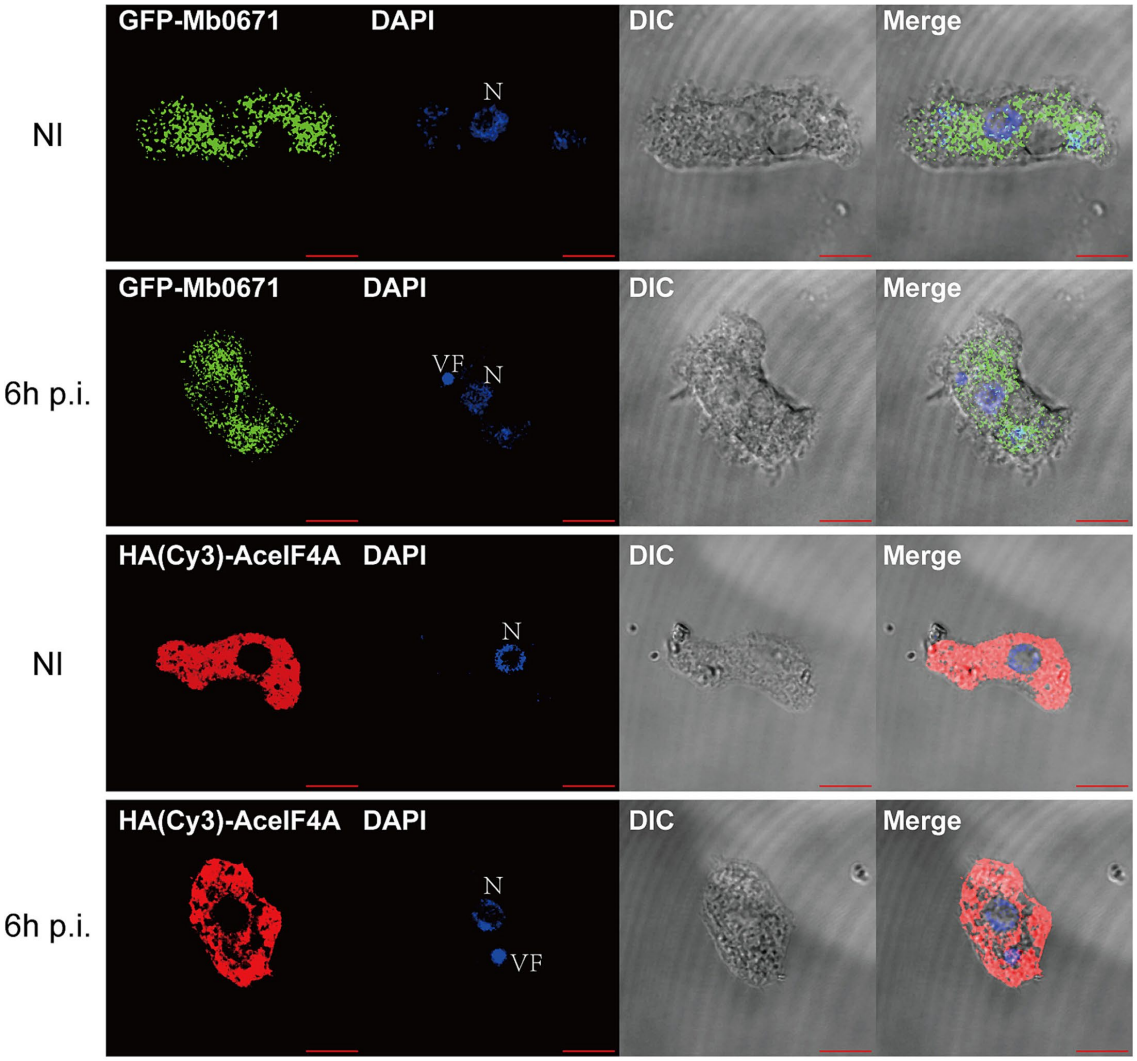
Localization of Mb0671 and AceIF4A in *A. castellanii*. Confocal fluorescence microscopy of *A. castellanii* cells stably express GFP-Mb0671 or HA-AceIF4A, either non-infected (NI) or infected with M. baoshan 6 h p.i. HA-tagged Mb0671 were stained with anti-HA mouse mAB (1:100) and Cy3-Goat anti-Mouse IgG (H + L) (1:500). DAPI staining was used shows the cell nucleus (N) and viral factories (VF). Scale bar: 10 μm.

To determine the localization of Mb0671 in cells, we transfected *A. castellanii* cells with plasmid expressing Mb0671 fused with green fluorescent protein (GFP-Mb0671), as well as Hemagglutinin (HA)-tagged amoeba eIF4A (HA-AceIF4A). In immunofluorescent microscopy (Figure 3), strong DAPI staining (blue) of virus factory can be seen in the cytoplasm, consistent with previous reports (Mutsafi et al., 2013; Suzan-Monti et al., 2007). In both infected and uninfected cells, both GFP-Mb0671 and HA-AceIF4A distributed in the cytoplasm, not in the nucleus, nor viral factories (Figure 3).

### ATPase and RNA binding activities of Mb0671

**Figure 4:**
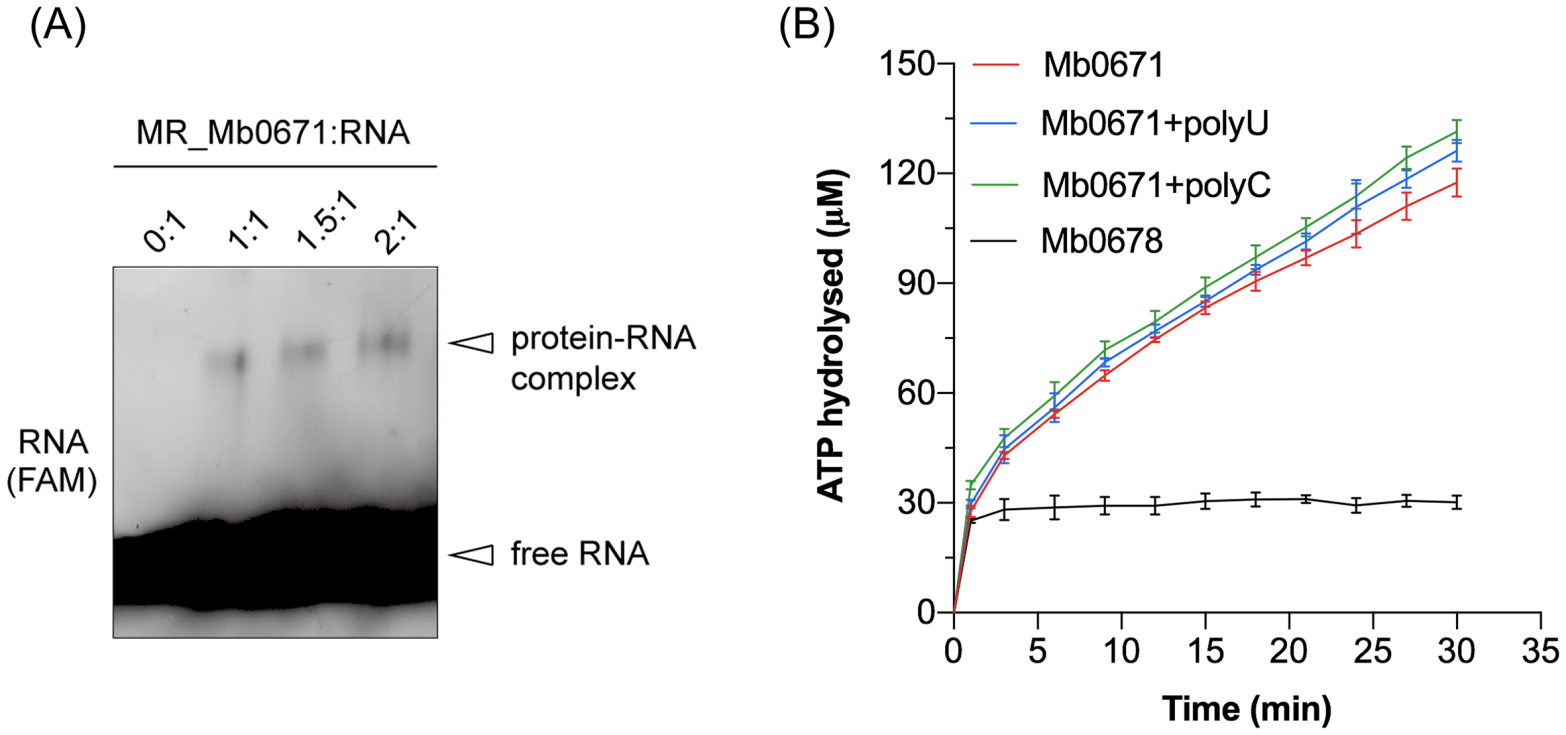
RNA binding and ATPase activity of Mb0671. **(A)** Crosslinking of Fam-labeled RNA with Mb0671. The RNA-crosslinking assay was carried out by incubating 2 μM 5′-Fam labeled 15-mer poly(U) RNA with 2 μM, 3 μM, and 4 μM Mb0671, respectively, in buffer containing 2 mM AMPPNP and 5 mM MgCl_2_ at room temperature for 30 min, followed by irradiation with a 254 nm UV lamp for another 30 min. Samples were then subjected to SDS-PAGE on a 4–12% polyacrylamide gel. MR: molar ratio. **(B)** ATPase activities of Mb0671. The OD_360_ readouts were converted to the concentrations of hydrolyzed ATP by a standard curve for inorganic phosphate (not shown).

Purified Mb0671 protein was prepared using prokaryotic expression system, and its activities were assayed *in vitro*. The RNA-crosslinking assay confirmed that Mb0671 had RNA-binding ability (Figure 4A). As a control, a similarly purified protein, Ulp1, did not show such RNA-binding acitivity (data not shown). ATPase activity of Mb0671 was also seen, either alone, or in the presence of poly(U) or poly(C). Interestingly, unlike the RNA-dependent ATPase activity reported for cellular eIF4A proteins (Blum et al., 1992; Rogers et al., 2002), the ATPase activity of Mb0671 does not require the stimulation of RNA (Figure 4B). The presence of poly(U) or poly(C) enhanced its ATP hydrolysis activity only slightly, with poly(C) had a little stronger effect than poly(U). Another potential translation-related protein, SUI1 (Mb0678), did not exhibit ATPase activity (Figure 4B).

### Interacting proteins of Mb0671

**Figure 5:**
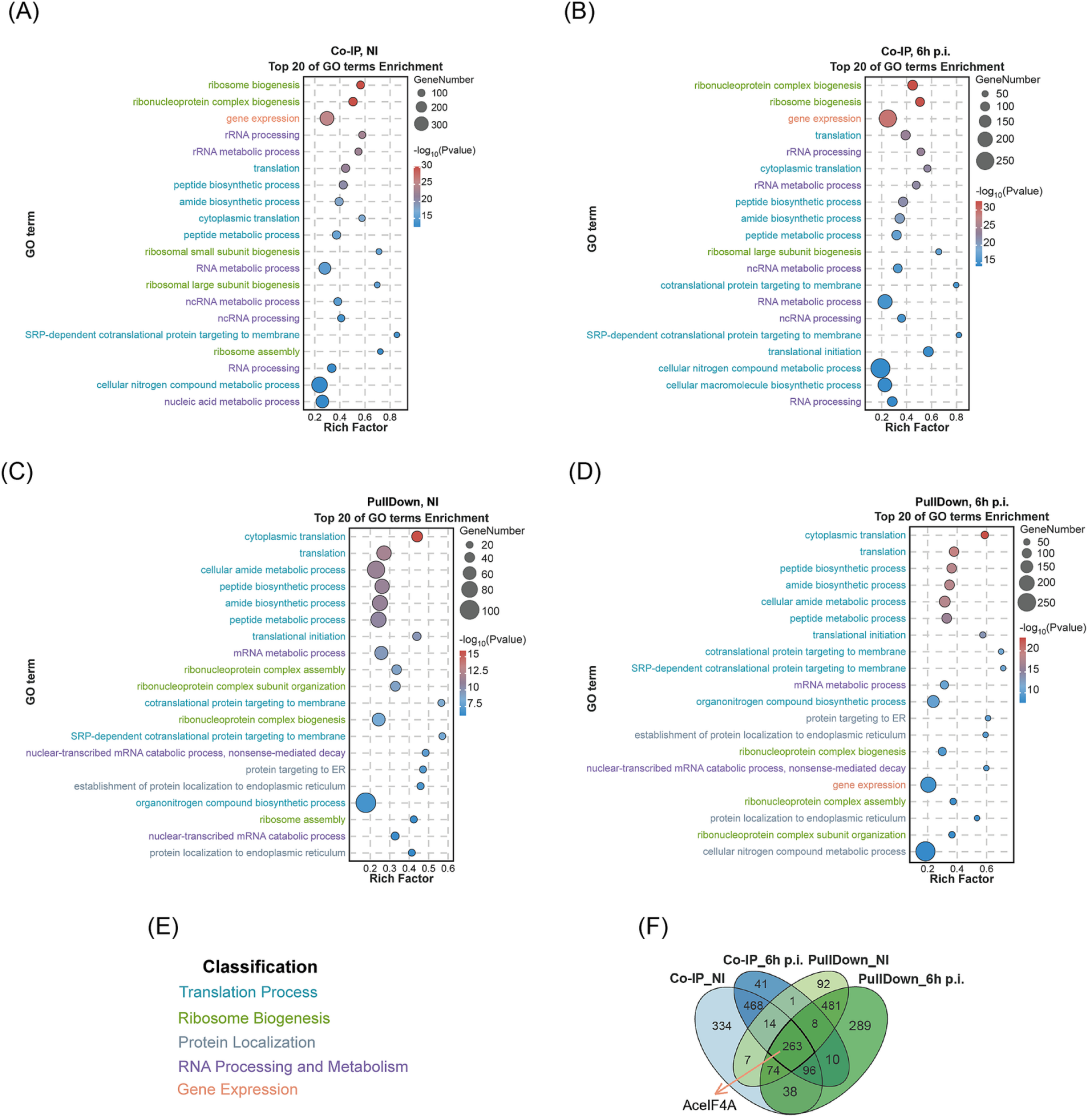
LC–MS/MS analysis of cellular proteins interacting with Mb0671. **(A–D)** Cell lysates were subjected to Co-IP experiments with Anti-FLAG immunomagnetic beads **(A,B)** or protein–protein pulldown experiments with the purified His-tagged Mb0671 protein **(C,D)**, followed by proteomic analysis using LC–MS/MS, and GO enrichment analysis. Top 20 GO terms (*p*-value < 0.01) were listed. **(A,C)** The cell lysate of uninfected *A. castellanii* cells overexpressing FLAG-tagged Mb0671. **(B,D)** The cell lysates of *A. castellanii* overexpressing FLAG-tagged Mb0671 infected by M. baoshan (MOI = 10) (6 h p.i.). **(E)** The coloring of GO terms. **(F)** Venn diagrams of these four sets of interactome data, with AceIF4A being present in all four datasets. NI: not infected.

Purified N-terminally His-tagged Mb0671 protein (His-Mb0671) was used to pulldown Mb0671-interacting proteins from cell lysates of *A. castellanii* cells either not infected (NI) or infected with M. baoshan (6 h p.i.). In another experiment, a cell line stably expressing FLAG-tagged Mb0671 (Ac_Mb0671) was established and used for *in vivo* coimmunoprecipitation (Co-IP) of proteins interacting with Mb0671 upon virus infection (6 h p.i.) or without infection (NI). The proteins from both pulldown and Co-IP experiments were subjected to liquid chromatography–tandem mass spectrometry (LC–MS/MS). The identified proteins from the four sets of interaction data (Supplementary Data Sheet 1) were analyzed for gene ontology (GO) enrichment. The top 20 enriched protein categories were primarily proteins involved in translation, ribosomal RNA processing, and gene expression (Figures 5A–E). In the Co-IP_NI, Co-IP_6h p.i., PullDown_NI, and PullDown_6h p.i. groups, there were 37, 37, 27, and 36 Mb0671-interacting proteins (Supplementary Data Sheet 2) significantly enriched in the GO term of translation initiation, respectively.

The Venn diagram illustrates the number of proteomics interacting proteins in the four groups and the intersections among them (Figure 5F), with the host translation initiation factor AceIF4A being detected in all four groups.

**Figure 6:**
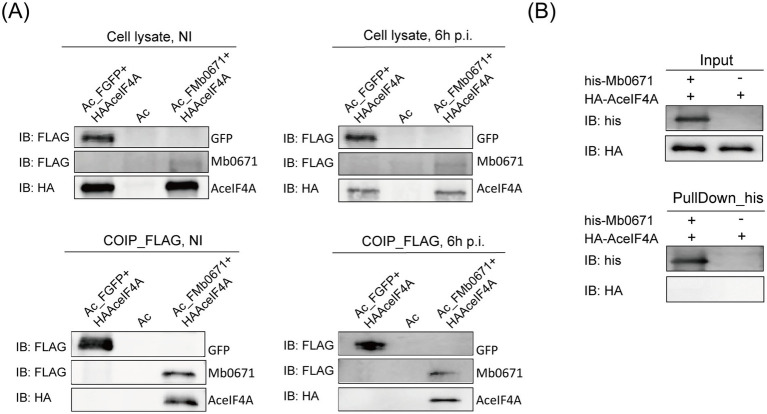
Immunoblot analysis of the interaction between AceIF4A and Mb0671. **(A)** Protein Co-IP assays in Ac_FMb0671 + HAAceIF4A and Ac_FGFP+HAAceIF4A with anti-FLAG MAb (1:2000) and anti-HA MAb (1:2000). **(B)** Protein–protein pulldown assays with anti-His MAb (1:2000) and anti-HA MAb (1:2000), using purified proteins of His-tagged Mb0671 and HA-tagged AceIF4A.

We constructed two *A. castellanii* cell lines co-expressing either FLAG-tagged Mb0671 and HA-tagged AceIF4A (Ac_FMb0671 + HAAceIF4A, also known as Ac_Mb0671 + AceIF4A), or FLAG-tagged GFP and HA-tagged AceIF4A (Ac_FGFP+HAAceIF4A). Co-IP/immunoblot experiments further confirmed the interaction between Mb0671 and AceIF4A (Figure 6A). However, further analysis of the interaction between Mb0671 and AceIF4A using purified HA-tagged AceIF4A (HA-AceIF4A) and His-tagged Mb0671 (His-Mb0671) with protein–protein pulldown assay showed that they did non interact with each other directly (Figure 6B). These results suggested the Mb0671 might associate with the translation complex.

### Mb0671 overexpressing altered protein profile

**Figure 7:**
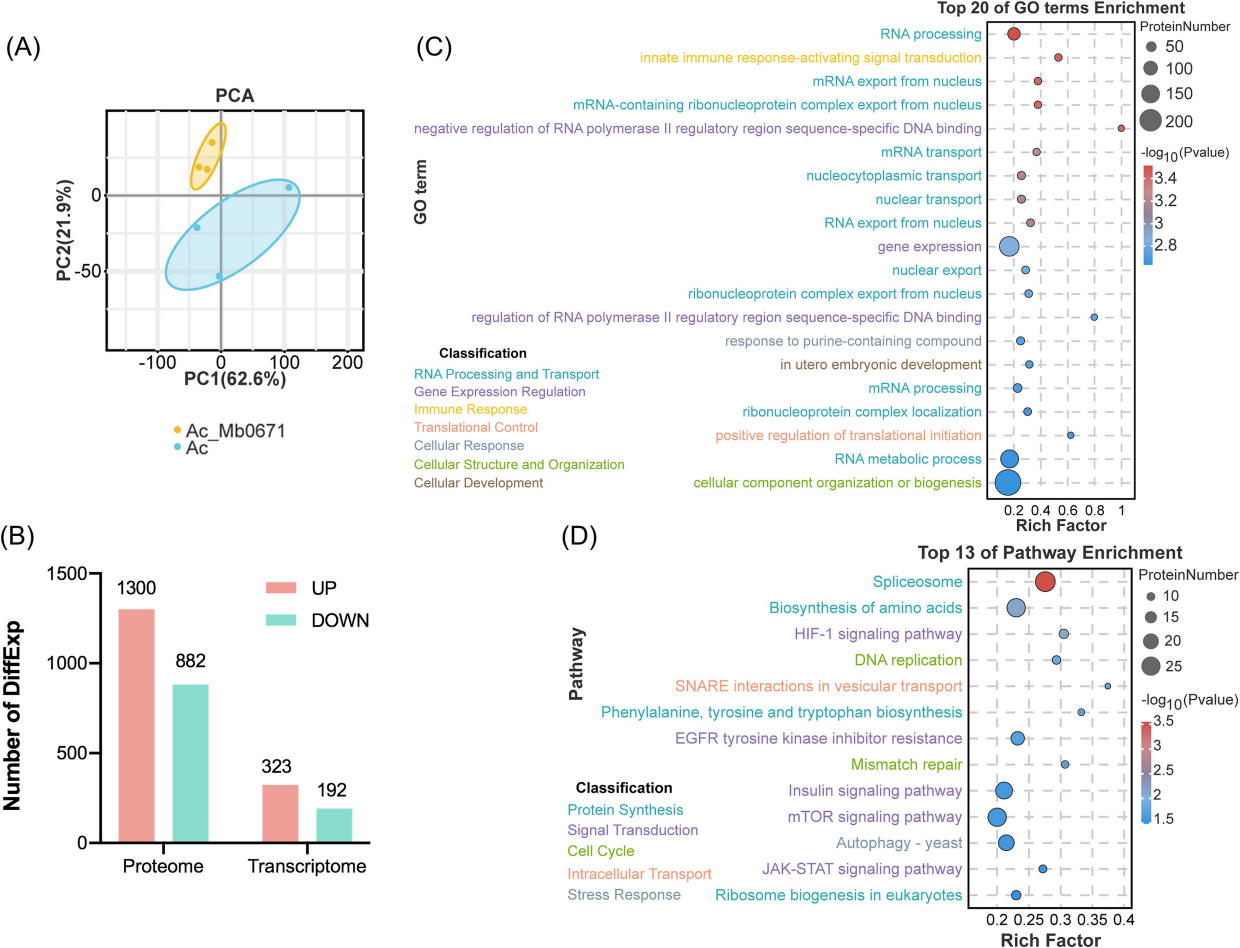
Analysis of differentially expressed proteins between Ac_Mb0671 and Ac. **(A)** Principal component analysis (PCA) of the proteomics samples. **(B)** Statistical analysis of differentially expressed (DiffExp) proteins and transcripts. **(C)** Graph of the Gene Ontology (GO) biological process enrichment analysis for DiffExp proteins upregulated in Ac_Mb0671 compared to Ac. The top 20 significantly enriched (*p*-value < 0.01) GO terms were shown. **(D)** Kyoto Encyclopedia of Genes and Genomes (KEGG) enrichment analysis of DiffExp proteins upregulated in the Ac_Mb0671 compared to Ac. The top 13 significantly enriched (*p*-value < 0.05) pathways were shown.

To further explore the impact of Mb0671 on host cells, cell lysates of *A. castellanii* overexpressing Mb0671 (Ac_Mb0671) and wild-type *A. castellanii* cell (Ac) were subjected to label-free quantitative proteomic analysis. Principal component analysis (PCA) of the proteomic data showed that samples of Ac_Mb0671 were separated well from that of Ac, indicating that the protein profiles in these two cell lines were significantly different (Figure 7A). Differential expression (DiffExp) analysis was performed on these two sets of proteomics data, and significantly up- or down-regulated proteins were selected according to |Log2(Fold Change)| > 1 and *p*-value < 0.05. Compared to Ac, Ac_Mb0671 has 1,300 proteins significantly upregulated and 882 proteins significantly downregulated (Figure 7B). GO enrichment analysis showed that, among upregulated proteins (Figure 7C), the top 20 significantly enriched GO terms (*p*-value < 0.01) were predominantly associated with mRNA processing and nuclear export, RNA metabolism and export, ribonucleoprotein complex assembly, etc. Other enriched GO terms included proteins in the regulation of gene expression, translation initiation, and organization or biogenesis of cellular components. KEGG analysis highlighted five signal transduction pathways that were enriched in Ac_Mb0671, including mTOR, HIF-1, EGFR tyrosine kinase inhibitor resistance, Insulin, and JAK–STAT signaling pathway (Figure 7D). Among these signaling pathways, the mTOR signaling pathway stood as the most central, as proteins enriched in the other pathway were essentially a subset of those found in the mTOR pathway (Supplementary Figure S2A; Supplementary Tables S3A–E).

Other important pathways with upregulated proteins enriched in Ac_Mb0671 included splicesome (Supplementary Figure S2B; Supplementary Table S3F), ribosome biogenesis (Supplementary Figure S2C; Supplementary Table S3G), biosynthesis of amino acids (Supplementary Tables S3H,I), autophagy (Supplementary Figure S2D; Supplementary Table S3J), soluble N-ethylmaleimidesensitive factor attachment protein receptor (SNARE) (Supplementary Figure S2E; Supplementary Table S3K), and DNA replication and mismatch repair signaling pathways (Supplementary Tables S3L,M).

In contrast, much less changes in mRNA level were seen in Mb0671-overexpressing cells. Transcriptome sequencing of Ac_Mb0671 and Ac cells revealed 9,319 and 9,022 cellular gene transcripts, respectively. Differential expression analysis identified 323 significantly upregulated and 192 downregulated genes (Figure 7B). Comparison of proteins significantly enriched in the top 16 signaling pathways showed that only genes, CypE and HSP73, involved in the spliceosome pathway, were significantly differentially expressed at the transcriptional level compared to wild-type, with Log2(fc) values of 1.4 and −1.6, respectively. The result suggested that Mb0671 acted mostly on the translation level.

### Mb0671 affected viral protein translation in infected cells

To investigate the effect of Mb0671 on the translation of M. baoshan proteins, infected cell lysate of Ac_Mb0671 and wild-type Ac cells (MOI = 10, 9 h p.i.) were used in label-free quantitative proteomic analysis. A total of 64 viral proteins were significantly upregulated (Log2(fc) > 1), and 19 proteins were significantly downregulated (Log2(fc) < −1) in Ac_Mb0671 compared to Ac (Supplementary Table S4).

### Mb0671 overexpression enhanced cell proliferation and accelerated virus replication

**Figure 8:**
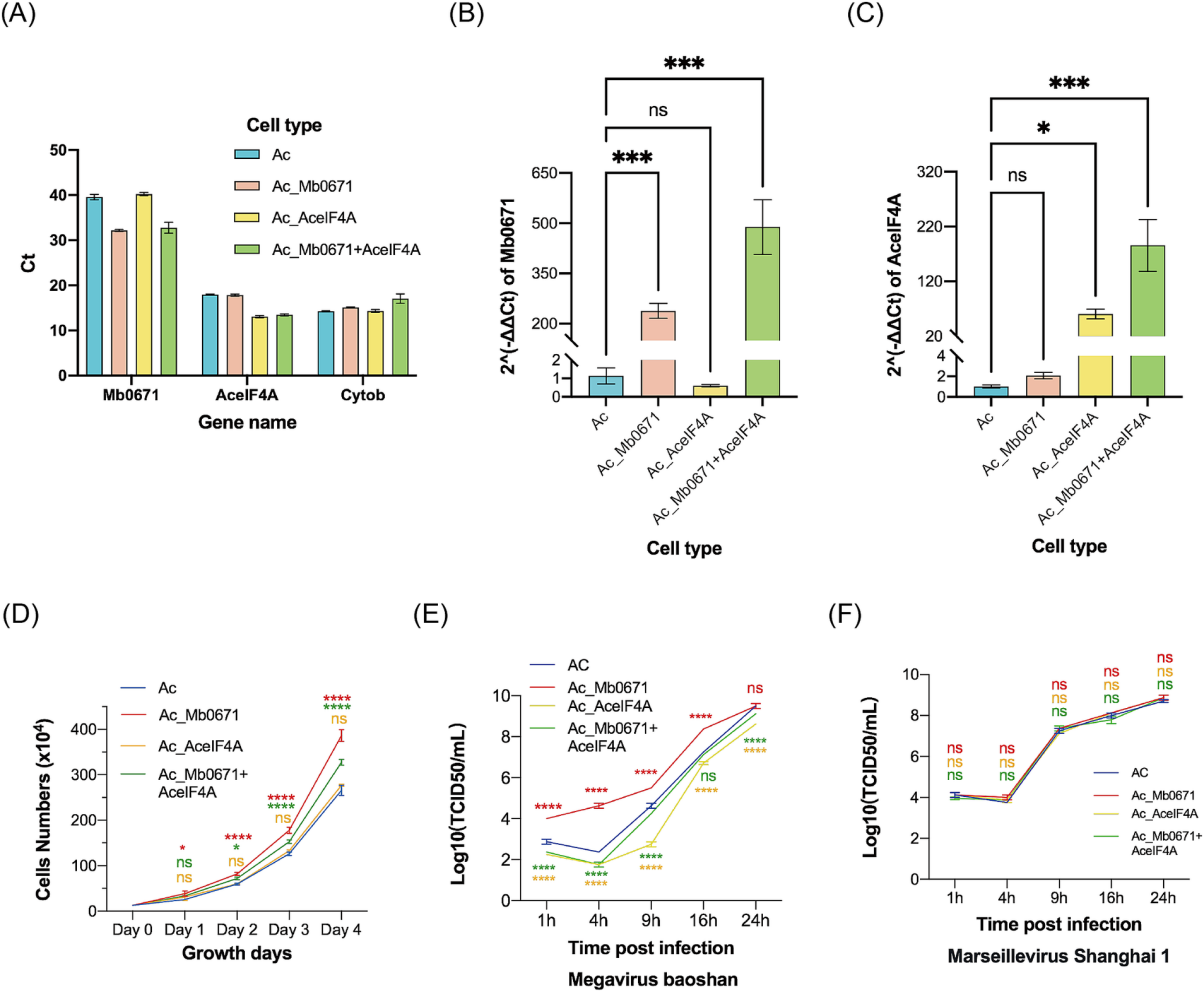
The impact of overexpression of Mb0671 on cell proliferation and viral replication. **(A–C)** RT-qPCR was used to detect the expression of Mb0671 and AceIF4A genes in Ac_Mb0671, Ac_AceIF4A, Ac_Mb0671 + eIF4A, and Ac cells, with the *A. castellanii* Cytob gene serving as the endogenous control. **(A)** The Ct values of Mb0671, AceIF4A, and Cytob genes in four cell lines. **(B)** The relative expression level of Mb0671 in four cell lines. **(C)** The relative expression level of AceIF4A in four cell lines. **(D)** Cell proliferation curves of four cells. **(E)** Viral replication curves of M. baoshan in four cell lines. **(F)** Viral replication curves of Marseillevirus Shanghai 1 in four cell lines. Student’s *t* test was performed to assess the significances of the difference between groups. *p ≤ 0.05; ***p ≤ 0.001; ****p < 0.0001; ns: *p* > 0.05.

Three cell lines of *A. castellanii* were established, either overexpressing Mb0671 (Ac_Mb0671), AceIF4A (Ac_AceIF4A), or both Mb0671 and AceIF4A (Ac_Mb0671 + AceIF4A). RT-qPCR was utilized to assess the expression of Mb0671 and AceIF4A genes in these three cell lines, together with the wild-type cell (Ac), using *A. castellanii* mitochondrial cytochrome b (Cytob) gene as an endogenous control. Mb0671 was confirmed to be expressed in both the Ac_Mb0671 and Ac_Mb0671 + AceIF4A cells (Figures 8A,B). AceIF4A was expressed at high levels in all four cell lines, with significantly higher expression in the two overexpressing lines, Ac_AceIF4A and Ac_Mb0671 + AceIF4A, compared to the non-overexpressing lines (Figures 8A,C).

Cell growth curves showed that Ac_Mb0671 grew significantly faster than Ac, while cells overexpressing Ac_AceIF4A grew at a similar rate as Ac. Ac_Mb0671 + AceIF4A grew at a rate between that of Ac and Ac_Mb0671 (Figure 8D).

Overexpression of Mb0671 significantly enhanced virus replication in the early stage, comparing with that in the wild-type cells (Figure 8E). In contrast, overexpression of AceIF4A seemed to slow down virus replication (Figure 8E). Co-overexpression of Mb0671 and AceIF4A also exhibited a slight inhibitory effect on viral infection (Figure 8E). Another amoeba giant virus from *Marseilleviridae*, Marseillevirus Shanghai 1, replicated similarly in all four cell lines (Figure 8F), suggesting that the activity of Mb0671 was specific for M. baoshan.

### Silencing Mb0671 reduced early virus production

**Figure 9:**
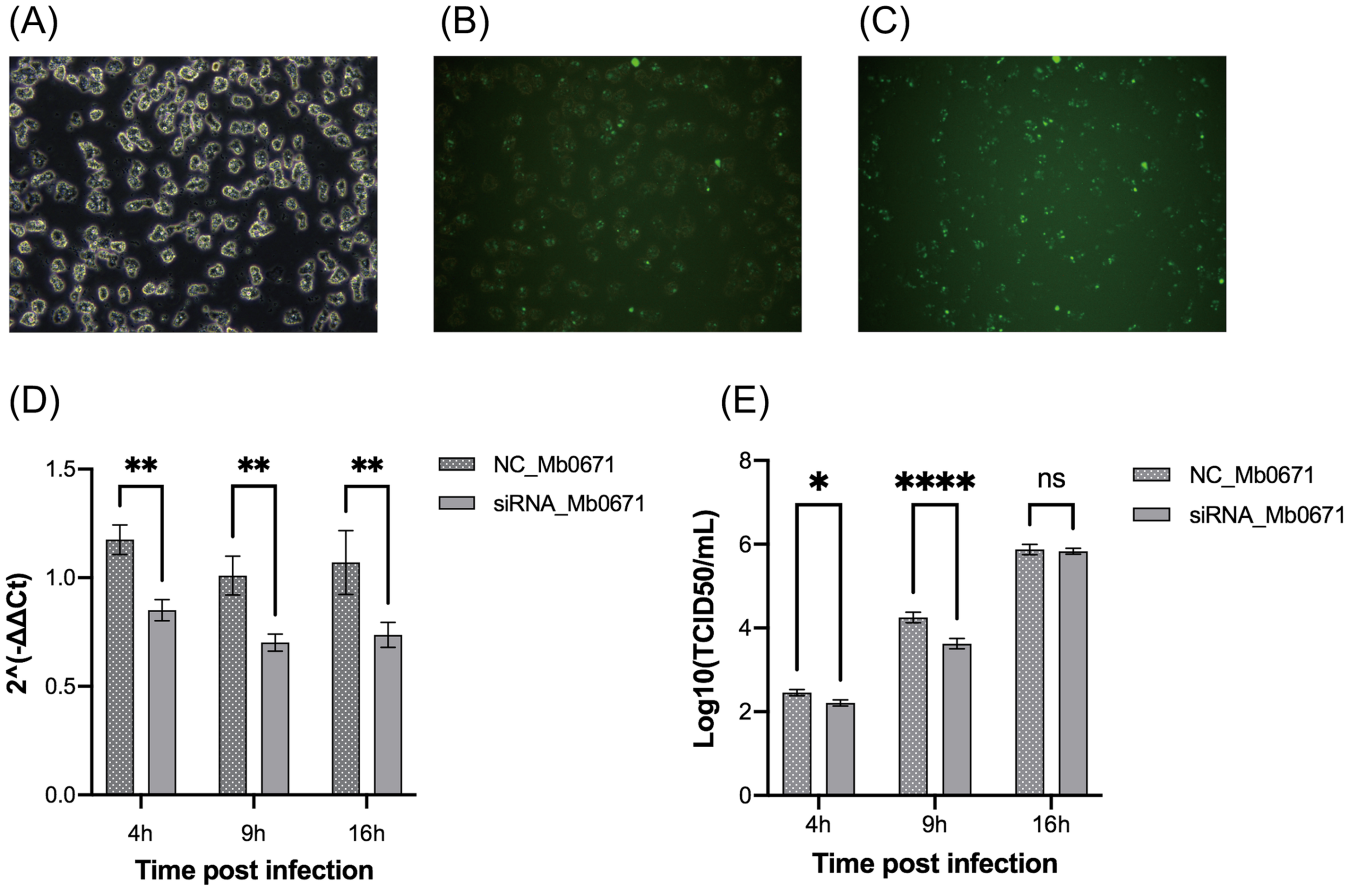
The impact of siRNA-mediated silencing of the Mb0671 gene on M. baoshan replication. **(A–C)** Efficiency of RNA transfection of in *A. castellanii* as revealed by green fluorescence in cells transfection with FAM-labeled short double-stranded RNA 4 h post transfection. **(A)** Bright field microcopy. **(B)** Merge of bright field and fluorescent microscopy. **(C)** Fluorescent microscopy. **(D)** Relative level of Mb0671 mRNA after transfection with Mb0671-specific siRNA or non-related control siRNA (NC) 4 h, 9 h, and 16 h p.i. **(E)** Virus titer of M. baoshan in cells transfected Mb0671-specific siRNA or NC siRNA 4 h, 9 h, and 16 h p.i. Student’s *t* test was performed to assess the significances of the difference between groups in **(D,E)**. *p ≤ 0.05; **p ≤ 0.01; ****p < 0.0001; ns: *p* > 0.05.

Transfection of *A. castellanii* cells with FAM-labeled short double-stranded RNA showed that the transfection efficiency was over 80% (Figures 9A–C). Four siRNAs targeting Mb0671 were designed, and the one with the best knockdown effect was selected for transfection into *A. castellanii* cells. The transfected cells were infected with M. baoshan 4 h post-transfection. Infected cells were collected 4 h, 9 h, and 16 h p.i., and Mb0671 transcription were determined with RT-qPCR. The results confirmed that the expression of Mb0671 was significantly reduced in transfected cells in comparison to un-transfected cells (Figure 9D). Virus titration showed that knockdown of Mb0671 significantly reduced virus production 4 and 9 h p.i., though yield of virus at the end infection (16 h p.i.) was not affected (Figure 9E).

Infected cells transfected with siRNA or NC siRNA were subjected to label-free quantitative proteomics analysis (MOI = 10, 4 h p.i.). The results showed that 154 viral proteins were detected in the NC group, while 153 viral proteins were detected in the siRNA knockdown group. Compared with the NC group, siRNA interference led to significant upregulation of 32 viral proteins and significant downregulation of 30 viral proteins (Supplementary Table S5).

## Discussion

There have been only limited researches on the translation-related proteins encoded by amoeba giant viruses. Bekliz et al. reported that silencing of the potential eIF4A encoded by APMV (R458) with RNAi resulted in decreased the level of viral DNA replication and the number of virus factories in infected cell, and deregulation of the expression of 32 virus proteins in a differential 2D electrophoresis. They suggested that the viral translation factor is functional during at least part of the infection cycle (Bekliz et al., 2018). Zinoviev et al. studied two groups of translational GTPases in giant viruses including Mimivirus and Marseillevirus with computational approach and *in vitro* reconstitution experiments, and showed that these proteins could be functional in the translation (Zinoviev et al., 2019). However, the role of these translation-related proteins in virus replication and their relationship with cellular translation mechanism remains unclear. In the current work, we focused on the functional role of the potential translation factor eIF4A (Mb0671) encoded by M. baoshan.

Bioinformatics analysis showed that Mb0671 belonged to the SrmB family of DEAD-box helicases. It possesses the basic structural domain of eIF4A but is evolutionarily closer to archaea, suggesting an early evolutionary origin. DEAD-box proteins are known to be required in almost all aspects of RNA metabolism, from their biogenesis to decay, in various organism from bacteria to mammals. In prokaryotes, SrmB family is involved in ribosome biogenesis (Iost and Jain, 2019; Rabuck-Gibbons et al., 2020; Trubetskoy et al., 2009), but due to the cytoplasmic localization of Mb0671 in *A. castellanii*, it is likely to serve a different function.

*In vitro* activity assays demonstrated that Mb0671 had non-RNA-dependent ATPase activity and RNA binding capacity similar to cellular eIF4A (Linder and Jankowsky, 2011; Ray et al., 1985; Rogers et al., 2002; Rogers et al., 2001; Rogers et al., 1999). However, no helicase activity has been detected to date, probably due to lack of key factors unknown to us yet.

Mb0671 was continuously expressed during virus infection, from 4 to 9 h p.i, with relatively high protein abundance compared to other virus-encoded translation-related proteins, but significantly lower than that of cellular eIF4A. Mb0671 was also found in virion, suggesting that Mb0671 might take part in the earliest viral translation, ensuring the initiation of viral replication. It is speculated that Mb0671 plays a crucial role throughout the viral replication cycle. This was consistent with the effect of Mb0671 in enhancing early virus production.

Immunofluorescent analysis confirmed the localization of Mb0671 in the cytoplasm, which was consistent with it predicted function in translation, though its co-localization with the cellular translation apparatus such as ribosome need to be further validated. However, analysis of the interactome of Mb0671 in both infected and uninfected cells did indicate that it interacted with many ribosomal proteins and translation factors. Parallel experiments in the laboratory with other proteins annotated to be translation related factors from a strain of Marseillevirus did not yield any significant enrichment of ribosomal proteins, indicating the results to be relieable. Co-IP and immunoblot confirmed its indirect interaction with cellular eIF4A. It is noted that during infection, the expression levels of host-encoded translation factors did not decrease significantly. Mb0671 might form complexes with host translation components and influence the translational preferences for mRNA, promoting the translation of viral proteins and selected cellular proteins in favor for virus replication. Additionally, Mb0671 was also found to interact with proteins related to gene expression, RNA processing and metabolism, suggesting its multiple functionalities.

Consistent with the results of interactome, overexpression of Mb0671 altered the cellular proteome. In uninfected *A. castellanii* cells, overexpression of Mb0671 led to significant upregulation of proteins related to cell growth, RNA metabolism, and protein synthesis, with significant enrichment in pathways such as mTOR, amino acid biosynthesis, ribosome biogenesis and spliceosome, which play crucial roles in protein synthesis (Chen et al., 2021; Clemens and Bommer, 1999; Dalla Venezia et al., 2019; He et al., 2021; Jacinto et al., 2004; Kim and Guan, 2019; Lamm et al., 2019; Leary and Huang, 2001; Ma and Blenis, 2009; Neufeld, 2010; Papasaikas and Valcárcel, 2016; Pelletier et al., 2018; Richardson et al., 2004; Wan et al., 2020). Significantly enriched pathways also include SNARE interactions in vesicular transport, essential for intracellular vesicular transport and protein trafficking (Fasshauer et al., 1998; Hackam et al., 1998; Holevinsky and Nelson, 1998; Jahn and Südhof, 1999; Mylvaganam et al., 2021; Söllner et al., 1993), and autophagy, crucial for maintaining cellular homeostasis and clearing misfolded proteins (Jacinto et al., 2004; May and Machesky, 2001).

How Mb0671 modulated the expression level of specific proteins is not yet clear. Since the transcriptome of the Mb0671-overexpressing cell altered to a much less level than the proteome, we presumed that Mb0671, as an RNA binding protein, might selectively bind to the special sequences or secondary structures of certain mRNAs to promote or inhibit their translation.

The overexpression of Mb0671 affected cell growth and created conditions favorable for virus replication, in accord with the change in cellular proteome. Cells overexpressing Mb0671 proliferated significantly faster than wild-type cells, and virus replication accelerated in Mb0671-overexpressing cell. These effects were not observed in cells overexpressing host AceIF4A. Consistently, the reduction of Mb0671 expression by siRNA delayed virus replication. These observations highlight the unique functionality of Mb0671.

The facts that Mb0671 was present in viral particles, and its expression increased with infection, and overexpression of it enhanced viral replication efficiency in the early stages of infection suggested that Mb0671 might have important role in early viral mRNA translation to ensure the normal initiation of viral replication. AceIF4A remained abundant during infection, indicating that Mb0671 did not replace the host translation machinery. Instead, viral replication primarily relied on the host’s translation mechanisms. The main cellular interaction partners of Mb0671 were found to be translation factors and ribosomal proteins (Supplementary Data Sheet 2). Overexpression of Mb0671 significantly altered the cellular protein composition, with upregulated proteins enriched in key pathways for protein synthesis and regulation, including amino acid biosynthesis, ribosome biogenesis, spliceosome, and mTOR signaling. Based on these observations, it is speculated that Mb0671 may manipulate host translation mechanisms to alter their mRNA preferences. Most eukaryotes’ eIF4G contains two eIF4A binding sites (Brito Querido et al., 2024). It is speculated that Mb0671 may compete for one of these sites and thus modulated the translation of certain mRNAs in infected cells, favoring viral replication. This hypothesis awaits validation with more detailed study. Experiments employing RNA pulldown or RNA immunoprecipitation might help to determine the specific RNA sequence Mb0671 bound to.

Overall, Mb0671 is believed to be capable of fine-tuning the host cell translation mechanisms, and selectively affected protein synthesis of virus and certain host proteins, thus to improve virus replication and its adaptability. The mechanism of Mb0671 to selectively up-regulate the translation of some proteins remained unclear. Mb0671 might bond to special structures in the 5′ end of some mRNAs, and enhance their translation initiation. The upregulation of mTOR and other essential pathways might further alter transcription of multiple genes.

Further studies are needed to elucidate the mechanism of Mb0671 to affect cellular proteome, especially its activity on RNA. Although Mb0671 has been shown to interact with translation-related proteins, direct evidence of its function in translation is limited. Future studies employing techniques such as ribosome profiling or polysome analysis could help validate its mechanism of action. Generating a Mb0671-knockout recombinant virus will be needed to confirm its role in virus replication and adaptability.

## Data Availability

The datasets presented in this study can be found in online repositories. The names of the repository/repositories and accession number(s) can be found in the article/Supplementary material.

## Footnotes

^1^ http://www.omicshare.com/tools

^2^ https://www.omicshare.com/

^3^ http://eggnog-mapper.embl.de/
