# Supplementary material for: Megavirus baoshanense Mb0671 modulates host translation and increases viral fitness
Source: Front Microbiol. 2025 Apr 28;16:1574090. doi: 10.3389/fmicb.2025.1574090 (PMC12066439; doi:10.3389/fmicb.2025.1574090)
Supplement: Supplementary file 1 [file Table_1.docx]

**Supplementary Table S1. Primers and RNAs**

**Table S1A. Primers used in PCR for constructing intracellular overexpression vectors.**

| **Primer Name** | **Sequence (5'-3')** |
| --- | --- |
| HA-AceIF4A-F | AAGATCTTCCATATGACTAGTTACCCATACGACGTCCCAGACTACGCTATGGATCAGGCTAAACTCAGCGAG |
| HA-AceIF4A-R | CTCCACCTCTAGACTTGTACAGTTAAGCGTAGTCTGGGACGTCGTATGGGTACAGGTAGGAGGCCACGTCG |
| FLAG-Mb0671-F | CACCACCAATCCACCCATATGGACTACAAGGACGATGACGACAAGATGAGTCAAGAACAAGAAAATATTTCAG |
| FLAG-Mb0671-R | TACCACGCGTGAATTTTACTTGTCGTCATCGTCCTTGTAGTCTAGTAAATTAGCCAAATCTCTAGGTAAAGG |
| FLAG-GFP-F | CACCACCAATCCACCCATATGGACTACAAGGACGATGACGACAAGATGGTGAGCAAGGGCGAG |
| FLAG-GFP-R | TACCACGCGTGAATTTTACTTGTCGTCATCGTCCTTGTAGTCCTTGTACAGCTCGTCCATGCC |
| GFP-Mb0671-F | TCGGCATGGACGAGCTGTACAAGATGAGTCAAGAACAAGAAAATATTTCAG |
| GFP-Mb0671-R | AAGCGGCCGCCTAGGGAATTCTTATAGTAAATTAGCCAAATCTCTAGGTAAAGG |

**Table S1B. Primers used in RT-qPCR.**

| **Primer Name** | **Sequence (5'-3')** |
| --- | --- |
| Mb0671-qF | GAGTGATTCTGCTTGTAGAACCTCC |
| Mb0671-qR | ACTACAAGAGATTGCACCGTTGC |
| AceIF4A-qF | ATCGGTATCCTCCAGCAGATCG |
| AceIF4A-qR | GAGGATGTGGGCCTTGATGTTC |
| Ac-Cytob-qF | ACCCCTCGTGAGCTTCTTTG |
| Ac-Cytob-qR | GAAAACCCACCCCATAGCCATATG |

**Table S1C. Primers used in PCR for constructing prokaryotic expression vector of protein.**

| **Primer Name** | **Sequence (5'-3')** |
| --- | --- |
| pSUMO-HA-AceIF4A-HA-F | CGCGGATCCTACCCATACGACGTCCCAGACTACGCTATGGATCAGGCTAAACTCAGCGAG |
| pSUMO-HA-AceIF4A-HA-R | CCCAAGCTTTTAAGCGTAGTCTGGGACGTCGTATGGGTACAGGTAGGAGGCCACGTCG |
| pET28a-Mb0671-F | GGAATTCCATATGATGAGTCAAGAACAAGAAAATATTTCAG |
| pET28a-Mb0671-R | CCCAAGCTTTAGTAAATTAGCCAAATCTCTAGGTAAAGG |
| pET28a-Mb0678-F | GGAATTCCATATGATGCTAGAAACTAATACTGGGATTTTTAATCC |
| pET28a-Mb0678-R | CCCAAGCTTCCATCCATGAATAATAATATCTTGAGATTTTACTATTG |

**Table S1D. RNA sequence for RNA crosslinking.**

| **RNA Name** | **Sequence (5'-3')** |
| --- | --- |
| FAM-U15 | FAM-UUUUUUUUUUUUUUU |

**Table S1E. siRNA sequence.**

| **RNA Name** | **Sequence (5'-3')** |
| --- | --- |
| Mb0671-355 | GGUGCAAUCUCUUGUAGUA |
| NC | UUCUCCGAACGUGUCACGUTT |
| FAM-NC | FAM-UUCUCCGAACGUGUCACGUTT |
