# Supplementary material for: Megavirus baoshanense Mb0671 modulates host translation and increases viral fitness
Source: Front Microbiol. 2025 Apr 28;16:1574090. doi: 10.3389/fmicb.2025.1574090 (PMC12066439; doi:10.3389/fmicb.2025.1574090)
Supplement: Supplementary file 2 [file Table_2.docx]

**Supplementary Table S2. Proteins in infected cells and virions**

**Table S2A. Proteins presented in infected cells at 4 h p.i.**

| **4 h p.i.** | | | |
| --- | --- | --- | --- |
| **Protein ID** | **Annotation** | **Abundance** | **Rank** |
| AZL89538.1 | VV D5-like helicase-primase | 1.32E+08 | 3 |
| AZL89973.1 | P-loop NTpase family | 1.13E+08 | 4 |
| AZL89448.1 | pyrophosphohydrolase | 1.11E+08 | 5 |
| AZL89519.1 | procollagen-lysine 2-oxoglutarate 5-dioxygenase | 7.63E+07 | 8 |
| AZL89345.1 | mRNA capping enzyme | 7.53E+07 | 9 |
| AZL89176.1 | glutamine synthetase | 7.46E+07 | 10 |
| AZL89437.1 | bifunctional polynucleotide phosphatase/kinase | 6.52E+07 | 13 |
| AZL89354.1 | heat shock 70 kDa protein-like protein | 5.33E+07 | 14 |
| AZL89424.1 | translation initiation factor 4a | 4.74E+07 | 16 |
| AZL89449.1 | DNA topoisomerase 2 | 4.46E+07 | 17 |
| AZL89667.1 | ariadne-like ring finger protein | 4.40E+07 | 18 |
| AZL89438.1 | DNA directed RNA polymerase subunit | 3.68E+07 | 21 |
| AZL89536.1 | DNA-directed RNA polymerase subunit 6 | 3.65E+07 | 22 |
| AZL89539.1 | serine/threonine protein kinase | 3.64E+07 | 23 |
| AZL89265.1 | ribonucleoside-diphosphate reductase small chain | 3.59E+07 | 24 |
| AZL89159.1 | flotillin domain protein | 3.01E+07 | 29 |
| AZL89282.1 | early transcription factor large subunit | 2.89E+07 | 31 |
| AZL89258.1 | protein phosphatase 2c | 2.76E+07 | 32 |
| AZL89468.1 | bifunctional dihydrofolate reductase-thymidylate synthase | 2.71E+07 | 33 |
| AZL89355.1 | heat shock 70 kDa protein-like protein | 2.67E+07 | 34 |
| AZL89155.1 | helicase | 2.43E+07 | 36 |
| AZL89998.1 | GMC-type oxidoreductase | 2.14E+07 | 39 |
| AZL89255.1 | DNA ligase | 2.04E+07 | 40 |
| AZL89218.1 | ubiquitin-conjugating enzyme E2 | 1.97E+07 | 42 |
| AZL89892.1 | fucosyltransferase | 1.93E+07 | 43 |
| AZL89426.1 | ubiquitin-conjugating enzyme E2 | 1.92E+07 | 44 |
| AZL89475.1 | DNA-directed RNA polymerase subunit 1 | 1.91E+07 | 45 |
| AZL89900.1 | EGF-like domain-containing protein | 1.83E+07 | 46 |
| AZL89295.1 | transcription factor S-II-related protein | 1.62E+07 | 48 |
| AZL89611.1 | KilA-N domain-containing protein | 1.53E+07 | 50 |
| AZL89444.1 | AAA family ATPase | 1.51E+07 | 51 |
| AZL89356.1 | HD domain-containing protein | 1.49E+07 | 52 |
| AZL89489.1 | DnaJ-like protein | 1.46E+07 | 53 |
| AZL89799.1 | metal-dependent phosphohydrolase HD domain protein | 1.38E+07 | 55 |
| AZL89366.1 | alpha/beta hydrolase | 1.31E+07 | 56 |
| AZL89392.1 | 5'-3'exonuclease | 1.16E+07 | 59 |
| AZL89391.1 | transcription factor | 1.13E+07 | 61 |
| UFX99826.1 | ubiquitin | 1.09E+07 | 64 |
| AZL89574.1 | tyrosyl-tRNA synthetase | 1.01E+07 | 66 |
| AZL89112.1 | GTP binding translation elongation/initiation factor | 1.00E+07 | 67 |
| AZL89157.1 | peptidase C19 subfamily protein | 9.59E+06 | 69 |
| AZL89389.1 | capsid protein | 8.09E+06 | 74 |
| UFX99824.1 | dTDP-d-glucose 4 6-dehydratase | 7.90E+06 | 75 |
| AZL89297.1 | poly(A) polymerase catalytic subunit | 7.81E+06 | 77 |
| AZL89185.1 | hydrolase | 7.58E+06 | 80 |
| AZL89293.1 | cullin,ankyrin repeat protein | 6.80E+06 | 82 |
| AZL89335.1 | glycosyltransferase | 6.42E+06 | 86 |
| AZL89443.1 | glutamine-dependent asparagine synthetase | 5.99E+06 | 88 |
| AZL89593.1 | purine phosphorylase | 5.80E+06 | 90 |
| AZL89122.1 | asparaginyl-tRNA synthetase | 5.49E+06 | 92 |
| AZL89192.1 | thioredoxin-like protein | 5.30E+06 | 94 |
| AZL89598.1 | purine phosphorylase,ankyrin repeat protein | 4.50E+06 | 97 |
| AZL89382.1 | nucleoside diphosphate kinase | 4.20E+06 | 99 |
| AZL89210.1 | DNA helicase | 3.66E+06 | 102 |
| AZL89151.1 | methyltransferase FkbM family protein | 3.42E+06 | 105 |
| AZL89415.1 | viral transcription factor 2 | 3.41E+06 | 106 |
| AZL89923.1 | serine/threonine-protein kinase | 2.88E+06 | 113 |
| AZL89504.1 | DNA-directed RNA polymerase subunit 2 | 2.65E+06 | 115 |
| AZL89266.1 | ribonucleoside-diphosphate reductase large subunit | 1.83E+06 | 122 |
| AZL89348.1 | endonuclease of the xpg family | 1.71E+06 | 123 |
| AZL89763.1 | serine/threonine-protein kinase | 1.65E+06 | 124 |
| AZL89636.1 | BTB/POZ domain-containing protein | 1.58E+06 | 126 |
| AZL89470.1 | replication factor C small subunit | 1.44E+06 | 128 |
| AZL89273.1 | DNA polymerase family X protein | 1.41E+06 | 129 |
| UFX99849.1 | intein-containing DNA-directed RNA polymerase subunit 2 | 1.19E+06 | 133 |
| AZL89409.1 | J domain-containing protein | 1.18E+06 | 134 |
| AZL89372.1 | core protein | 1.16E+06 | 135 |
| AZL89462.1 | DNA polymerase sliding clamp | 8.77E+05 | 139 |
| UFX99837.1 | replication factor C small subunit | 7.77E+05 | 140 |
| AZL89324.1 | ATP-dependent RNA helicase | 6.94E+05 | 141 |
| AZL89318.1 | glycosyltransferase | 6.73E+05 | 142 |
| AZL89490.1 | isoleucyl-tRNA synthetase,ankyrin repeat protein | 5.40E+05 | 144 |
| AZL89514.1 | DNA-directed RNA polymerase subunit 5 | 4.86E+05 | 145 |
| AZL89537.1 | DNA-dependent RNA polymerase subunit rpb9 | 3.38E+05 | 148 |
| AZL89906.1 | cysteinyl-tRNA synthetase | 3.31E+05 | 149 |
| AZL89236.1 | FtsJ-like methyltransferase | 2.29E+05 | 153 |
| AZL89642.1 | serine/threonine protein kinase receptor | 1.82E+05 | 154 |
| AZL89186.1 | ankyrin repeat protein | 7.07E+07 | 11 |
| AZL89752.1 | ankyrin repeat protein | 3.34E+07 | 27 |
| AZL89235.1 | ankyrin repeat protein | 1.19E+07 | 58 |
| AZL89658.1 | ankyrin repeat protein | 1.14E+07 | 60 |
| AZL89627.1 | ankyrin repeat protein | 1.09E+07 | 63 |
| AZL89576.1 | ankyrin repeat protein | 4.08E+06 | 100 |
| AZL89320.1 | ankyrin repeat protein | 3.65E+06 | 103 |
| AZL89601.1 | ankyrin repeat protein | 2.48E+06 | 116 |
| AZL89894.1 | ankyrin repeat protein | 1.27E+06 | 131 |
| AZL89835.1 | ankyrin repeat protein | 1.01E+06 | 137 |
| AZL89129.1 | ankyrin repeat protein | 2.54E+05 | 151 |
| AZL89946.1 | F-box and FNIP repeat-containing protein | 1.74E+07 | 47 |
| AZL89621.1 | F-box and FNIP repeat-containing protein | 6.57E+06 | 85 |
| AZL89101.1 | F-box and FNIP repeat-containing protein | 1.24E+06 | 132 |
| AZL89633.1 | F-box and FNIP repeat-containing protein | 6.71E+05 | 143 |
| AZL89138.1 | hypothetical protein Mb0368 | 2.51E+09 | 1 |
| AZL89124.1 | hypothetical protein Mb0354 | 3.51E+08 | 2 |
| AZL89179.1 | hypothetical protein Mb0414 | 1.06E+08 | 6 |
| AZL89433.1 | hypothetical protein Mb0680 | 8.72E+07 | 7 |
| AZL89543.1 | hypothetical protein Mb0799 | 6.59E+07 | 12 |
| AZL89406.1 | hypothetical protein Mb0653 | 4.87E+07 | 15 |
| AZL89478.1 | hypothetical protein Mb0728 | 3.81E+07 | 19 |
| AZL89234.1 | hypothetical protein Mb0472 | 3.74E+07 | 20 |
| AZL89493.1 | hypothetical protein Mb0743 | 3.48E+07 | 25 |
| AZL89359.1 | hypothetical protein Mb0603 | 3.47E+07 | 26 |
| AZL89476.1 | hypothetical protein Mb0726 | 3.22E+07 | 28 |
| AZL89924.1 | hypothetical protein Mb0260 | 2.89E+07 | 30 |
| AZL89206.1 | hypothetical protein Mb0442 | 2.63E+07 | 35 |
| UFX99712.1 | hypothetical protein Mb0021 | 2.27E+07 | 37 |
| AZL89174.1 | hypothetical protein Mb0407 | 2.19E+07 | 38 |
| AZL89661.1 | hypothetical protein Mb0073 | 2.02E+07 | 41 |
| AZL89143.1 | hypothetical protein Mb0374 | 1.61E+07 | 49 |
| AZL89294.1 | hypothetical protein Mb0536 | 1.38E+07 | 54 |
| AZL89364.1 | hypothetical protein Mb0608 | 1.27E+07 | 57 |
| AZL89652.1 | hypothetical protein Mb0084 | 1.11E+07 | 62 |
| AZL89686.1 | hypothetical protein Mb0042 | 1.03E+07 | 65 |
| AZL89429.1 | hypothetical protein Mb0676 | 1.00E+07 | 68 |
| UFX99868.1 | hypothetical protein Mb0836 | 8.72E+06 | 70 |
| UFX99908.1 | hypothetical protein Mb0990 | 8.56E+06 | 71 |
| AZL89196.1 | hypothetical protein Mb0432 | 8.54E+06 | 72 |
| AZL89524.1 | hypothetical protein Mb0779 | 8.25E+06 | 73 |
| AZL89280.1 | hypothetical protein Mb0520 | 7.88E+06 | 76 |
| AZL89691.1 | hypothetical protein Mb0037 | 7.73E+06 | 78 |
| AZL89771.1 | hypothetical protein Mb0927 | 7.68E+06 | 79 |
| AZL89244.1 | hypothetical protein Mb0482 | 7.35E+06 | 81 |
| UFX99828.1 | hypothetical protein Mb0624 | 6.75E+06 | 83 |
| AZL89607.1 | hypothetical protein Mb0139 | 6.67E+06 | 84 |
| AZL89685.1 | hypothetical protein Mb0044 | 6.42E+06 | 87 |
| AZL89250.1 | hypothetical protein Mb0488 | 5.94E+06 | 89 |
| AZL89619.1 | hypothetical protein Mb0125 | 5.66E+06 | 91 |
| AZL89588.1 | hypothetical protein Mb0163 | 5.42E+06 | 93 |
| AZL89123.1 | hypothetical protein Mb0353 | 5.01E+06 | 95 |
| AZL89580.1 | hypothetical protein Mb0171 | 4.89E+06 | 96 |
| AZL89606.1 | hypothetical protein Mb0141 | 4.43E+06 | 98 |
| AZL89972.1 | hypothetical protein Mb0835 | 3.81E+06 | 101 |
| AZL89477.1 | hypothetical protein Mb0727 | 3.63E+06 | 104 |
| AZL89776.1 | hypothetical protein Mb0932 | 3.40E+06 | 107 |
| AZL89842.1 | hypothetical protein Mb1007 | 3.37E+06 | 108 |
| AZL89774.1 | hypothetical protein Mb0930 | 3.32E+06 | 109 |
| AZL89134.1 | hypothetical protein Mb0364 | 3.06E+06 | 110 |
| AZL89341.1 | hypothetical protein Mb0584 | 2.91E+06 | 111 |
| AZL89183.1 | hypothetical protein Mb0419 | 2.90E+06 | 112 |
| AZL89464.1 | hypothetical protein Mb0713 | 2.78E+06 | 114 |
| AZL89644.1 | hypothetical protein Mb0092 | 2.18E+06 | 117 |
| AZL89784.1 | hypothetical protein Mb0941 | 2.17E+06 | 118 |
| AZL89479.1 | hypothetical protein Mb0729 | 2.17E+06 | 119 |
| AZL89457.1 | hypothetical protein Mb0705 | 2.16E+06 | 120 |
| AZL89540.1 | hypothetical protein Mb0796 | 1.91E+06 | 121 |
| AZL89954.1 | hypothetical protein Mb0854 | 1.58E+06 | 125 |
| AZL89180.1 | hypothetical protein Mb0415 | 1.44E+06 | 127 |
| AZL89141.1 | hypothetical protein Mb0371 | 1.32E+06 | 130 |
| AZL89965.1 | hypothetical protein Mb0842 | 1.10E+06 | 136 |
| AZL89459.1 | hypothetical protein Mb0708 | 9.51E+05 | 138 |
| AZL89458.1 | hypothetical protein Mb0706 | 3.96E+05 | 146 |
| AZL89769.1 | hypothetical protein Mb0924 | 3.44E+05 | 147 |
| AZL89984.1 | hypothetical protein Mb0822 | 3.12E+05 | 150 |
| AZL89330.1 | hypothetical protein Mb0573 | 2.39E+05 | 152 |

**Table S2B. Proteins presented in infected cells at 9 h p.i.**

| **9 h p.i.** | | | |
| --- | --- | --- | --- |
| **Protein ID** | **Annotation** | **Abundance** | **Rank** |
| AZL89354.1 | heat shock 70 kDa protein-like protein | 1.10E+09 | 3 |
| AZL89448.1 | pyrophosphohydrolase | 2.36E+08 | 9 |
| AZL89372.1 | core protein | 2.25E+08 | 10 |
| AZL89449.1 | DNA topoisomerase 2 | 1.83E+08 | 12 |
| AZL89973.1 | P-loop NTpase family | 1.62E+08 | 13 |
| AZL89319.1 | EXOV-like protein | 1.62E+08 | 14 |
| AZL89176.1 | glutamine synthetase | 1.45E+08 | 15 |
| AZL89159.1 | flotillin domain protein | 1.41E+08 | 16 |
| AZL89182.1 | HTH-type transcriptional regulator | 1.29E+08 | 17 |
| AZL89475.1 | DNA-directed RNA polymerase subunit 1 | 1.29E+08 | 18 |
| AZL89667.1 | ariadne-like ring finger protein | 1.29E+08 | 19 |
| AZL89538.1 | VV D5-like helicase-primase | 1.26E+08 | 20 |
| AZL89355.1 | heat shock 70 kDa protein-like protein | 1.18E+08 | 21 |
| AZL89409.1 | J domain-containing protein | 1.08E+08 | 24 |
| AZL89265.1 | ribonucleoside-diphosphate reductase small chain | 1.04E+08 | 25 |
| AZL89539.1 | serine/threonine protein kinase | 9.91E+07 | 27 |
| AZL89519.1 | procollagen-lysine 2-oxoglutarate 5-dioxygenase | 9.78E+07 | 28 |
| UFX99824.1 | dTDP-d-glucose 4 6-dehydratase | 9.48E+07 | 29 |
| AZL89424.1 | translation initiation factor 4a | 9.05E+07 | 30 |
| AZL89437.1 | bifunctional polynucleotide phosphatase/kinase | 8.54E+07 | 32 |
| AZL89345.1 | mRNA capping enzyme | 8.31E+07 | 33 |
| AZL89444.1 | AAA family ATPase | 6.88E+07 | 38 |
| AZL89317.1 | thioredoxin domain-containing protein | 6.66E+07 | 41 |
| AZL89258.1 | protein phosphatase 2c | 6.36E+07 | 43 |
| AZL89536.1 | DNA-directed RNA polymerase subunit 6 | 5.97E+07 | 45 |
| AZL89489.1 | DnaJ-like protein | 5.57E+07 | 46 |
| AZL89122.1 | asparaginyl-tRNA synthetase | 5.36E+07 | 47 |
| AZL89266.1 | ribonucleoside-diphosphate reductase large subunit | 5.18E+07 | 48 |
| AZL89438.1 | DNA directed RNA polymerase subunit | 4.98E+07 | 50 |
| AZL89155.1 | helicase | 4.79E+07 | 51 |
| AZL89900.1 | EGF-like domain-containing protein | 4.70E+07 | 52 |
| AZL89537.1 | DNA-dependent RNA polymerase subunit rpb9 | 4.63E+07 | 53 |
| AZL89218.1 | ubiquitin-conjugating enzyme E2 | 4.55E+07 | 55 |
| AZL89998.1 | GMC-type oxidoreductase | 3.98E+07 | 61 |
| AZL89462.1 | DNA polymerase sliding clamp | 3.91E+07 | 63 |
| AZL89192.1 | thioredoxin-like protein | 3.85E+07 | 64 |
| AZL89295.1 | transcription factor S-II-related protein | 3.68E+07 | 65 |
| AZL89366.1 | alpha/beta hydrolase | 3.58E+07 | 67 |
| UFX99826.1 | ubiquitin | 3.55E+07 | 68 |
| AZL89574.1 | tyrosyl-tRNA synthetase | 3.17E+07 | 73 |
| AZL89468.1 | bifunctional dihydrofolate reductase-thymidylate synthase | 3.15E+07 | 74 |
| AZL89356.1 | HD domain-containing protein | 3.11E+07 | 75 |
| AZL89288.1 | zinc finger MynD domain-containing protein | 2.83E+07 | 77 |
| AZL89157.1 | peptidase C19 subfamily protein | 2.63E+07 | 79 |
| AZL89392.1 | 5'-3'exonuclease | 2.56E+07 | 81 |
| AZL89892.1 | fucosyltransferase | 2.41E+07 | 84 |
| AZL89389.1 | capsid protein | 2.37E+07 | 85 |
| AZL89426.1 | ubiquitin-conjugating enzyme E2 | 2.27E+07 | 87 |
| AZL89443.1 | glutamine-dependent asparagine synthetase | 2.13E+07 | 89 |
| AZL89282.1 | early transcription factor large subunit | 2.10E+07 | 90 |
| AZL89467.1 | eukaryotic translation initiation factor 4e-like protein | 1.95E+07 | 93 |
| AZL89906.1 | cysteinyl-tRNA synthetase | 1.87E+07 | 95 |
| AZL89297.1 | poly(A) polymerase catalytic subunit | 1.86E+07 | 96 |
| AZL89490.1 | isoleucyl-tRNA synthetase,ankyrin repeat protein | 1.85E+07 | 97 |
| AZL89324.1 | ATP-dependent RNA helicase | 1.81E+07 | 98 |
| AZL89185.1 | hydrolase | 1.80E+07 | 100 |
| AZL89504.1 | DNA-directed RNA polymerase subunit 2 | 1.68E+07 | 103 |
| AZL89799.1 | metal-dependent phosphohydrolase HD domain protein | 1.63E+07 | 105 |
| AZL89382.1 | nucleoside diphosphate kinase | 1.59E+07 | 106 |
| UFX99843.1 | SWIB domain-containing protein | 1.58E+07 | 108 |
| AZL89151.1 | methyltransferase FkbM family protein | 1.50E+07 | 111 |
| AZL89255.1 | DNA ligase | 1.40E+07 | 113 |
| AZL89293.1 | cullin,ankyrin repeat protein | 1.39E+07 | 114 |
| AZL89237.1 | replication factor C small subunit | 1.38E+07 | 115 |
| AZL89514.1 | DNA-directed RNA polymerase subunit 5 | 1.31E+07 | 118 |
| AZL89335.1 | glycosyltransferase | 1.30E+07 | 119 |
| AZL89593.1 | purine phosphorylase | 1.22E+07 | 121 |
| AZL89611.1 | KilA-N domain-containing protein | 1.21E+07 | 122 |
| AZL89807.1 | methyltransferase type 11 | 1.18E+07 | 124 |
| AZL89415.1 | viral transcription factor 2 | 1.14E+07 | 126 |
| UFX99822.1 | regulator of chromosome condensation | 1.14E+07 | 128 |
| AZL89268.1 | formamidopyrimidine-DNA glycosylase | 1.07E+07 | 132 |
| UFX99827.1 | replication factor C small subunit | 9.53E+06 | 133 |
| AZL89112.1 | GTP binding translation elongation/initiation factor | 8.15E+06 | 140 |
| AZL89482.1 | ATP-dependent RNA helicase | 8.10E+06 | 141 |
| AZL89507.1 | DNA-directed RNA polymerase subunit 2 | 7.13E+06 | 146 |
| AZL89277.1 | B-family DNA polymerase | 7.12E+06 | 148 |
| AZL89986.1 | Cu/Zn superoxide dismutase | 6.86E+06 | 152 |
| AZL89551.1 | glycosyltransferase | 6.63E+06 | 154 |
| AZL89348.1 | endonuclease of the xpg family | 6.31E+06 | 156 |
| AZL89470.1 | replication factor C small subunit | 5.82E+06 | 161 |
| AZL89210.1 | DNA helicase | 5.73E+06 | 163 |
| AZL89598.1 | purine phosphorylase,ankyrin repeat protein | 5.58E+06 | 164 |
| AZL89496.1 | heat shock 70 kDa protein-like protein | 5.36E+06 | 165 |
| AZL89560.1 | JMJC domain protein | 5.19E+06 | 167 |
| AZL89623.1 | cholinesterase | 5.15E+06 | 168 |
| AZL89213.1 | 5'-3'exonuclease | 4.89E+06 | 170 |
| UFX99849.1 | intein-containing DNA-directed RNA polymerase subunit 2 | 4.72E+06 | 175 |
| AZL89391.1 | transcription factor | 4.37E+06 | 177 |
| AZL89760.1 | BTB/POZ domain-containing protein | 4.16E+06 | 180 |
| AZL89321.1 | UDP-N-acetylglucosamine 2-epimerase | 3.87E+06 | 183 |
| AZL89259.1 | mRNA capping enzyme | 3.54E+06 | 190 |
| AZL89993.1 | CfxQ-like protein | 3.52E+06 | 191 |
| AZL89923.1 | serine/threonine-protein kinase | 3.28E+06 | 193 |
| UFX99837.1 | replication factor C small subunit | 3.23E+06 | 194 |
| AZL89118.1 | glycylpeptide n-tetradecanoyltransferase | 3.09E+06 | 195 |
| AZL89120.1 | glucosamine-fructose-6-phosphate aminotransferase | 3.03E+06 | 196 |
| AZL89136.1 | glucose-methanol-choline oxidoreductase | 2.46E+06 | 205 |
| AZL89373.1 | factor C large subunit | 2.33E+06 | 208 |
| AZL89236.1 | FtsJ-like methyltransferase | 2.22E+06 | 210 |
| AZL89201.1 | ATP-dependent RNA helicase | 2.11E+06 | 211 |
| AZL89249.1 | endonuclease 4 | 1.62E+06 | 218 |
| AZL89903.1 | arginyl-tRNA synthetase | 1.37E+06 | 221 |
| AZL89636.1 | BTB/POZ domain-containing protein | 1.36E+06 | 222 |
| AZL89318.1 | glycosyltransferase | 1.30E+06 | 225 |
| AZL89164.1 | ATP-dependent helicase | 1.05E+06 | 229 |
| AZL89273.1 | DNA polymerase family X protein | 8.93E+05 | 231 |
| AZL89309.1 | phage-type endonuclease | 7.75E+05 | 232 |
| AZL89970.1 | ubiquitin-conjugating enzyme E2 | 2.98E+05 | 235 |
| AZL89642.1 | serine/threonine protein kinase receptor | 2.51E+04 | 236 |
| AZL89186.1 | ankyrin repeat protein | 2.20E+08 | 11 |
| AZL89235.1 | ankyrin repeat protein | 1.11E+08 | 23 |
| AZL89752.1 | ankyrin repeat protein | 4.14E+07 | 57 |
| AZL89658.1 | ankyrin repeat protein | 2.75E+07 | 78 |
| AZL89835.1 | ankyrin repeat protein | 2.59E+07 | 80 |
| AZL89894.1 | ankyrin repeat protein | 1.94E+07 | 94 |
| AZL89320.1 | ankyrin repeat protein | 1.65E+07 | 104 |
| AZL89627.1 | ankyrin repeat protein | 1.21E+07 | 123 |
| UFX99751.1 | ankyrin repeat protein | 4.81E+06 | 172 |
| AZL89601.1 | ankyrin repeat protein | 4.72E+06 | 174 |
| AZL89576.1 | ankyrin repeat protein | 4.28E+06 | 179 |
| AZL89747.1 | ankyrin repeat protein | 3.87E+06 | 184 |
| AZL89935.1 | ankyrin repeat protein | 3.86E+06 | 185 |
| AZL89913.1 | ankyrin repeat protein | 2.70E+06 | 200 |
| AZL89891.1 | ankyrin repeat protein | 2.29E+06 | 209 |
| AZL89247.1 | ankyrin repeat protein | 1.69E+06 | 215 |
| AZL89946.1 | F-box and FNIP repeat-containing protein | 1.73E+07 | 102 |
| AZL89621.1 | F-box and FNIP repeat-containing protein | 3.59E+06 | 188 |
| AZL89633.1 | F-box and FNIP repeat-containing protein | 5.17E+05 | 234 |
| AZL89138.1 | hypothetical protein Mb0368 | 2.17E+09 | 1 |
| AZL89124.1 | hypothetical protein Mb0354 | 1.19E+09 | 2 |
| AZL89543.1 | hypothetical protein Mb0799 | 6.75E+08 | 4 |
| AZL89478.1 | hypothetical protein Mb0728 | 4.46E+08 | 5 |
| AZL89179.1 | hypothetical protein Mb0414 | 3.59E+08 | 6 |
| AZL89406.1 | hypothetical protein Mb0653 | 3.39E+08 | 7 |
| AZL89433.1 | hypothetical protein Mb0680 | 2.82E+08 | 8 |
| AZL89479.1 | hypothetical protein Mb0729 | 1.16E+08 | 22 |
| AZL89398.1 | hypothetical protein Mb0645 | 1.04E+08 | 26 |
| AZL89215.1 | hypothetical protein Mb0451 | 9.01E+07 | 31 |
| UFX99828.1 | hypothetical protein Mb0624 | 7.93E+07 | 34 |
| UFX99813.1 | hypothetical protein Mb0471 | 7.42E+07 | 35 |
| AZL89180.1 | hypothetical protein Mb0415 | 7.28E+07 | 36 |
| AZL89661.1 | hypothetical protein Mb0073 | 7.20E+07 | 37 |
| AZL89965.1 | hypothetical protein Mb0842 | 6.77E+07 | 39 |
| AZL89748.1 | hypothetical protein Mb0903 | 6.69E+07 | 40 |
| AZL89361.1 | hypothetical protein Mb0605 | 6.53E+07 | 42 |
| AZL89954.1 | hypothetical protein Mb0854 | 6.19E+07 | 44 |
| AZL89234.1 | hypothetical protein Mb0472 | 4.98E+07 | 49 |
| AZL89347.1 | hypothetical protein Mb0590 | 4.62E+07 | 54 |
| AZL89206.1 | hypothetical protein Mb0442 | 4.41E+07 | 56 |
| AZL89331.1 | hypothetical protein Mb0574 | 4.14E+07 | 58 |
| AZL89294.1 | hypothetical protein Mb0536 | 4.12E+07 | 59 |
| AZL89457.1 | hypothetical protein Mb0705 | 4.03E+07 | 60 |
| AZL89143.1 | hypothetical protein Mb0374 | 3.97E+07 | 62 |
| AZL89196.1 | hypothetical protein Mb0432 | 3.62E+07 | 66 |
| AZL89499.1 | hypothetical protein Mb0751 | 3.53E+07 | 69 |
| AZL89359.1 | hypothetical protein Mb0603 | 3.38E+07 | 70 |
| AZL89280.1 | hypothetical protein Mb0520 | 3.32E+07 | 71 |
| AZL89476.1 | hypothetical protein Mb0726 | 3.28E+07 | 72 |
| AZL89183.1 | hypothetical protein Mb0419 | 3.10E+07 | 76 |
| AZL89776.1 | hypothetical protein Mb0932 | 2.55E+07 | 82 |
| AZL89495.1 | hypothetical protein Mb0745 | 2.43E+07 | 83 |
| AZL89540.1 | hypothetical protein Mb0796 | 2.34E+07 | 86 |
| AZL89364.1 | hypothetical protein Mb0608 | 2.25E+07 | 88 |
| AZL89628.1 | hypothetical protein Mb0112 | 2.07E+07 | 91 |
| AZL89464.1 | hypothetical protein Mb0713 | 1.96E+07 | 92 |
| AZL89442.1 | hypothetical protein Mb0689 | 1.81E+07 | 99 |
| AZL89498.1 | hypothetical protein Mb0750 | 1.80E+07 | 101 |
| AZL89691.1 | hypothetical protein Mb0037 | 1.59E+07 | 107 |
| AZL89619.1 | hypothetical protein Mb0125 | 1.56E+07 | 109 |
| AZL89174.1 | hypothetical protein Mb0407 | 1.52E+07 | 110 |
| AZL89477.1 | hypothetical protein Mb0727 | 1.40E+07 | 112 |
| AZL89580.1 | hypothetical protein Mb0171 | 1.35E+07 | 116 |
| AZL89771.1 | hypothetical protein Mb0927 | 1.34E+07 | 117 |
| AZL89458.1 | hypothetical protein Mb0706 | 1.22E+07 | 120 |
| UFX99848.1 | hypothetical protein Mb0753 | 1.17E+07 | 125 |
| AZL89952.1 | hypothetical protein Mb0856 | 1.14E+07 | 127 |
| AZL89972.1 | hypothetical protein Mb0835 | 1.11E+07 | 129 |
| AZL89244.1 | hypothetical protein Mb0482 | 1.09E+07 | 130 |
| UFX99908.1 | hypothetical protein Mb0990 | 1.07E+07 | 131 |
| AZL89379.1 | hypothetical protein Mb0623 | 9.07E+06 | 134 |
| AZL89397.1 | hypothetical protein Mb0644 | 9.03E+06 | 135 |
| AZL89341.1 | hypothetical protein Mb0584 | 8.88E+06 | 136 |
| AZL89812.1 | hypothetical protein Mb0975 | 8.39E+06 | 137 |
| AZL89432.1 | hypothetical protein Mb0679 | 8.29E+06 | 138 |
| AZL89123.1 | hypothetical protein Mb0353 | 8.28E+06 | 139 |
| AZL89429.1 | hypothetical protein Mb0676 | 8.01E+06 | 142 |
| AZL89459.1 | hypothetical protein Mb0708 | 7.67E+06 | 143 |
| AZL89607.1 | hypothetical protein Mb0139 | 7.34E+06 | 144 |
| AZL89250.1 | hypothetical protein Mb0488 | 7.28E+06 | 145 |
| AZL89107.1 | hypothetical protein Mb0338 | 7.13E+06 | 147 |
| UFX99846.1 | hypothetical protein Mb0747 | 7.08E+06 | 149 |
| UFX99785.1 | hypothetical protein Mb0301 | 7.02E+06 | 150 |
| AZL89769.1 | hypothetical protein Mb0924 | 6.99E+06 | 151 |
| AZL89882.1 | hypothetical protein Mb0309 | 6.72E+06 | 153 |
| UFX99756.1 | hypothetical protein Mb0186 | 6.51E+06 | 155 |
| UFX99868.1 | hypothetical protein Mb0836 | 6.30E+06 | 157 |
| AZL89685.1 | hypothetical protein Mb0044 | 5.95E+06 | 158 |
| AZL89217.1 | hypothetical protein Mb0453 | 5.84E+06 | 159 |
| AZL89276.1 | hypothetical protein Mb0516 | 5.82E+06 | 160 |
| AZL89774.1 | hypothetical protein Mb0930 | 5.75E+06 | 162 |
| AZL89917.1 | hypothetical protein Mb0267 | 5.31E+06 | 166 |
| UFX99753.1 | hypothetical protein Mb0179 | 4.91E+06 | 169 |
| AZL89784.1 | hypothetical protein Mb0941 | 4.86E+06 | 171 |
| AZL89134.1 | hypothetical protein Mb0364 | 4.78E+06 | 173 |
| AZL89925.1 | hypothetical protein Mb0259 | 4.51E+06 | 176 |
| UFX99712.1 | hypothetical protein Mb0021 | 4.31E+06 | 178 |
| AZL89493.1 | hypothetical protein Mb0743 | 4.12E+06 | 181 |
| AZL89686.1 | hypothetical protein Mb0042 | 4.05E+06 | 182 |
| AZL90012.1 | hypothetical protein Mb0226 | 3.60E+06 | 186 |
| AZL89606.1 | hypothetical protein Mb0141 | 3.60E+06 | 187 |
| AZL89524.1 | hypothetical protein Mb0779 | 3.58E+06 | 189 |
| AZL89555.1 | hypothetical protein Mb0199 | 3.33E+06 | 192 |
| AZL90000.1 | hypothetical protein Mb0214 | 3.00E+06 | 197 |
| AZL89793.1 | hypothetical protein Mb0952 | 3.00E+06 | 198 |
| AZL89141.1 | hypothetical protein Mb0371 | 2.98E+06 | 199 |
| AZL89842.1 | hypothetical protein Mb1007 | 2.68E+06 | 201 |
| AZL89116.1 | hypothetical protein Mb0346 | 2.60E+06 | 202 |
| AZL89308.1 | hypothetical protein Mb0550 | 2.60E+06 | 203 |
| AZL89353.1 | hypothetical protein Mb0597 | 2.54E+06 | 204 |
| AZL89187.1 | hypothetical protein Mb0423 | 2.46E+06 | 206 |
| AZL89652.1 | hypothetical protein Mb0084 | 2.43E+06 | 207 |
| AZL89307.1 | hypothetical protein Mb0549 | 2.10E+06 | 212 |
| AZL89456.1 | hypothetical protein Mb0703 | 1.93E+06 | 213 |
| AZL89330.1 | hypothetical protein Mb0573 | 1.73E+06 | 214 |
| AZL89109.1 | hypothetical protein Mb0340 | 1.67E+06 | 216 |
| AZL89352.1 | hypothetical protein Mb0596 | 1.65E+06 | 217 |
| UFX99779.1 | hypothetical protein Mb0255 | 1.62E+06 | 219 |
| AZL90005.1 | hypothetical protein Mb0219 | 1.48E+06 | 220 |
| UFX99839.1 | hypothetical protein Mb0704 | 1.33E+06 | 223 |
| AZL89541.1 | hypothetical protein Mb0797 | 1.31E+06 | 224 |
| AZL89562.1 | hypothetical protein Mb0191 | 1.28E+06 | 226 |
| AZL89644.1 | hypothetical protein Mb0092 | 1.18E+06 | 227 |
| AZL89588.1 | hypothetical protein Mb0163 | 1.05E+06 | 228 |
| AZL89984.1 | hypothetical protein Mb0822 | 9.62E+05 | 230 |
| AZL89363.1 | hypothetical protein Mb0607 | 5.30E+05 | 233 |

**Table S2C. Proteins present in virions.**

| **virion** | | | |
| --- | --- | --- | --- |
| **Protein ID** | **Annotation** | **Abundance** | **Rank** |
| UFX99822.1 | regulator of chromosome condensation | 4.40E+09 | 1 |
| AZL89372.1 | core protein | 2.15E+09 | 4 |
| AZL89389.1 | capsid protein | 1.93E+09 | 5 |
| AZL89317.1 | thioredoxin domain-containing protein | 4.72E+08 | 9 |
| AZL89136.1 | glucose-methanol-choline oxidoreductase | 1.51E+08 | 22 |
| AZL89485.1 | WD repeat-containing protein | 1.47E+08 | 23 |
| AZL89475.1 | DNA-directed RNA polymerase subunit 1 | 1.36E+08 | 26 |
| AZL89345.1 | mRNA capping enzyme | 1.04E+08 | 30 |
| AZL89306.1 | transcription termination factor | 9.14E+07 | 31 |
| UFX99909.1 | lipoprotein | 8.29E+07 | 33 |
| AZL89214.1 | alpha/beta hydrolase | 7.69E+07 | 36 |
| AZL89162.1 | N-acetyltransferase | 7.25E+07 | 37 |
| AZL89201.1 | ATP-dependent RNA helicase | 7.05E+07 | 38 |
| AZL89504.1 | DNA-directed RNA polymerase subunit 2 | 6.93E+07 | 39 |
| AZL89282.1 | early transcription factor large subunit | 6.65E+07 | 41 |
| AZL89340.1 | NTPase | 6.02E+07 | 42 |
| AZL89775.1 | virion-associated membrane protein | 5.62E+07 | 46 |
| AZL89213.1 | 5'-3'exonuclease | 5.47E+07 | 49 |
| AZL89195.1 | low complexity protein | 5.19E+07 | 52 |
| AZL89438.1 | DNA directed RNA polymerase subunit | 4.62E+07 | 55 |
| AZL89473.1 | DNA-directed RNA polymerase subunit 1 | 4.52E+07 | 59 |
| AZL89297.1 | poly(A) polymerase catalytic subunit | 4.46E+07 | 60 |
| AZL89507.1 | DNA-directed RNA polymerase subunit 2 | 4.00E+07 | 63 |
| AZL89221.1 | cyanovirin-N domain | 3.61E+07 | 66 |
| AZL89279.1 | myristoylated membrane protein | 3.13E+07 | 68 |
| AZL89454.1 | pan domain-containing protein | 2.60E+07 | 73 |
| UFX99865.1 | bifunctional metalloprotease/ubiquiton-like protein | 2.34E+07 | 75 |
| AZL89258.1 | protein phosphatase 2c | 2.33E+07 | 76 |
| UFX99849.1 | intein-containing DNA-directed RNA polymerase subunit 2 | 2.32E+07 | 77 |
| AZL89178.1 | ATP-dependent RNA helicase | 2.15E+07 | 79 |
| AZL89327.1 | ATP-dependent RNA helicase | 2.02E+07 | 81 |
| AZL90009.1 | DNA topoisomerase 1b | 2.00E+07 | 82 |
| AZL89273.1 | DNA polymerase family X protein | 1.89E+07 | 84 |
| AZL89362.1 | serine/threonine-protein kinase | 1.41E+07 | 87 |
| AZL89154.1 | prolyl 4-hydroxylase | 1.38E+07 | 88 |
| AZL89455.1 | pan domain-containing protein | 1.27E+07 | 89 |
| AZL89514.1 | DNA-directed RNA polymerase subunit 5 | 1.19E+07 | 91 |
| AZL89360.1 | phosphoesterase | 1.16E+07 | 92 |
| AZL89899.1 | histidine phosphatase | 1.13E+07 | 93 |
| AZL89338.1 | DNA-directed RNA polymerase subunit | 1.12E+07 | 94 |
| AZL89408.1 | ADP-ribosylglycohydrolase | 1.09E+07 | 95 |
| AZL89329.1 | FAD-linked sulfhydryl oxidase | 1.07E+07 | 96 |
| AZL89203.1 | ATP-dependent RNA helicase | 1.06E+07 | 97 |
| UFX99826.1 | ubiquitin | 1.00E+07 | 99 |
| AZL89255.1 | DNA ligase | 9.84E+06 | 101 |
| AZL89310.1 | thiol protease | 9.80E+06 | 102 |
| AZL89346.1 | FtsJ-like methyltransferase | 9.01E+06 | 105 |
| AZL89968.1 | p13-like protein | 7.92E+06 | 107 |
| UFX99861.1 | collagen-like protein | 7.47E+06 | 109 |
| UFX99851.1 | intein-containing DNA-directed RNA polymerase subunit 2 | 6.87E+06 | 111 |
| UFX99859.1 | glutaredoxin | 6.21E+06 | 113 |
| AZL89139.1 | lipocalin | 6.15E+06 | 114 |
| AZL89537.1 | DNA-dependent RNA polymerase subunit rpb9 | 5.88E+06 | 116 |
| AZL89253.1 | serine/threonine-protein kinase | 5.26E+06 | 118 |
| AZL89117.1 | tyrosine-protein phosphatase | 5.13E+06 | 119 |
| AZL89407.1 | thioredoxin domain-containing protein | 5.01E+06 | 120 |
| AZL89949.1 | UV-damage endonuclease | 5.00E+06 | 121 |
| AZL89554.1 | GMC-type oxidoreductase | 4.85E+06 | 122 |
| AZL89167.1 | helicase | 3.93E+06 | 127 |
| AZL89142.1 | structural ppiase-like protein | 3.92E+06 | 128 |
| AZL89348.1 | endonuclease of the xpg family | 3.61E+06 | 133 |
| UFX99860.1 | collagen-like protein | 3.43E+06 | 134 |
| AZL89271.1 | collagen-like protein | 3.40E+06 | 135 |
| AZL89192.1 | thioredoxin-like protein | 3.29E+06 | 137 |
| AZL89249.1 | endonuclease 4 | 3.12E+06 | 139 |
| AZL89831.1 | phosphatidyl ethanolamine-binding protein-like protein | 3.09E+06 | 140 |
| AZL89103.1 | lectin | 2.73E+06 | 144 |
| UFX99871.1 | serpin-like protein | 2.46E+06 | 145 |
| AZL90007.1 | endotype 6-aminohexanoat-oligomer hydrolase | 2.42E+06 | 146 |
| AZL89150.1 | FAD-linked sulfhydryl oxidase | 2.38E+06 | 147 |
| AZL89536.1 | DNA-directed RNA polymerase subunit 6 | 2.19E+06 | 148 |
| UFX99829.1 | short-chain type dehydrogenase/reductase | 1.94E+06 | 151 |
| UFX99888.1 | serine/threonine-protein kinase | 1.87E+06 | 152 |
| AZL89437.1 | bifunctional polynucleotide phosphatase/kinase | 1.81E+06 | 154 |
| UFX99858.1 | collagen-like protein | 1.77E+06 | 156 |
| AZL89929.1 | collagen-like protein | 1.57E+06 | 160 |
| AZL89956.1 | potassium channel protein | 1.36E+06 | 162 |
| UFX99823.1 | RNA polymerases subunit N | 1.13E+06 | 168 |
| AZL89281.1 | metallopeptidase WLM domain protein | 1.08E+06 | 173 |
| AZL89319.1 | EXOV-like protein | 8.73E+05 | 177 |
| AZL89497.1 | dual specificity phosphatase | 7.39E+05 | 180 |
| AZL89268.1 | formamidopyrimidine-DNA glycosylase | 7.18E+05 | 181 |
| AZL89982.1 | chemotaxis protein CheD | 6.55E+05 | 183 |
| AZL89409.1 | J domain-containing protein | 6.41E+05 | 184 |
| AZL89246.1 | ubiquitin carboxyl-terminal hydrolase | 6.17E+05 | 186 |
| AZL89516.1 | zinc protease | 5.08E+05 | 192 |
| AZL89251.1 | ribonuclease H protein | 5.05E+05 | 193 |
| AZL89336.1 | N-acetylglucosaminyl phosphatidyl inositol deacetylase | 4.96E+05 | 194 |
| AZL89998.1 | GMC-type oxidoreductase | 4.78E+05 | 195 |
| AZL89335.1 | glycosyltransferase | 4.35E+05 | 197 |
| AZL89900.1 | EGF-like domain-containing protein | 4.32E+05 | 198 |
| AZL89157.1 | peptidase C19 subfamily protein | 4.01E+05 | 199 |
| AZL89639.1 | KilA-N domain-containing protein | 3.80E+05 | 201 |
| AZL89617.1 | Fe2OG oxygenase family oxidoreductase | 3.65E+05 | 204 |
| UFX99922.1 | macrocin O-methyl transferase | 3.55E+05 | 205 |
| AZL89881.1 | deoxyribodipyrimidine photolyase-related protein | 3.11E+05 | 208 |
| AZL89354.1 | heat shock 70 kDa protein-like protein | 2.98E+05 | 209 |
| AZL89986.1 | Cu/Zn superoxide dismutase | 2.81E+05 | 211 |
| UFX99825.1 | glycosyltransferase | 2.49E+05 | 213 |
| UFX99866.1 | endonuclease VIII-like protein | 2.48E+05 | 214 |
| AZL89807.1 | methyltransferase type 11 | 2.01E+05 | 218 |
| AZL89403.1 | capsid protein | 1.71E+05 | 219 |
| AZL89424.1 | translation initiation factor 4a | 1.37E+05 | 223 |
| UFX99824.1 | dTDP-d-glucose 4 6-dehydratase | 1.14E+05 | 224 |
| AZL89247.1 | ankyrin repeat protein | 5.56E+07 | 47 |
| AZL89452.1 | ankyrin repeat protein | 4.56E+07 | 57 |
| AZL89332.1 | ankyrin repeat protein | 3.63E+06 | 131 |
| AZL89383.1 | ankyrin repeat protein | 4.58E+05 | 196 |
| AZL89270.1 | ankyrin repeat protein | 2.42E+05 | 216 |
| AZL89406.1 | hypothetical protein Mb0653 | 3.30E+09 | 2 |
| AZL89116.1 | hypothetical protein Mb0346 | 2.59E+09 | 3 |
| AZL89988.1 | hypothetical protein Mb0818 | 8.28E+08 | 6 |
| AZL89954.1 | hypothetical protein Mb0854 | 5.47E+08 | 7 |
| AZL89190.1 | hypothetical protein Mb0426 | 5.16E+08 | 8 |
| UFX99813.1 | hypothetical protein Mb0471 | 3.99E+08 | 10 |
| AZL89134.1 | hypothetical protein Mb0364 | 3.55E+08 | 11 |
| AZL89457.1 | hypothetical protein Mb0705 | 3.13E+08 | 12 |
| AZL89878.1 | hypothetical protein Mb0313 | 3.12E+08 | 13 |
| AZL89646.1 | hypothetical protein Mb0090 | 3.08E+08 | 14 |
| AZL89361.1 | hypothetical protein Mb0605 | 3.02E+08 | 15 |
| AZL89965.1 | hypothetical protein Mb0842 | 2.74E+08 | 16 |
| AZL89187.1 | hypothetical protein Mb0423 | 2.52E+08 | 17 |
| AZL89498.1 | hypothetical protein Mb0750 | 2.26E+08 | 18 |
| AZL89349.1 | hypothetical protein Mb0592 | 2.16E+08 | 19 |
| AZL89303.1 | hypothetical protein Mb0545 | 1.85E+08 | 20 |
| UFX99785.1 | hypothetical protein Mb0301 | 1.56E+08 | 21 |
| UFX99789.1 | hypothetical protein Mb0311 | 1.47E+08 | 24 |
| AZL89877.1 | hypothetical protein Mb0314 | 1.45E+08 | 25 |
| UFX99868.1 | hypothetical protein Mb0836 | 1.20E+08 | 27 |
| AZL89440.1 | hypothetical protein Mb0687 | 1.18E+08 | 28 |
| AZL89172.1 | hypothetical protein Mb0405 | 1.13E+08 | 29 |
| AZL89234.1 | hypothetical protein Mb0472 | 8.36E+07 | 32 |
| AZL89425.1 | hypothetical protein Mb0672 | 8.22E+07 | 34 |
| AZL89952.1 | hypothetical protein Mb0856 | 7.84E+07 | 35 |
| AZL89225.1 | hypothetical protein Mb0461 | 6.73E+07 | 40 |
| AZL89161.1 | hypothetical protein Mb0393 | 5.93E+07 | 43 |
| AZL89280.1 | hypothetical protein Mb0520 | 5.85E+07 | 44 |
| AZL89876.1 | hypothetical protein Mb0315 | 5.83E+07 | 45 |
| AZL89224.1 | hypothetical protein Mb0460 | 5.48E+07 | 48 |
| UFX99836.1 | hypothetical protein Mb0666 | 5.44E+07 | 50 |
| AZL89257.1 | hypothetical protein Mb0496 | 5.42E+07 | 51 |
| AZL89980.1 | hypothetical protein Mb0826 | 5.13E+07 | 53 |
| AZL89365.1 | hypothetical protein Mb0609 | 4.70E+07 | 54 |
| AZL89840.1 | hypothetical protein Mb1005 | 4.60E+07 | 56 |
| AZL89364.1 | hypothetical protein Mb0608 | 4.53E+07 | 58 |
| AZL89875.1 | hypothetical protein Mb0316 | 4.09E+07 | 61 |
| AZL89184.1 | hypothetical protein Mb0420 | 4.00E+07 | 62 |
| AZL89286.1 | hypothetical protein Mb0527 | 3.97E+07 | 64 |
| AZL89458.1 | hypothetical protein Mb0706 | 3.84E+07 | 65 |
| AZL89453.1 | hypothetical protein Mb0700 | 3.45E+07 | 67 |
| AZL89208.1 | hypothetical protein Mb0444 | 3.12E+07 | 69 |
| UFX99803.1 | hypothetical protein Mb0388 | 2.90E+07 | 70 |
| UFX99817.1 | hypothetical protein Mb0501 | 2.87E+07 | 71 |
| AZL89461.1 | hypothetical protein Mb0710 | 2.84E+07 | 72 |
| UFX99847.1 | hypothetical protein Mb0749 | 2.53E+07 | 74 |
| UFX99842.1 | hypothetical protein Mb0722 | 2.20E+07 | 78 |
| AZL89966.1 | hypothetical protein Mb0841 | 2.12E+07 | 80 |
| AZL89352.1 | hypothetical protein Mb0596 | 1.99E+07 | 83 |
| AZL89430.1 | hypothetical protein Mb0677 | 1.78E+07 | 85 |
| AZL89339.1 | hypothetical protein Mb0582 | 1.51E+07 | 86 |
| AZL89351.1 | hypothetical protein Mb0595 | 1.25E+07 | 90 |
| UFX99839.1 | hypothetical protein Mb0704 | 1.05E+07 | 98 |
| AZL89378.1 | hypothetical protein Mb0622 | 1.00E+07 | 100 |
| AZL89527.1 | hypothetical protein Mb0782 | 9.77E+06 | 103 |
| AZL89197.1 | hypothetical protein Mb0433 | 9.18E+06 | 104 |
| AZL89353.1 | hypothetical protein Mb0597 | 8.60E+06 | 106 |
| AZL89315.1 | hypothetical protein Mb0558 | 7.74E+06 | 108 |
| AZL89753.1 | hypothetical protein Mb0908 | 7.04E+06 | 110 |
| AZL89379.1 | hypothetical protein Mb0623 | 6.81E+06 | 112 |
| AZL89419.2 | hypothetical protein Mb0392 | 6.07E+06 | 115 |
| AZL89376.1 | hypothetical protein Mb0620 | 5.42E+06 | 117 |
| AZL89307.1 | hypothetical protein Mb0549 | 4.79E+06 | 123 |
| AZL89135.1 | hypothetical protein Mb0365 | 4.40E+06 | 124 |
| AZL89188.1 | hypothetical protein Mb0424 | 4.20E+06 | 125 |
| UFX99816.1 | hypothetical protein Mb0500 | 4.01E+06 | 126 |
| AZL89513.1 | hypothetical protein Mb0768 | 3.82E+06 | 129 |
| AZL89423.1 | hypothetical protein Mb0670 | 3.65E+06 | 130 |
| AZL89901.1 | hypothetical protein Mb0287 | 3.62E+06 | 132 |
| AZL89471.1 | hypothetical protein Mb0720 | 3.29E+06 | 136 |
| AZL89481.2 | hypothetical protein Mb0456 | 3.20E+06 | 138 |
| AZL89987.1 | hypothetical protein Mb0819 | 3.05E+06 | 141 |
| AZL89312.1 | hypothetical protein Mb0554 | 2.99E+06 | 142 |
| AZL89284.1 | hypothetical protein Mb0525 | 2.88E+06 | 143 |
| AZL89975.1 | hypothetical protein Mb0831 | 2.06E+06 | 149 |
| AZL89736.1 | hypothetical protein Mb0887 | 2.03E+06 | 150 |
| AZL89107.1 | hypothetical protein Mb0338 | 1.83E+06 | 153 |
| AZL89466.1 | hypothetical protein Mb0715 | 1.78E+06 | 155 |
| UFX99701.1 | hypothetical protein Mb0009 | 1.73E+06 | 157 |
| AZL89436.1 | hypothetical protein Mb0683 | 1.67E+06 | 158 |
| AZL89333.1 | hypothetical protein Mb0576 | 1.64E+06 | 159 |
| UFX99819.1 | hypothetical protein Mb0524 | 1.36E+06 | 161 |
| AZL89802.1 | hypothetical protein Mb0963 | 1.35E+06 | 163 |
| AZL89413.1 | hypothetical protein Mb0660 | 1.30E+06 | 164 |
| AZL89163.1 | hypothetical protein Mb0396 | 1.27E+06 | 165 |
| AZL89417.1 | hypothetical protein Mb0664 | 1.20E+06 | 166 |
| AZL89124.1 | hypothetical protein Mb0354 | 1.19E+06 | 167 |
| AZL89879.1 | hypothetical protein Mb0312 | 1.11E+06 | 169 |
| AZL89614.1 | hypothetical protein Mb0132 | 1.11E+06 | 170 |
| AZL89798.1 | hypothetical protein Mb0959 | 1.10E+06 | 171 |
| AZL89137.1 | hypothetical protein Mb0367 | 1.10E+06 | 172 |
| AZL89499.1 | hypothetical protein Mb0751 | 1.00E+06 | 174 |
| AZL89292.1 | hypothetical protein Mb0534 | 9.62E+05 | 175 |
| AZL89278.1 | hypothetical protein Mb0518 | 9.37E+05 | 176 |
| AZL89371.1 | hypothetical protein Mb0615 | 7.82E+05 | 178 |
| AZL89375.1 | hypothetical protein Mb0619 | 7.43E+05 | 179 |
| AZL89263.1 | hypothetical protein Mb0503 | 6.69E+05 | 182 |
| AZL89397.1 | hypothetical protein Mb0644 | 6.34E+05 | 185 |
| AZL89534.1 | hypothetical protein Mb0789 | 6.05E+05 | 187 |
| AZL89479.1 | hypothetical protein Mb0729 | 5.78E+05 | 188 |
| AZL89737.1 | hypothetical protein Mb0888 | 5.54E+05 | 189 |
| UFX99802.1 | hypothetical protein Mb0373 | 5.14E+05 | 190 |
| AZL89953.1 | hypothetical protein Mb0855 | 5.14E+05 | 191 |
| AZL89950.1 | hypothetical protein Mb0858 | 3.86E+05 | 200 |
| AZL89874.1 | hypothetical protein Mb0317 | 3.70E+05 | 202 |
| AZL89863.1 | hypothetical protein Mb1034 | 3.68E+05 | 203 |
| AZL89478.1 | hypothetical protein Mb0728 | 3.22E+05 | 206 |
| AZL89812.1 | hypothetical protein Mb0975 | 3.17E+05 | 207 |
| AZL89529.1 | hypothetical protein Mb0784 | 2.85E+05 | 210 |
| AZL89793.1 | hypothetical protein Mb0952 | 2.72E+05 | 212 |
| AZL89580.1 | hypothetical protein Mb0171 | 2.45E+05 | 215 |
| AZL89202.1 | hypothetical protein Mb0438 | 2.11E+05 | 217 |
| AZL89250.1 | hypothetical protein Mb0488 | 1.68E+05 | 220 |
| AZL89957.1 | hypothetical protein Mb0851 | 1.61E+05 | 221 |
| AZL89180.1 | hypothetical protein Mb0415 | 1.58E+05 | 222 |
| AZL89359.1 | hypothetical protein Mb0603 | 1.12E+05 | 225 |
| AZL89484.1 | hypothetical protein Mb0734 | 9.35E+04 | 226 |

Table S2D. Proteins presented in virions and infected cells at 4 h and 9 h p.i.

| **Protein ID** | **Annotation** | **virion** | | **4 h p.i.** | | **9 h p.i.** | |
| --- | --- | --- | --- | --- | --- | --- | --- |
|  |  | **Abundance** | **Rank** | **Abundance** | **Rank** | **Abundance** | **Rank** |
| AZL89372.1 | core protein | 2.15E+09 | 4 | 1.16E+06 | 135 | 2.25E+08 | 10 |
| AZL89389.1 | capsid protein | 1.93E+09 | 5 | 8.09E+06 | 74 | 2.37E+07 | 85 |
| AZL89475.1 | DNA-directed RNA polymerase subunit 1 | 1.36E+08 | 26 | 1.91E+07 | 45 | 1.29E+08 | 18 |
| AZL89345.1 | mRNA capping enzyme | 1.04E+08 | 30 | 7.53E+07 | 9 | 8.31E+07 | 33 |
| AZL89504.1 | DNA-directed RNA polymerase subunit 2 | 6.93E+07 | 39 | 2.65E+06 | 115 | 1.68E+07 | 103 |
| AZL89282.1 | early transcription factor large subunit | 6.65E+07 | 41 | 2.89E+07 | 31 | 2.10E+07 | 90 |
| AZL89438.1 | DNA directed RNA polymerase subunit | 4.62E+07 | 55 | 3.68E+07 | 21 | 4.98E+07 | 50 |
| AZL89297.1 | poly(A) polymerase catalytic subunit | 4.46E+07 | 60 | 7.81E+06 | 77 | 1.86E+07 | 96 |
| AZL89258.1 | protein phosphatase 2c | 2.33E+07 | 76 | 2.76E+07 | 32 | 6.36E+07 | 43 |
| UFX99849.1 | intein-containing DNA-directed RNA polymerase subunit 2 | 2.32E+07 | 77 | 1.19E+06 | 133 | 4.72E+06 | 175 |
| AZL89273.1 | DNA polymerase family X protein | 1.89E+07 | 84 | 1.41E+06 | 129 | 8.93E+05 | 231 |
| AZL89514.1 | DNA-directed RNA polymerase subunit 5 | 1.19E+07 | 91 | 4.86E+05 | 145 | 1.31E+07 | 118 |
| UFX99826.1 | ubiquitin | 1.00E+07 | 99 | 1.09E+07 | 64 | 3.55E+07 | 68 |
| AZL89255.1 | DNA ligase | 9.84E+06 | 101 | 2.04E+07 | 40 | 1.40E+07 | 113 |
| AZL89537.1 | DNA-dependent RNA polymerase subunit rpb9 | 5.88E+06 | 116 | 3.38E+05 | 148 | 4.63E+07 | 53 |
| AZL89348.1 | endonuclease of the xpg family | 3.61E+06 | 133 | 1.71E+06 | 123 | 6.31E+06 | 156 |
| AZL89192.1 | thioredoxin-like protein | 3.29E+06 | 137 | 5.30E+06 | 94 | 3.85E+07 | 64 |
| AZL89536.1 | DNA-directed RNA polymerase subunit 6 | 2.19E+06 | 148 | 3.65E+07 | 22 | 5.97E+07 | 45 |
| AZL89437.1 | bifunctional polynucleotide phosphatase/kinase | 1.81E+06 | 154 | 6.52E+07 | 13 | 8.54E+07 | 32 |
| AZL89409.1 | J domain-containing protein | 6.41E+05 | 184 | 1.18E+06 | 134 | 1.08E+08 | 24 |
| AZL89998.1 | GMC-type oxidoreductase | 4.78E+05 | 195 | 2.14E+07 | 39 | 3.98E+07 | 61 |
| AZL89335.1 | glycosyltransferase | 4.35E+05 | 197 | 6.42E+06 | 86 | 1.30E+07 | 119 |
| AZL89900.1 | EGF-like domain-containing protein | 4.32E+05 | 198 | 1.83E+07 | 46 | 4.70E+07 | 52 |
| AZL89157.1 | peptidase C19 subfamily protein | 4.01E+05 | 199 | 9.59E+06 | 69 | 2.63E+07 | 79 |
| AZL89354.1 | heat shock 70 kDa protein-like protein | 2.98E+05 | 209 | 5.33E+07 | 14 | 1.10E+09 | 3 |
| AZL89424.1 | translation initiation factor 4a* | 1.37E+05 | 223 | 4.74E+07 | 16 | 9.05E+07 | 30 |
| UFX99824.1 | dTDP-d-glucose 4 6-dehydratase | 1.14E+05 | 224 | 7.90E+06 | 75 | 9.48E+07 | 29 |
| AZL89406.1 | hypothetical protein Mb0653 | 3.30E+09 | 2 | 4.87E+07 | 15 | 3.39E+08 | 7 |
| AZL89954.1 | hypothetical protein Mb0854 | 5.47E+08 | 7 | 1.58E+06 | 125 | 6.19E+07 | 44 |
| AZL89134.1 | hypothetical protein Mb0364 | 3.55E+08 | 11 | 3.06E+06 | 110 | 4.78E+06 | 173 |
| AZL89457.1 | hypothetical protein Mb0705 | 3.13E+08 | 12 | 2.16E+06 | 120 | 4.03E+07 | 60 |
| AZL89965.1 | hypothetical protein Mb0842 | 2.74E+08 | 16 | 1.10E+06 | 136 | 6.77E+07 | 39 |
| UFX99868.1 | hypothetical protein Mb0836 | 1.20E+08 | 27 | 8.72E+06 | 70 | 6.30E+06 | 157 |
| AZL89234.1 | hypothetical protein Mb0472 | 8.36E+07 | 32 | 3.74E+07 | 20 | 4.98E+07 | 49 |
| AZL89280.1 | hypothetical protein Mb0520 | 5.85E+07 | 44 | 7.88E+06 | 76 | 3.32E+07 | 71 |
| AZL89364.1 | hypothetical protein Mb0608 | 4.53E+07 | 58 | 1.27E+07 | 57 | 2.25E+07 | 88 |
| AZL89458.1 | hypothetical protein Mb0706 | 3.84E+07 | 65 | 3.96E+05 | 146 | 1.22E+07 | 120 |
| AZL89124.1 | hypothetical protein Mb0354 | 1.19E+06 | 167 | 3.51E+08 | 2 | 1.19E+09 | 2 |
| AZL89479.1 | hypothetical protein Mb0729 | 5.78E+05 | 188 | 2.17E+06 | 119 | 1.16E+08 | 22 |
| AZL89478.1 | hypothetical protein Mb0728 | 3.22E+05 | 206 | 3.81E+07 | 19 | 4.46E+08 | 5 |
| AZL89580.1 | hypothetical protein Mb0171 | 2.45E+05 | 215 | 4.89E+06 | 96 | 1.35E+07 | 116 |
| AZL89250.1 | hypothetical protein Mb0488 | 1.68E+05 | 220 | 5.94E+06 | 89 | 7.28E+06 | 145 |
| AZL89180.1 | hypothetical protein Mb0415 | 1.58E+05 | 222 | 1.44E+06 | 127 | 7.28E+07 | 36 |
| AZL89359.1 | hypothetical protein Mb0603 | 1.12E+05 | 225 | 3.47E+07 | 26 | 3.38E+07 | 70 |

*Mb0671.
