# Supplementary material for: Megavirus baoshanense Mb0671 modulates host translation and increases viral fitness
Source: Front Microbiol. 2025 Apr 28;16:1574090. doi: 10.3389/fmicb.2025.1574090 (PMC12066439; doi:10.3389/fmicb.2025.1574090)
Supplement: Supplementary file 3 [file Table_3.docx]

**Supplementary Table S3. Proteins significantly enriched in different pathways**

**Table S3A. Proteins that are significantly enriched in the HIF-1 signaling pathway.**

| **Pathway: HIF-1 signaling pathway Pvalue=0.00742261** | | | |
| --- | --- | --- | --- |
| **Protein** | **Protein ID** | **Description** | **Log2(fc)** |
| ERK | L8H7P6 | Mitogenactivated protein kinase 5, putative | 7.62 |
| eIF4E | L8HF00 | Eukaryotic initiation factor, putative | 3.44 |
| ElonginB | L8GP24 | Ubiquitin family protein | 2.17 |
| AKT | L8HBH8 | Non-specific serine/threonine protein kinase | 1.8 |
| AKT | L8GIE6 | Non-specific serine/threonine protein kinase | 1.61 |
| eIF4E | L8H0E9 | Eukaryotic initiation factor 4E | 1.53 |
| ElonginC | L8GYQ9 | Elongin-C | 1.51 |
| PI3K | L8GS90 | Phosphoinositide 3kinase family, accessory domain (Pik domain) domain containing protein | 1.39 |
| mTOR | L8GIV8 | Serine/threonine-protein kinase TOR | 1.3 |
| eIF4E | L8H4Z1 | Eukaryotic initiation factor 4e, putative | 1.13 |
| eIF4E | L8GFK0 | Eukaryotic initiation factor 4E protein, putative | 1.12 |

**Table S3B. Proteins that are significantly enriched in the JAK-STAT signaling pathway.**

| **Pathway: JAK-STAT signaling pathway Pvalue=0.02713409** | | | |
| --- | --- | --- | --- |
| **Protein** | **Protein ID** | **Description** | **Log2(fc)** |
| GRB | L8HBS2 | SH3 domain containing protein | 13.65 |
| Ras | L8GU20 | RAP1A, member of RAS oncogene family | 4.17 |
| Akt | L8HBH8 | Non-specific serine/threonine protein kinase | 1.80 |
| Akt | L8GIE6 | Non-specific serine/threonine protein kinase | 1.61 |
| PI3K | L8GS90 | Phosphoinositide 3kinase family, accessory domain (Pik domain) domain containing protein | 1.39 |
| mTOR | L8GIV8 | Serine/threonine-protein kinase TOR | 1.30 |
| Ras | L8GMR5 | Ras subfamily protein | 1.24 |
| SOS | L8GSE4 | RasGEF domain containing protein (Fragment) | 1.16 |
| SOS | L8HDG6 | RasGEF domain containing protein | 1.08 |

**Table S3C. Proteins that are significantly enriched in the EGFR tyrosine kinase inhibitor resistance.**

| **Pathway: EGFR tyrosine kinase inhibitor resistance Pvalue=0.0192899** | | | |
| --- | --- | --- | --- |
| **Protein** | **Protein ID** | **Description** | **Log2(fc)** |
| Grb2 | L8HBS2 | SH3 domain containing protein | 13.65 |
| ERK | L8H7P6 | Mitogenactivated protein kinase 5, putative | 7.62 |
| Ras | L8GU20 | RAP1A, member of RAS oncogene family | 4.17 |
| Ras | L8H7T7 | Raslike protein rasG, putative | 1.90 |
| Ras | L8HF47 | Ras GTPase, putative | 1.46 |
| Ras | L8GMR5 | Ras subfamily protein | 1.24 |
| PI3K | L8GS90 | Phosphoinositide 3kinase family, accessory domain (Pik domain) domain containing protein | 1.39 |
| eIF4E | L8HF00 | Eukaryotic initiation factor, putative | 3.44 |
| eIF4E | L8H0E9 | Eukaryotic initiation factor 4E | 1.53 |
| eIF4E | L8H4Z1 | Eukaryotic initiation factor 4e, putative | 1.13 |
| eIF4E | L8GFK0 | Eukaryotic initiation factor 4E protein, putative | 1.12 |
| PTEN | L8GSP7 | Protein-tyrosine-phosphatase | 1.77 |
| Akt | L8HBH8 | Non-specific serine/threonine protein kinase | 1.80 |
| Akt | L8GIE6 | Non-specific serine/threonine protein kinase | 1.61 |
| mTOR | L8GIV8 | Serine/threonine-protein kinase TOR | 1.30 |
| SOS | L8GSE4 | RasGEF domain containing protein (Fragment) | 1.16 |
| SOS | L8HDG6 | RasGEF domain containing protein | 1.08 |

**Table S3D. Proteins that are significantly enriched in the insulin signaling pathway.**

| **Pathway: Insulin signaling pathway Pvalue=0.02518622** | | | |
| --- | --- | --- | --- |
| **Protein** | **Protein ID** | **Description** | **Log2(fc)** |
| Grb2 | L8HBS2 | SH3 domain containing protein | 13.65 |
| PKA | L8GJI9 | Serine/threonine kinase | 11.98 |
| ERK1/2 | L8H7P6 | Mitogenactivated protein kinase 5, putative | 7.62 |
| Ras | L8GU20 | RAP1A, member of RAS oncogene family | 4.17 |
| Ras | L8H7T7 | Raslike protein rasG, putative | 1.90 |
| Ras | L8HF47 | Ras GTPase, putative | 1.46 |
| Ras | L8GMR5 | Ras subfamily protein | 1.24 |
| PHK | L8HBJ0 | Calmodulin | 4.14 |
| eIF4E | L8HF00 | Eukaryotic initiation factor, putative | 3.44 |
| eIF4E | L8H0E9 | Eukaryotic initiation factor 4E | 1.53 |
| eIF4E | L8H4Z1 | Eukaryotic initiation factor 4e, putative | 1.13 |
| eIF4E | L8GFK0 | Eukaryotic initiation factor 4E protein, putative | 1.12 |
| PI3K | L8GS90 | Phosphoinositide 3kinase family, accessory domain (Pik domain) domain containing protein | 1.39 |
| PDK1/2 | L8HAN3 | 3phosphoinositide-dependent protein kinase 1, putative | 1.31 |
| Akt | L8HBH8 | Non-specific serine/threonine protein kinase | 1.80 |
| Akt | L8GIE6 | Non-specific serine/threonine protein kinase | 1.61 |
| mTOR | L8GIV8 | Serine/threonine-protein kinase TOR | 1.30 |
| Raptor | L8GIL5 | Regulatory-associated protein of mTOR, putative | 1.13 |
| PP1 | L8GV01 | Peptidylprolyl isomerase | 1.02 |
| PHK | L8GID0 | Myosin IC light chain, putative | 1.21 |
| SOS | L8GSE4 | RasGEF domain containing protein (Fragment) | 1.16 |
| SOS | L8HDG6 | RasGEF domain containing protein | 1.08 |

**Table S3E. Proteins that are significantly upregulated in the Ac_Mb0671 enriched in the mTOR signaling pathway.**

| **Pathway: mTOR signaling pathway**  Pvalue = 0.02562547 | | | |
| --- | --- | --- | --- |
| **Protein** | **Protein ID** | **Description** | **Log2(fc)** |
| Grb2 | L8HBS2 | SH3 domain containing protein | 13.65 |
| ERK1/2 | L8H7P6 | Mitogenactivated protein kinase 5, putative | 7.62 |
| Ras | L8GU20 | RAP1A, member of RAS oncogene family | 4.17 |
| Ras | L8H7T7 | Raslike protein rasG, putative | 1.9 |
| Ras | L8HF47 | Ras GTPase, putative | 1.46 |
| Ras | L8GMR5 | Ras subfamily protein | 1.24 |
| eIF4E | L8HF00 | Eukaryotic initiation factor, putative | 3.44 |
| eIF4E | L8H0E9 | Eukaryotic initiation factor 4E | 1.53 |
| eIF4E | L8H4Z1 | Eukaryotic initiation factor 4e, putative | 1.13 |
| eIF4E | L8GFK0 | Eukaryotic initiation factor 4E protein, putative | 1.12 |
| V-ATPase | L8H9V1 | Vacuolar proton pump subunit B | 1.88 |
| V-ATPase | L8H9M3 | V-type proton ATPase subunit G | 1.62 |
| V-ATPase | L8GGR7 | Vacuolar atp synthase subunit h, putative | 1.18 |
| Akt | L8HBH8 | Non-specific serine/threonine protein kinase | 1.8 |
| Akt | L8GIE6 | Non-specific serine/threonine protein kinase | 1.61 |
| PTEN | L8GSP7 | Protein-tyrosine-phosphatase | 1.77 |
| eIF4B | L8GR31 | Uncharacterized protein | 1.77 |
| PI3K | L8GS90 | Phosphoinositide 3kinase family, accessory domain (Pik domain) domain containing protein | 1.39 |
| PDK1 | L8HAN3 | 3phosphoinositide-dependent protein kinase 1, putative | 1.31 |
| mTOR | L8GIV8 | Serine/threonine-protein kinase TOR | 1.3 |
| SOS | L8GSE4 | RasGEF domain containing protein (Fragment) | 1.16 |
| SOS | L8HDG6 | RasGEF domain containing protein | 1.08 |
| GATOR2 | L8HCL0 | zinc_ribbon_16 domain-containing protein | 1.15 |
| Raptor | L8GIL5 | Regulatory-associated protein of mTOR, putative | 1.13 |

**Table S3F. Proteins that are significantly enriched in the spliceosome pathway.**

| **Pathway: Spliceosome Pvalue = 0.000273848** | | | |
| --- | --- | --- | --- |
| **Protein** | **Protein ID** | **Description** | **Log2(fc)** |
| CypE | L8GMN9 | Peptidylprolyl cis-trans isomerase e, ppie, putative | 11.69 |
| S164 | L8H642 | PWI domain containing protein | 8.15 |
| HSP73 | L8GGZ4 | Heat shock protein ssa1, putative | 3.99 |
| SR | L8GP81 | RNA recognition motif domain containing protein | 3.24 |
| SR | L8HE36 | RNA recognition motif domain containing protein | 1.65 |
| Prp22 | L8H9J2 | RNA helicase | 2.85 |
| Prp16 | L8GZ15 | PremRNA-splicing factor ATP-dependent RNA helicase PRP16, putative | 2.78 |
| U2AF | L8GMZ7 | U2 snRNP auxilliary factor, large subunit, splicing factor subfamily protein | 2.47 |
| U2AF | L8HCR8 | Zinc finger domain containing protein | 1.90 |
| THOC | L8HEE4 | Uncharacterized protein | 2.28 |
| THOC | L8GS43 | RNA recognition motif domain containing protein | 1.58 |
| Prp28 | L8H2J1 | DEAD/DEAH box helicase domain containing protein | 2.23 |
| Prp2 | L8GHC7 | Helicase conserved Cterminal domain containing protein | 2.19 |
| CTNNBL1 | L8GZ71 | DUF1716 domain-containing protein | 2.12 |
| SF3b | L8GWI1 | Splicing factor 3b subunit 1, putative | 1.87 |
| SF3b | L8H7N6 | PSP, prolinerich, putative | 1.24 |
| p68 | L8HAM3 | ATPdependent RNA helicase dbp2, putative | 1.85 |
| p68 | L8GYV4 | ATPdependent RNA helicase DBP2, putative (Fragment) | 1.28 |
| Prp31 | L8HMF9 | Putative snoRNA binding domain containing protein | 1.56 |
| Prp4 | L8HCB3 | U4/U6 small nuclear ribonucleoprotein Prp4, putative | 1.21 |
| Snu13 | L8GS62 | Ribonucloprotein | 1.14 |
| AQR | L8GQF1 | Uncharacterized protein | 1.09 |
| Prp6 | L8GYC6 | PRP1 splicing factor, Nterminal/tetratricopeptide repeat domain containing protein | 1.07 |
| Prp5 | L8GSD6 | DEAD/DEAH box helicase domain containing protein | 1.06 |
| Isy1 | L8GQP7 | Uncharacterized protein | 1.06 |
| U1A | L8HCN4 | Uncharacterized protein | 1.02 |

**Table S3G. Proteins that are significantly enriched in the ribosome biogenesis in eukaryotes signaling pathway.**

| **Pathway: Ribosome biogenesis in eukaryotes Pvalue = 0.03164604** | | | |
| --- | --- | --- | --- |
| **Protein** | **Protein ID** | **Description** | **Log2(fc)** |
| Fap7 | L8H5N4 | Adenylate kinase isoenzyme 6 homolog | 11.83 |
| Sdo1 | L8H5P7 | Uncharacterized protein | 3.81 |
| NOP4 | L8GR35 | RNA recognition motif domain containing protein | 1.27 |
| Ran | L8GF16 | GTP-binding nuclear protein | 1.17 |
| Drg1 | L8GER0 | ATPase, AAA domain containing protein | 9.18 |
| EMG1 | L8HHN9 | EMG1 nucleolar protein, putative | 1.49 |
| Rex1/2 | L8HE07 | Exonuclease | 1.17 |
| Bms1 | L8HJR5 | Ribosome biogenesis protein | 3.04 |
| Rio1 | L8H1N7 | Serine/threonine-protein kinase RIO1 | 2.46 |
| Rio2 | L8HDY7 | Non-specific serine/threonine protein kinase | 1.27 |
| LSG1 | L8H6E0 | G domain-containing protein | 2.60 |
| Imp3 | L8GU91 | 40S ribosomal protein S9 | 1.21 |
| SNU13 | L8GS62 | Ribonucloprotein | 1.14 |
| Nog1 | L8GLH2 | Nucleolar gtpbinding protein 1, putative | 1.42 |

**Table S3H. Proteins that are significantly enriched in the biosynthesis of amino acids pathway.**

| **Pathway: Biosynthesis of amino acids Pvalue=0.006745306** | | |
| --- | --- | --- |
| **Protein ID** | **Description** | **Log2(fc)** |
| L8HFC9 | Diaminopimelate epimerase | 10.40 |
| L8H9F9 | Cysteine synthase 2, putative | 9.83 |
| L8H9R0 | p-aminobenzoic acid synthase | 3.72 |
| L8H5A8 | Ornithine cyclodeaminase | 2.82 |
| L8GEY3 | Bifunctional aspartate kinase/diaminopimelate decarboxylase protein | 2.49 |
| L8GUC6 | Tryptophan synthase subunit beta, putative | 2.29 |
| L8H2F0 | Class II glutamine amidotransferase/glutamate synthase central domain containing protein | 1.96 |
| L8HA80 | Aminotransferase, class III superfamily protein | 1.95 |
| L8GI71 | S-adenosylmethionine synthase | 1.91 |
| L8GXD5 | Methionine synthase | 1.82 |
| L8H6M4 | Tryptophan synthase | 1.78 |
| L8GUI0 | 3-deoxy-7-phosphoheptulonate synthase | 1.71 |
| L8H468 | Histidinolphosphate transaminase | 1.59 |
| L8H3K6 | 6-phosphofructokinase | 1.46 |
| L8GNS4 | Phosphoribosylpyrophosphate synthetase | 1.35 |
| L8GYL8 | Protein synthetase, putative | 1.31 |
| L8H6M2 | Indole-3-glycerol phosphate synthase | 1.26 |
| L8GZ64 | Pyridoxalphosphate dependent superfamily protein | 1.22 |
| L8GS87 | Saccharopine dehydrogenase (Nad+, l-lysine forming), putative | 1.21 |
| L8H4K1 | Aconitate hydratase, mitochondrial | 1.20 |
| L8H004 | Glutamate synthase (NADH) | 1.15 |
| L8GWP2 | Homoaconitase, mitochondrial, putative | 1.04 |
| L8HE69 | Chorismate mutase | 1.03 |
| L8GTX8 | Pyruvate carboxylase | 1.02 |

**Table S3I. Proteins that are significantly enriched in the phenylalanine, tyrosine and tryptophan biosynthesis pathway.**

| **Pathway: Phenylalanine, tyrosine and tryptophan biosynthesis Pvalue=0.01907295** | | |
| --- | --- | --- |
| **Protein ID** | **Description** | **Log2(fc)** |
| L8H9R0 | p-aminobenzoic acid synthase | 3.72 |
| L8H6M2 | Indole-3-glycerol phosphate synthase | 1.26 |
| L8GUI0 | 3-deoxy-7-phosphoheptulonate synthase | 1.71 |
| L8HE69 | Chorismate mutase | 1.03 |
| L8H6M4 | Tryptophan synthase | 1.78 |
| L8GUC6 | Tryptophan synthase subunit beta, putative | 2.29 |
| L8H468 | Histidinolphosphate transaminase | 1.59 |

**Table S3J. Proteins that are significantly enriched in the autophagy-yeast pathway.**

| **Pathway: Autophagy-yeast Pvalue = 0.0313342** | | | |
| --- | --- | --- | --- |
| **Protein** | **Protein ID** | **Description** | **Log2(fc)** |
| PKA | L8GJI9 | Serine/threonine kinase | 11.98 |
| ARP2/3 | L8HJA5 | Actin-related protein 2 | 9.85 |
| ARP2/3 | L8HGQ4 | Actin-related protein 2 | 1.54 |
| ATG4 | L8H1P1 | Cysteine protease | 3.77 |
| Vps33 | L8HJ53 | Non-specific serine/threonine protein kinase | 2.77 |
| Gcn2 | L8GT53 | Sec1like family protein | 2.69 |
| Vps16 | L8H081 | Vacuolar protein sorting 16, putative | 2.59 |
| Sec17 | L8GWA7 | Tetratricopeptide repeat domain containing protein | 2.09 |
| ATG3 | L8H445 | Autophagyrelated protein 3, putative | 1.96 |
| Ras | L8H7T7 | Raslike protein rasG, putative | 1.90 |
| Ras | L8HF47 | Ras GTPase, putative | 1.46 |
| Ykt6 | L8HHL3 | Lethal (1), putative | 1.79 |
| Ypt7 | L8GRY8 | Rab7/RabGfamily small GTPase | 1.35 |
| Ypt7 | L8HGU8 | Ras family protein | 1.28 |
| Ypt7 | L8H231 | GTPbinding protein | 1.12 |
| TOR | L8GIV8 | Serine/threonine-protein kinase TOR | 1.30 |
| Raptor | L8GIL5 | Regulatory-associated protein of mTOR, putative | 1.13 |
| ATG18 | L8H3G2 | Autophagyrelated protein 18, putative (Fragment) | 1.10 |
| eIF2α | L8H146 | Eukaryotic initiation factor, putative | 1.10 |
| Ypt7 | L8HEH5 | Rab7, putative | 1.05 |

**Table S3K. Proteins that are significantly enriched in the SNARE interactions in vesicular pathway.**

| **Pathway: SNARE interactions in vesicular Pvalue=0.01619815** | | | |
| --- | --- | --- | --- |
| **Protein** | **Protein ID** | **Description** | **Log2(fc)** |
| Stxl6 | L8GY87 | Syntaxinlike t-SNARE protein TLG2, putative | 12.04 |
| Bet1 | L8GF91 | SNARE domain containing protein | 10.45 |
| Stx7 | L8HBP6 | Syntaxin7A, putative | 3.27 |
| VAMP7 | L8GS45 | Vesicle-associated membrane protein, putative | 1.97 |
| Ykt6 | L8HHL3 | Lethal (1), putative | 1.79 |
| Stxl-4 | L8HBG6 | Pescadillo homolog | 1.36 |

**Table S3L. Proteins that are significantly enriched in the DNA replication pathway.**

| **Pathway: DNA replication Pvalue=0.01397448** | | | |
| --- | --- | --- | --- |
| **Protein** | **Protein ID** | **Description** | **Log2(fc)** |
| RPA | L8H9Z0 | DEAD box RNA helicase | 4.57 |
| PCNA | L8GYX4 | Proliferating cell nuclear antigen | 3.27 |
| δ1 | L8HIY3 | DNA polymerase | 2.62 |
| Lig | L8HHC6 | DNA ligase | 2.52 |
| RFC2/4 | L8H0K7 | DNA replication factor C complex subunit 2, putative | 2.41 |
| SSB | L8HBV6 | Singlestrand binding protein family | 2.00 |
| DpoI | L8H556 | DNA-directed DNA polymerase | 1.71 |
| α1 | L8GWY4 | DNA polymerase | 1.46 |
| δ2 | L8HI26 | DNA polymerase epsilon subunit B protein | 1.26 |
| ε1 | L8GY46 | DNA polymerase epsilon catalytic subunit | 1.01 |

**Table S3M. Proteins that are significantly enriched in the mismatch repair signaling pathway.**

| **Pathway: Mismatch repair Pvalue=0.02054486** | | | |
| --- | --- | --- | --- |
| **Protein** | **Protein ID** | **Description** | **Log2(fc)** |
| Polδ | L8HI26 | DNA polymerase epsilon subunit B protein | 1.26 |
| Polδ | L8HIY3 | DNA polymerase | 2.62 |
| RFC | L8H0K7 | DNA replication factor C complex subunit 2, putative | 2.41 |
| SSB | L8HBV6 | Singlestrand binding protein family | 2.00 |
| LIG1 | L8HHC6 | DNA ligase | 2.52 |
| RPA | L8H9Z0 | DEAD box RNA helicase | 4.57 |
| PCNA | L8GYX4 | Proliferating cell nuclear antigen | 3.27 |
| MSH2 | L8GS03 | DNA mismatch repair protein msh2, putative | 1.15 |
