# Supplementary material for: Megavirus baoshanense Mb0671 modulates host translation and increases viral fitness
Source: Front Microbiol. 2025 Apr 28;16:1574090. doi: 10.3389/fmicb.2025.1574090 (PMC12066439; doi:10.3389/fmicb.2025.1574090)
Supplement: Supplementary file 4 [file Table_4.docx]

**Supplementary Table S4. Differentially expressed M. baoshan proteins in Ac_Mb0671 compared to Ac at 9 h p.i.**

| **Protein ID** | **Description** | **Log2(fc)** |
| --- | --- | --- |
| AZL89331.1 | hypothetical protein Mb0574 | 11.87 |
| UFX99792.1 | F-box and FNIP repeat-containing protein | 10.31 |
| AZL89601.1 | ankyrin repeat protein | 10.17 |
| AZL89373.1 | factor C large subunit | 9.91 |
| AZL89809.1 | hypothetical protein Mb0970 | 9.88 |
| AZL89109.1 | hypothetical protein Mb0340 | 9.78 |
| UFX99716.1 | BTB/POZ domain-containing protein | 9.69 |
| AZL89272.1 | hypothetical protein Mb0512 | 9.36 |
| AZL89873.1 | methionyl-tRNA synthetase | 9.28 |
| AZL89607.1 | hypothetical protein Mb0139 | 8.99 |
| AZL89790.1 | hypothetical protein Mb0949 | 8.93 |
| AZL89536.1 | DNA-directed RNA polymerase subunit 6 | 8.92 |
| AZL89938.1 | F-box and FNIP repeat-containing protein | 8.82 |
| AZL89276.1 | hypothetical protein Mb0516 | 8.78 |
| AZL89744.1 | ankyrin repeat protein | 8.71 |
| AZL89655.1 | hypothetical protein Mb0081 | 8.66 |
| UFX99772.1 | F-box and FNIP repeat-containing protein | 8.64 |
| AZL89789.1 | hypothetical protein Mb0948 | 8.50 |
| AZL89654.1 | hypothetical protein Mb0082 | 8.49 |
| AZL89644.1 | hypothetical protein Mb0092 | 8.19 |
| AZL89925.1 | hypothetical protein Mb0259 | 8.15 |
| AZL89299.1 | ribonuclease 3 | 7.92 |
| AZL89608.1 | BTB/POZ domain-containing protein | 7.84 |
| AZL89532.1 | hypothetical protein Mb0787 | 7.72 |
| AZL90014.1 | ankyrin repeat protein | 7.71 |
| AZL89858.1 | BTB/POZ domain-containing protein | 7.69 |
| UFX99813.1 | hypothetical protein Mb0471 | 7.49 |
| AZL89540.1 | hypothetical protein Mb0796 | 7.32 |
| AZL89372.1 | core protein | 7.22 |
| AZL89653.1 | ankyrin repeat protein | 7.17 |
| AZL89442.1 | hypothetical protein Mb0689 | 6.62 |
| UFX99757.1 | ankyrin repeat protein | 6.47 |
| AZL89792.1 | ankyrin repeat protein | 6.17 |
| AZL89657.1 | BTB/POZ domain-containing protein | 3.68 |
| AZL89771.1 | hypothetical protein Mb0927 | 3.39 |
| AZL89490.1 | isoleucyl-tRNA synthetase,ankyrin repeat protein | 3.05 |
| AZL89495.1 | hypothetical protein Mb0745 | 2.77 |
| AZL89482.1 | ATP-dependent RNA helicase | 2.62 |
| AZL89756.1 | NUDIX hydrolase domain-containing protein | 2.48 |
| AZL89918.1 | hypothetical protein Mb0266 | 2.42 |
| AZL89499.1 | hypothetical protein Mb0751 | 1.99 |
| AZL89747.1 | ankyrin repeat protein | 1.96 |
| AZL89658.1 | ankyrin repeat protein | 1.81 |
| UFX99753.1 | hypothetical protein Mb0179 | 1.78 |
| AZL89342.1 | hypothetical protein Mb0585 | 1.72 |
| AZL89972.1 | hypothetical protein Mb0835 | 1.72 |
| UFX99827.1 | replication factor C small subunit | 1.67 |
| AZL89504.1 | DNA-directed RNA polymerase subunit 2 | 1.66 |
| AZL90012.1 | hypothetical protein Mb0226 | 1.59 |
| AZL89429.1 | hypothetical protein Mb0676 | 1.59 |
| AZL89769.1 | hypothetical protein Mb0924 | 1.56 |
| AZL89409.1 | J domain-containing protein | 1.56 |
| AZL89433.1 | hypothetical protein Mb0680 | 1.42 |
| AZL89305.1 | hypothetical protein Mb0547 | 1.27 |
| AZL89210.1 | DNA helicase | 1.27 |
| AZL89459.1 | hypothetical protein Mb0708 | 1.26 |
| AZL89347.1 | hypothetical protein Mb0590 | 1.23 |
| AZL89906.1 | cysteinyl-tRNA synthetase | 1.20 |
| AZL89258.1 | protein phosphatase 2c | 1.16 |
| AZL89835.1 | ankyrin repeat protein | 1.15 |
| AZL89438.1 | DNA directed RNA polymerase subunit | 1.07 |
| AZL89293.1 | cullin,ankyrin repeat protein | 1.04 |
| AZL89576.1 | ankyrin repeat protein | 1.04 |
| UFX99908.1 | hypothetical protein Mb0990 | -9.41 |
| AZL89217.1 | hypothetical protein Mb0453 | -7.64 |
| AZL89432.1 | hypothetical protein Mb0679 | -2.98 |
| UFX99826.1 | ubiquitin | -2.55 |
| AZL89910.1 | hypothetical protein Mb0275 | -2.35 |
| AZL89682.1 | ankyrin repeat protein | -2.04 |
| UFX99880.1 | hypothetical protein Mb0875 | -2.00 |
| AZL89403.1 | capsid protein | -1.81 |
| AZL89915.1 | ankyrin repeat protein | -1.69 |
| AZL89295.1 | transcription factor S-II-related protein | -1.56 |
| AZL89795.1 | ankyrin repeat protein | -1.48 |
| AZL89527.1 | hypothetical protein Mb0782 | -1.46 |
| AZL89250.1 | hypothetical protein Mb0488 | -1.30 |
| AZL89391.1 | transcription factor | -1.29 |
| AZL89557.1 | hypothetical protein Mb0196 | -1.26 |
| AZL89288.1 | zinc finger MynD domain-containing protein | -1.23 |
| AZL89916.1 | ankyrin repeat protein | -1.15 |
| AZL89986.1 | Cu/Zn superoxide dismutase | -1.03 |
| UFX99732.1 | hypothetical protein Mb0100 | -1.01 |
