# Supplementary material for: Megavirus baoshanense Mb0671 modulates host translation and increases viral fitness
Source: Front Microbiol. 2025 Apr 28;16:1574090. doi: 10.3389/fmicb.2025.1574090 (PMC12066439; doi:10.3389/fmicb.2025.1574090)
Supplement: Supplementary file 5 [file Table_5.docx]

**Supplementary Table S5. Differentially expressed M. baoshan proteins in siRNA_Mb0671 cells compared to NC cells at 4 h p.i.**

| Protein ID | Description | Log2(fc) |
| --- | --- | --- |
| AZL89993.1 | CfxQ-like protein | 10.46 |
| UFX99827.1 | replication factor C small subunit | 9.14 |
| AZL89760.1 | BTB/POZ domain-containing protein | 7.80 |
| AZL89555.1 | hypothetical protein Mb0199 | 7.66 |
| AZL89495.1 | hypothetical protein Mb0745 | 6.79 |
| AZL89276.1 | hypothetical protein Mb0516 | 6.52 |
| AZL89352.1 | hypothetical protein Mb0596 | 6.07 |
| AZL89215.1 | hypothetical protein Mb0451 | 6.03 |
| AZL89307.1 | hypothetical protein Mb0549 | 5.78 |
| AZL89353.1 | hypothetical protein Mb0597 | 5.77 |
| UFX99779.1 | hypothetical protein Mb0255 | 5.41 |
| AZL89467.1 | eukaryotic translation initiation factor 4e-like protein | 3.30 |
| AZL89906.1 | cysteinyl-tRNA synthetase | 3.14 |
| AZL89504.1 | DNA-directed RNA polymerase subunit 2 | 2.01 |
| AZL89464.1 | hypothetical protein Mb0713 | 1.95 |
| AZL89946.1 | F-box and FNIP repeat-containing protein | 1.86 |
| AZL89266.1 | ribonucleoside-diphosphate reductase large subunit | 1.81 |
| AZL89551.1 | glycosyltransferase | 1.79 |
| AZL89894.1 | ankyrin repeat protein | 1.71 |
| AZL89470.1 | replication factor C small subunit | 1.62 |
| AZL89372.1 | core protein | 1.53 |
| AZL89318.1 | glycosyltransferase | 1.45 |
| AZL89273.1 | DNA polymerase family X protein | 1.44 |
| AZL89642.1 | serine/threonine protein kinase receptor | 1.35 |
| AZL89769.1 | hypothetical protein Mb0924 | 1.35 |
| AZL89129.1 | ankyrin repeat protein | 1.21 |
| AZL89598.1 | purine phosphorylase,ankyrin repeat protein | 1.18 |
| AZL89607.1 | hypothetical protein Mb0139 | 1.17 |
| AZL89409.1 | J domain-containing protein | 1.16 |
| UFX99824.1 | dTDP-d-glucose 4 6-dehydratase | 1.11 |
| AZL89415.1 | viral transcription factor 2 | 1.10 |
| AZL89842.1 | hypothetical protein Mb1007 | 1.04 |
| AZL89297.1 | poly(A) polymerase catalytic subunit | -9.40 |
| AZL89250.1 | hypothetical protein Mb0488 | -9.03 |
| AZL89151.1 | methyltransferase FkbM family protein | -8.41 |
| AZL89341.1 | hypothetical protein Mb0584 | -8.17 |
| AZL89923.1 | serine/threonine-protein kinase | -7.80 |
| AZL89644.1 | hypothetical protein Mb0092 | -7.39 |
| AZL89954.1 | hypothetical protein Mb0854 | -6.94 |
| AZL89141.1 | hypothetical protein Mb0371 | -6.68 |
| AZL89965.1 | hypothetical protein Mb0842 | -6.42 |
| UFX99837.1 | replication factor C small subunit | -5.91 |
| AZL89324.1 | ATP-dependent RNA helicase | -5.75 |
| AZL89458.1 | hypothetical protein Mb0706 | -5.29 |
| AZL89537.1 | DNA-dependent RNA polymerase subunit rpb9 | -5.01 |
| AZL89330.1 | hypothetical protein Mb0573 | -4.56 |
| AZL89424.1 | translation initiation factor 4a | -2.95 |
| AZL89444.1 | AAA family ATPase | -1.92 |
| AZL89335.1 | glycosyltransferase | -1.84 |
| UFX99849.1 | intein-containing DNA-directed RNA polymerase subunit 2 | -1.67 |
| UFX99712.1 | hypothetical protein Mb0021 | -1.62 |
| AZL89356.1 | HD domain-containing protein | -1.59 |
| AZL89636.1 | BTB/POZ domain-containing protein | -1.58 |
| AZL89580.1 | hypothetical protein Mb0171 | -1.48 |
| AZL89763.1 | serine/threonine-protein kinase | -1.42 |
| AZL89685.1 | hypothetical protein Mb0044 | -1.38 |
| AZL89210.1 | DNA helicase | -1.31 |
| AZL89206.1 | hypothetical protein Mb0442 | -1.25 |
| AZL89601.1 | ankyrin repeat protein | -1.17 |
| UFX99828.1 | hypothetical protein Mb0624 | -1.14 |
| AZL89348.1 | endonuclease of the xpg family | -1.11 |
| AZL89652.1 | hypothetical protein Mb0084 | -1.01 |
