# Supplementary figures and images for: Megavirus baoshanense Mb0671 modulates host translation and increases viral fitness
Source: Front Microbiol. 2025 Apr 28;16:1574090. doi: 10.3389/fmicb.2025.1574090 (PMC12066439; doi:10.3389/fmicb.2025.1574090)

**Supplementary Figure S1. Amino acid sequence alignment of Mb0671 and AceIF4A.**


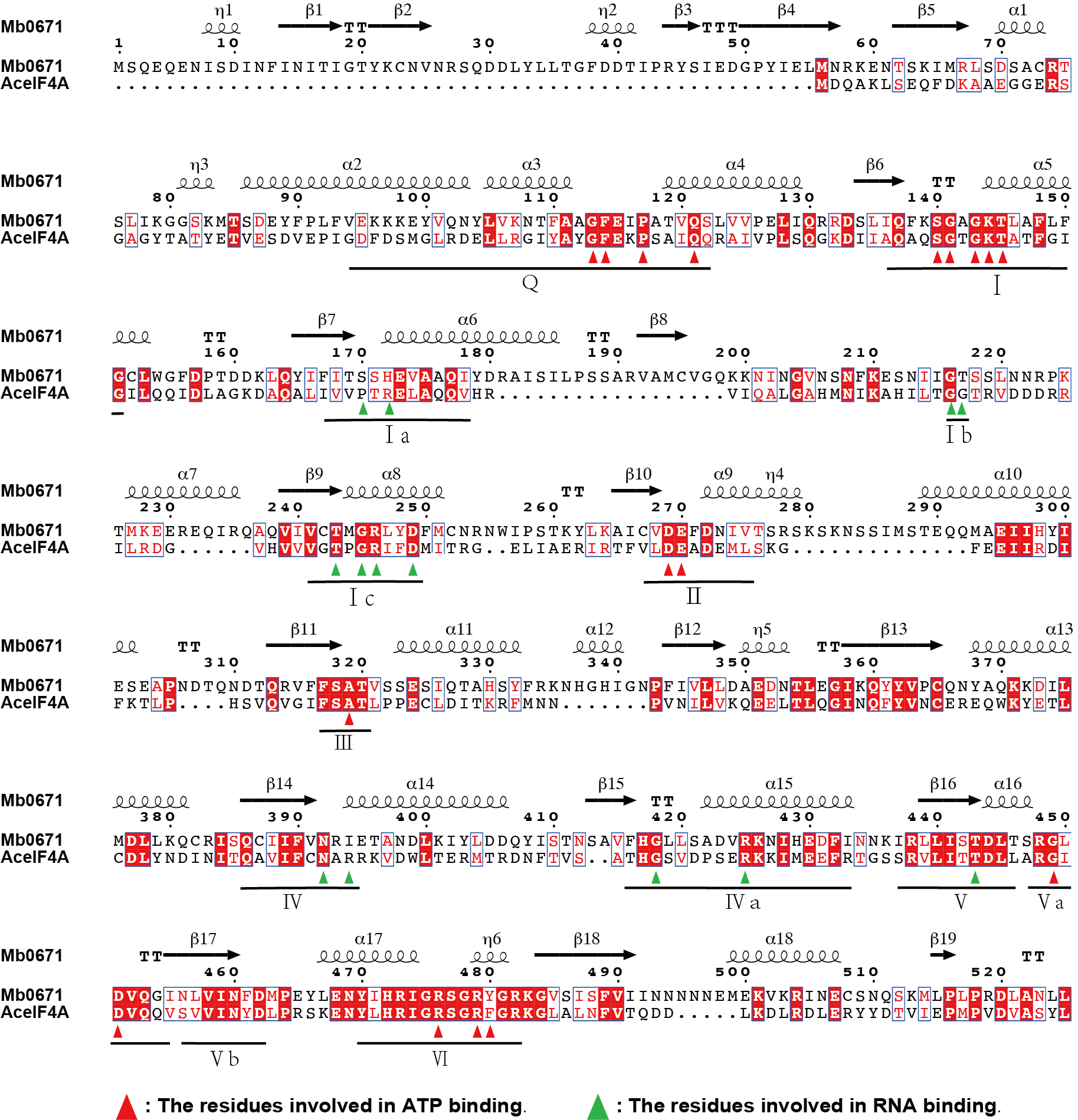

Supplement: Supplementary file 6 [file Table_6.docx]

**S1 Figure. Amino acid sequence alignment of Mb0671 with AceIF4A.**


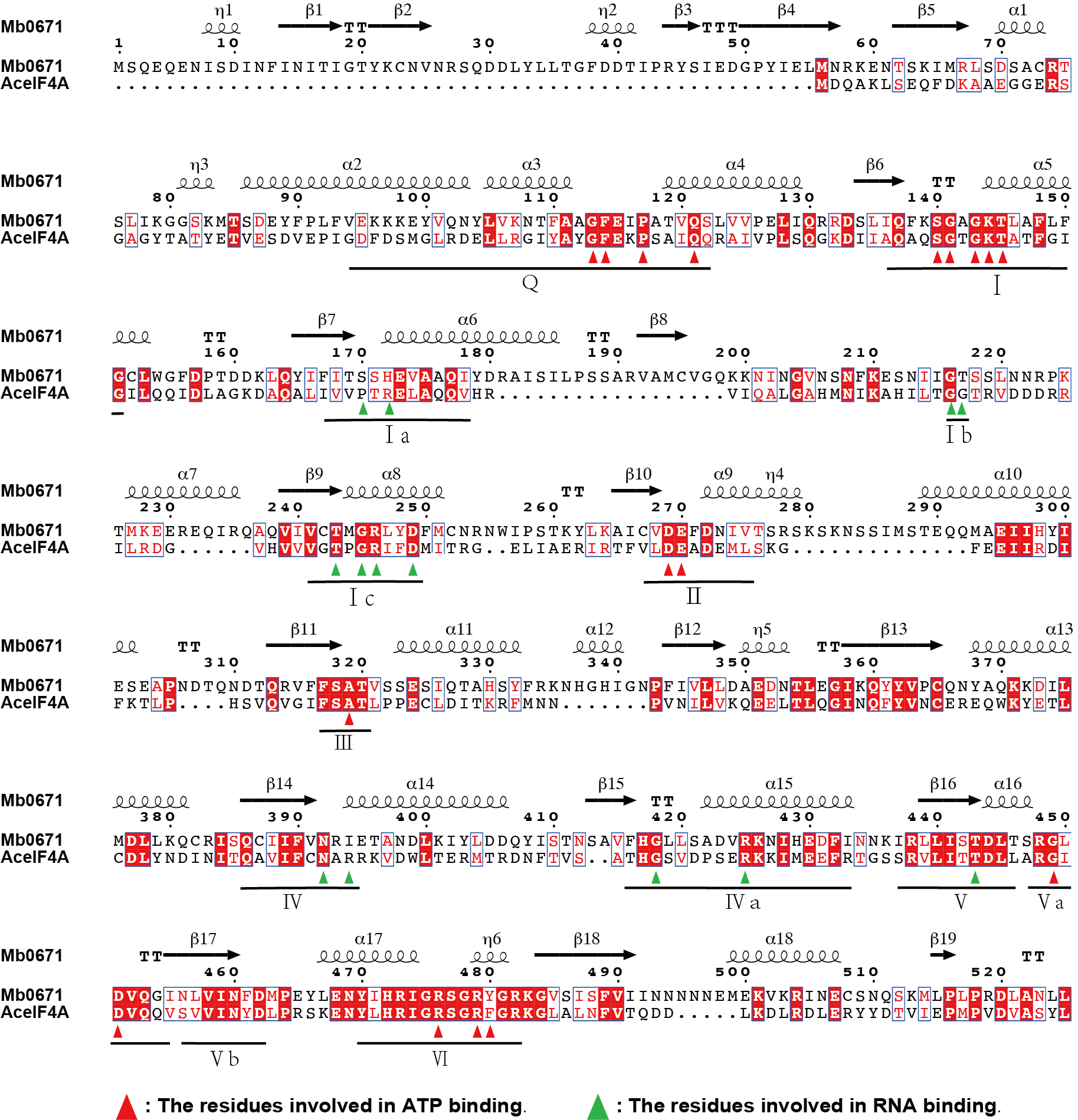

Supplement: Supplementary file 7 [file Supplementary_file_1.docx]
