# Supplementary material for: Megavirus baoshanense Mb0671 modulates host translation and increases viral fitness
Source: Front Microbiol. 2025 Apr 28;16:1574090. doi: 10.3389/fmicb.2025.1574090 (PMC12066439; doi:10.3389/fmicb.2025.1574090)

**S1 Figure. Upregulated proteins in different pathways**

**Figure A. Upregulated proteins in mTOR signaling pathway.** The red box represented proteins significantly upregulated (|Log2(Fold Change) | >1 and p-value < 0.05) in Ac_Mb0671 compared to Ac.


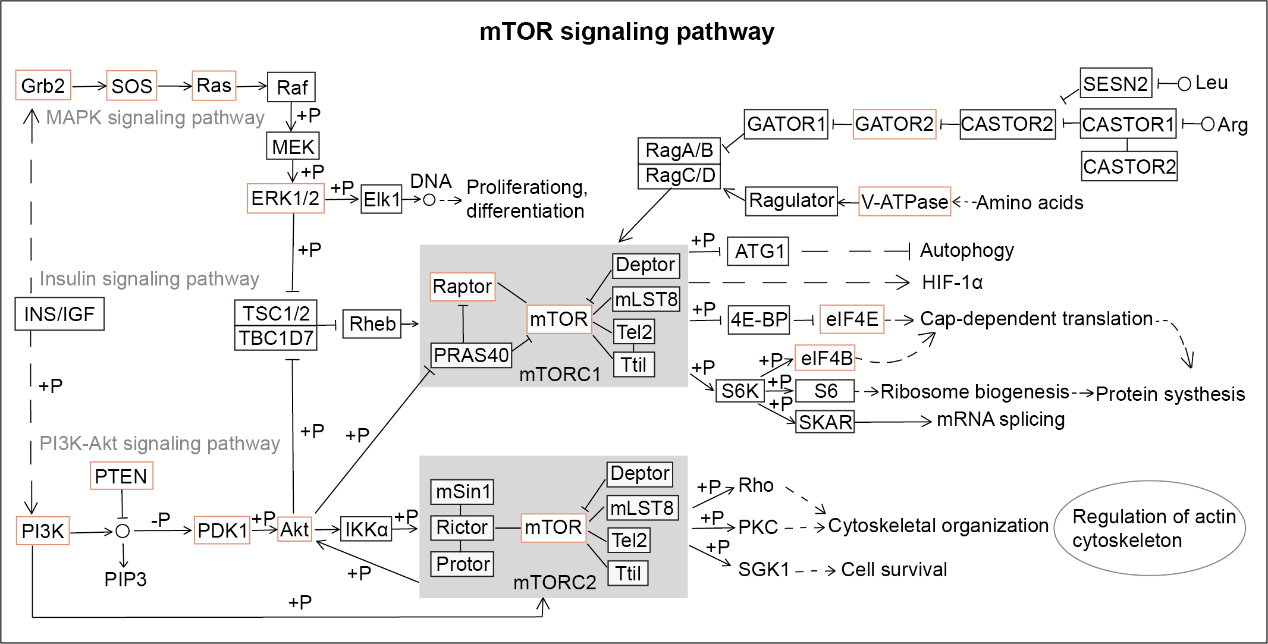


**Figure B. Upregulated proteins in splicesome pathway.** A Simplified diagram of spliceosome pathway, with red boxes represented proteins significantly upregulated (|Log2(Fold Change) | >1 and p-value < 0.05) in Ac_Mb0671 compared to Ac.


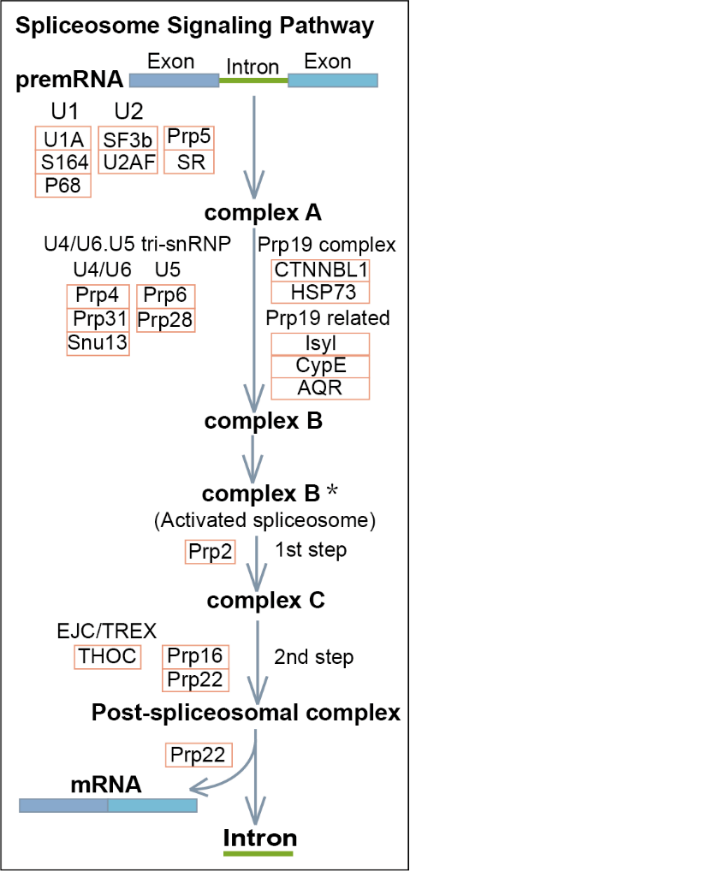


**Figure C. Upregulated proteins in ribosome biogenesis in eukaryotes.** A diagram of simplified ribosome biogenesis pathway, with red boxes represented proteins significantly upregulated (|Log2(Fold Change) | >1 and p-value < 0.05) in Ac_Mb0671 compared to Ac.


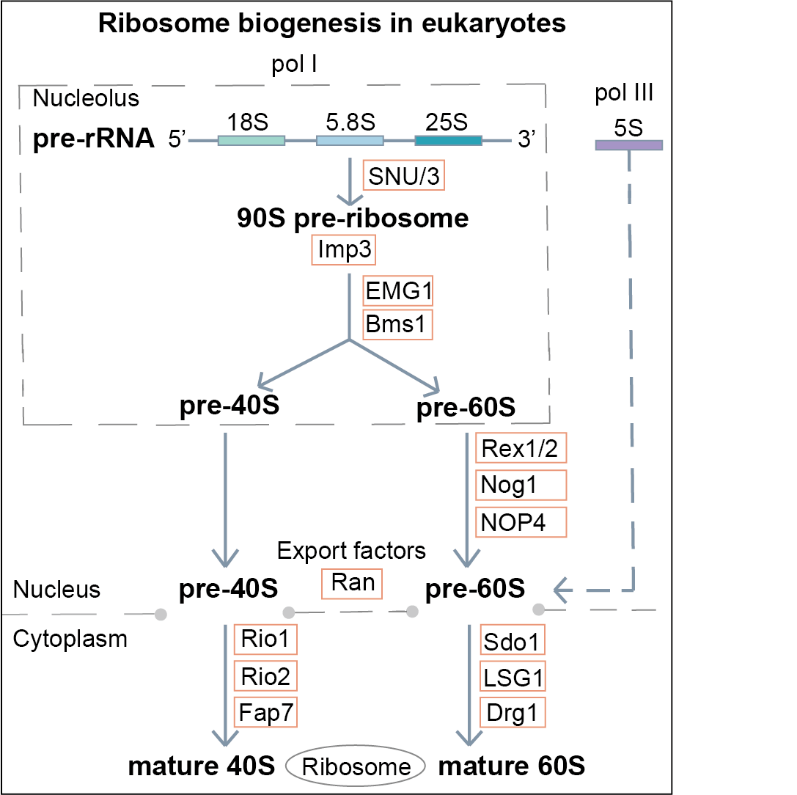


**Figure D. Upregulated proteins in autophage-yeast pathway.** A diagram of simplified autophagy-yeast pathway with red boxes represented proteins significantly upregulated (|Log2(Fold Change)| >1 and p-value < 0.05) proteins in Ac_Mb0671 compared to Ac.


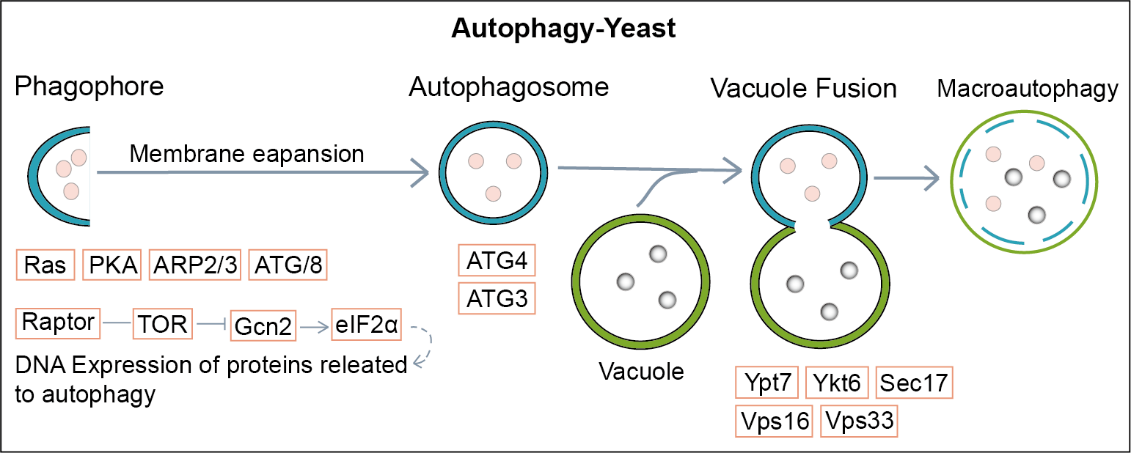


**Figure E. Proteins upregulated in SNARE Interactions.** A simplified diagram of SNARE interactions in vesicular transport, with red boxes represented proteins significantly upregulated (|Log2(Fold Change) | >1 and p-value < 0.05) in Ac_Mb0671 compared to Ac.


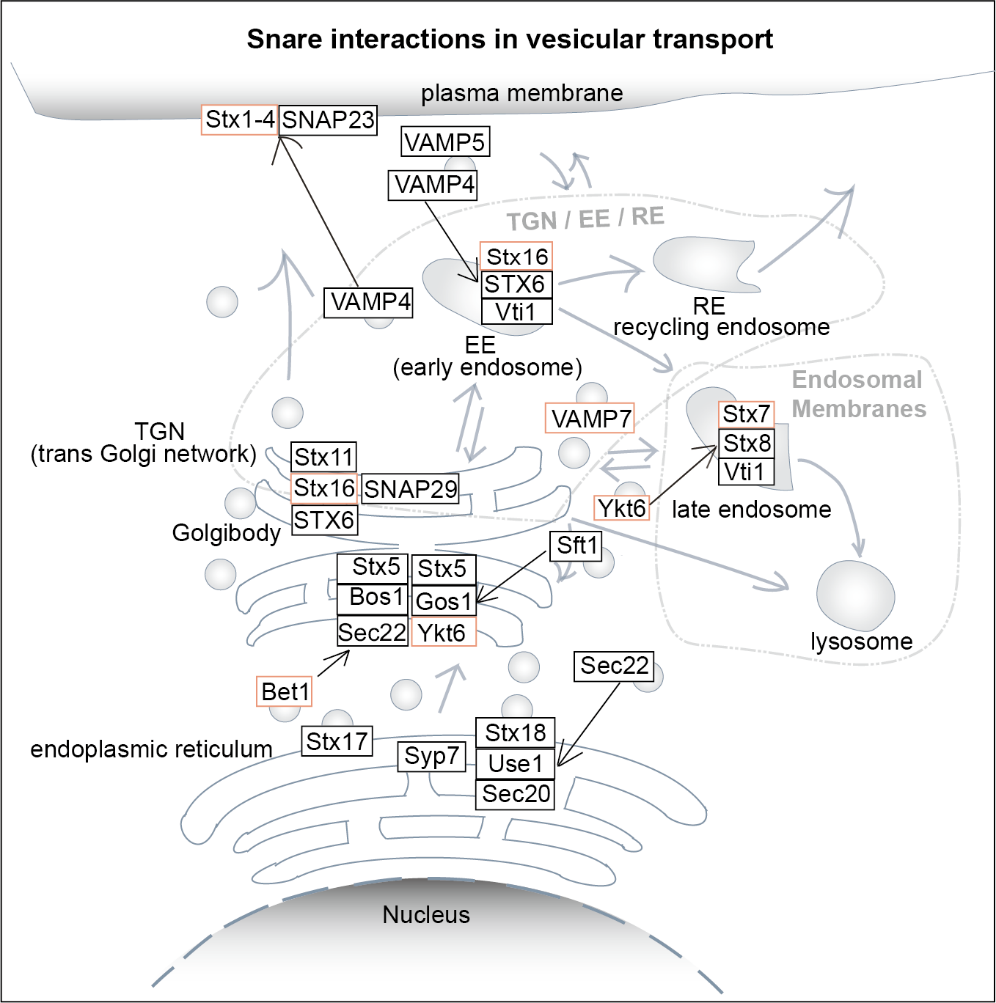

Supplement: Supplementary file 8 [file Supplementary_file_2.docx]
